# Design, Synthesis and Evaluation of the First 2-Alkynyl(aza)indole ^18^F Probe Targeting α-Synuclein Aggregates

**DOI:** 10.3390/ph18111638

**Published:** 2025-10-29

**Authors:** Liliana Boiaryna, Laura Pieri, Sylvie Chalon, Sophie Serrière, Sylvie Bodard, Gabrielle Chicheri, Elisa Chenaf, Franck Suzenet, Ronald Melki, Frédéric Buron, Sylvain Routier, Johnny Vercouillie

**Affiliations:** 1 Université d’Orléans, CNRS, ICOA, UMR 7311, 45067 Orléans, Franceelisa.chenaf@univ-orleans.fr (E.C.); franck.suzenet@univ-orleans.fr (F.S.); 2 UMR 1253, iBrain, Université de Tours, Inserm, 37044 Tours, France; laura.pieri@i2bc.paris-saclay.fr (L.P.); sylvie.chalon@univ-tours.fr (S.C.); sophie.serriere@univ-tours.fr (S.S.); sylvie.bodard@univ-tours.fr (S.B.); gabrielle.chicheri@univ-tours.fr (G.C.); johnny.vercouillie@univ-tours.fr (J.V.); 3 Paris-Saclay Institute of Neuroscience, CNRS UMR 9197, Université Paris-Saclay, 91190 Gif-sur-Yvette, France; ronald.melki@cnrs.fr

**Keywords:** alkynyl(aza)indoles, α-synuclein, Parkinson’s disease, PET

## Abstract

**Background/Objectives**: The role of α-synuclein (α-syn) in the pathogenesis of Parkinson’s disease (PD) or neurodegenerative diseases such as Lewy body dementia (LBD) and multiple system atrophy (MSA) is commonly accepted. Through different physiological dysfunctions, abnormal forms of α-syn are generated. These abnormal aggregates accumulate and alter pre- and postsynaptic transmission, in particular that of dopamine. Thus, the development of a diagnostic biomarker of synucleinopathies remains crucial and challenging. The development of an α-syn positron emission tomography (PET) radiopharmaceutical may be suitable to early diagnose and stratify patients, follow up disease progression, and evaluate future therapies. **Methods**: To develop a selective α-syn PET tracer, we synthesized an original series based on alkynyl(aza)indoles. Fifteen final ligands were synthesized bearing indoles or azaindoles from one side of the alkyne and a substituted phenyl ring for the opposite side of the alkyne. The final ligands were tested to determine Ki and/or Kd toward α-syn, tau, and Aβ. **Results**: The SAR showed that the indole series exhibited moderate to low affinity for α-syn and, moreover, lower Ki toward Aβ and tau (i.e., compound **39**, Ki_(αsyn)_ 21.7 nM, Ki_(Aβ)_ 64.4 nM, Ki_(Tau)_ 27.6 nM), highlighting the low potency of these series to afford an α-syn tracer. The introduction of a nitrogen on the different positions of the phenyl to obtain the corresponding azaindoles resulted for most of the compounds in better affinity for α-syn and selectivity towards Aβ compared to the indole analogs (i.e., compound **43**, Ki_(αsyn)_ 4.7 nM, Ki_(Aβ)_ 24.4 nM, and Ki_(Tau)_ 4.61 nM). A fluorinated azaindole derivative was prepared with a view to obtaining a ^18^F tracer and exhibited the highest affinity for α-syn but without selectivity against tau and Aβ. The radiosynthesis of [^18^F]**45** was performed in a two-step procedure starting from the tosylated and protected precursor. [^18^F]**45** was obtained in 85 ± 5 min with a radiochemical yield of 32 ± 3%. Molar activity, determined from a calibration with stable **45**, was around 130 GBq/µmole. The dynamic PET imaging showed that [^18^F]**45** was able to cross the blood–brain barrier, but non-specific uptake was observed, confirming the in vitro results. **Conclusions**: Although promising nanomolar affinity for the target, the new tracer showed mainly non-specific in vivo uptake in the rat brain, indicating that further pharmacomodulations on the azaindole series are required.

## 1. Introduction

Positron emission tomography (PET) is a unique tool for the in vivo exploration of neurodegenerative diseases characterized by the aberrant deposition of specific protein species. Significant progress has been made in developing a number of PET imaging tracers for distinctive protein aggregates of Alzheimer’s disease (AD), i.e., β-amyloid (Aβ) plaques [1] and hyperphosphorylated tau [2], which are increasingly used in clinical applications. In contrast, the detection of in vivo α-synuclein (α-syn) aggregates is still an unmet clinical need [3,4]. These pathologic aggregates, which accumulate with time, are the main features of synucleinopathies that include Parkinson’s disease (PD), dementia with Lewy bodies (DLB) and multiple system atrophy (MSA) [5]. PD affects around 6.3 million people worldwide, is the second most common age-related neurodegenerative disorder after AD and is expected to increase due to an aging population. The discovery that α-syn aggregates traffic between brain cells and grow by recruitment of endogenous α-syn suggests that pathogenesis starts many years before the first symptoms appear [6]. A PET imaging probe targeting specifically α-syn aggregates would therefore be highly useful to improve the early differential diagnosis and to monitor disease progression and the potential effects of new disease-modifying treatments in synucleinopathies.

**Figure 1:**
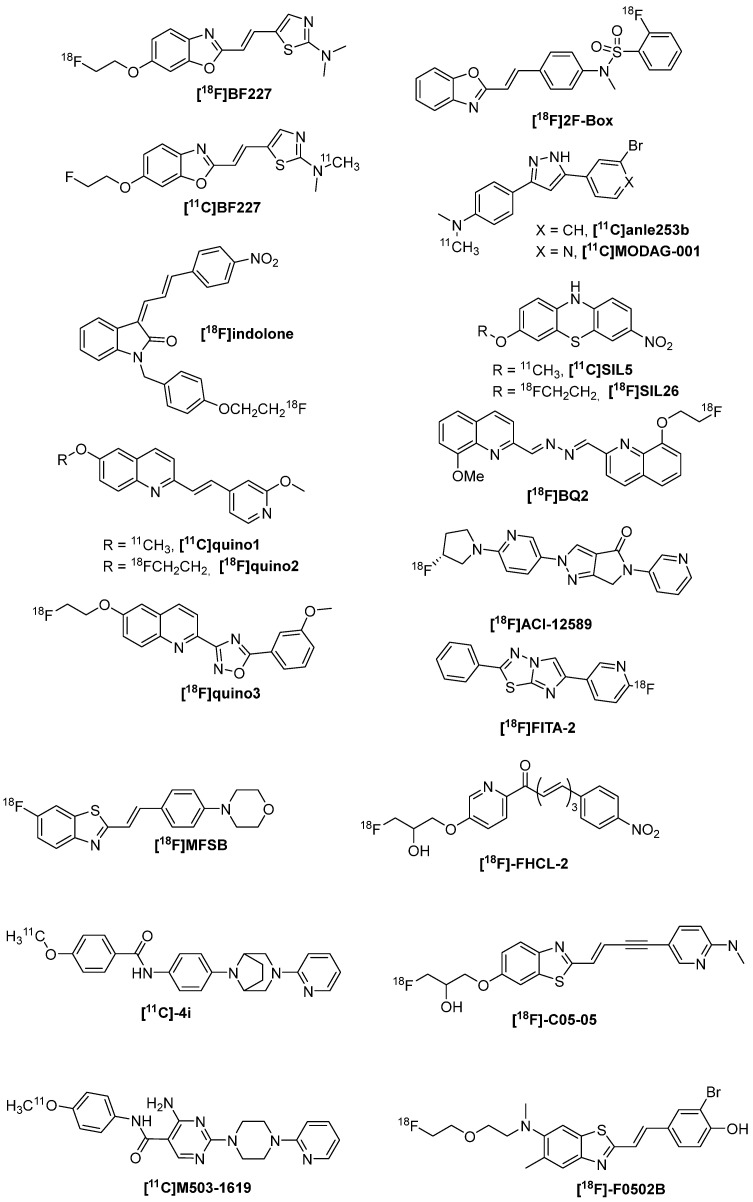
Main radiolabeled [^18^F] and [^11^C] PET tracers targeting α-synuclein aggregates [9].

Several attempts have already been made to obtain specific pathogenic α-syn binders, but with very limited success (Figure 1) [7,8,9]. Furthermore, discrepancies were often observed between rather promising results obtained using computational modeling or in vitro binding experiments on synthetic fibrils and results obtained in animal models or human brain tissue. The benzoxazole derivative **BF227** labeled with [^11^C] was first described as a promising PET tracer of Aβ plaques [10]. Then, its [^18^F]-labeled derivative was shown to also bind at least in vitro to α-syn [11], but finally did not enable α-syn aggregates to be detected in vivo in a transgenic mouse model expressing α-syn [12].

In the same chemical series, the vinyl benzoxazole derivative **[^18^F]2FBox** has been shown to bind in vitro to α-syn fibrils with a better affinity (Kd = 3.3 nM) than to Aβ (Kd = 145 nM), but despite these promising properties, it was unable to detect in vivo α-syn and Aβ either in rodent models or on human pathological brain sections [13]. More recently, a derivative of diphenylpyrazole, **anle253b**, first identified as an inhibitor of prion protein and α-syn aggregation, was labeled with [^11^C] and shown to bind in vitro to α-syn fibrils with a nanomolar affinity and to cross the blood–brain barrier in rats [14]. Structural modifications of this compound using a phenyl/pyridine bio-isostere yielded the **[^11^C]MODAG-001** which exhibited in vitro around 20 times better affinity for α-syn as compared to Aβ and tau fibrils. This tracer was able to reach in vivo α-syn fibrils inoculated in the rat brain, but gave disappointing results with very low binding on pathological human brain sections [15]. The bis-vinylindolone core gave high affinity compounds and ^18^F radiolabeling led to a **[^18^F]indolone** which had excellent in vitro properties, with a Ki of 10 nM for α-syn and a 27- and 5-fold selectivity towards Aβ and tau fibrils, respectively. It was nonetheless unsuitable for in vivo brain imaging due to its high lipophilicity [16].

An extensive structure–activity relationship study based on the tricyclic phenothiazine family resulted in selecting **SIL5** and **SIL26**, which had a moderate 30–50 nM affinity for α-syn [17] and were 2–3 fold selective towards Aβ and tau fibrils [18]. Their radiolabeled analogs **[^11^C]SIL5** and **[^18^F]SIL26** were able to cross the blood–brain barrier with rapid wash-out in the rat and non-human primate, but their properties need to be improved before application in human PET exploration [19]. More recently, the quinolein core was used to design putative PET radiotracers for α-syn. After a large screening assay on 44,000 compounds, the bis quinolinyl derivative **[^18^F]BQ2** was selected because of its good affinity (Ki around 10 nM for α-syn), but disappointing results were obtained in terms of selectivity (same Ki for Aβ) and brain uptake in the mouse [20]. This uptake was improved with the chalcone analog **[^18^F]FHCL-2**, which has not yet reached the required properties for human application [21]. Recently, new developments of benzamide analogs led to **[^11^C]4i** which, with promising in vivo kinetics in the non-human primate (NHP) brain, could be useful for α-syn imaging in the limited case of MSA because of its probable high binding to tau aggregates [22]. The most promising results were obtained with benzothiazole derivatives **[^18^F]MFSB [23]** and **[^18^F]F0502B**, which have several positive characteristics in terms of affinity to α-syn (Kd around 10 nM) and selectivity towards Aβ and tau fibrils (both Kd around 100 nM), binding to human α-syn in vitro and in vivo in the NHP brain expressing the human target [24]. However, they still need to be evaluated in clinical assays. In 2023, the tracer **[^18^F] ACI-12589** was found to present distinct uptake in the cerebellar white matter or peduncles of multiple system atrophy patients consistent with α-syn pathology [25]. Its favorable Kd value of 22–30 nM in MSA tissues prompted several groups to pursue medicinal chemistry investigations, and imidazo[2,1-*b*][1,3,4]thiadiazole **[^18^F]FITA-2** was reported in 2024 [26].

**Figure 2:**
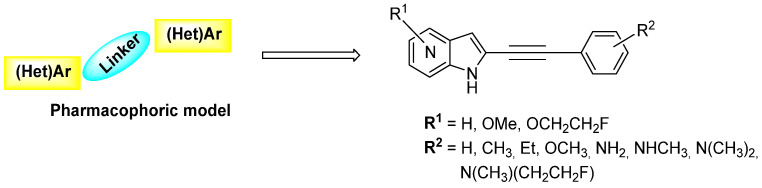
Pharmacophoric model leading to α-syn ligands and the envisioned 2-alkynyl(aza)indole family.

Apart from the alkyloxy or alkylamino chains carrying the radioelement, the structural cores of α-syn probes are mostly planar. They contain several (hetero)aromatic cycles, which are mainly linked together by π-conjugated linkers of alkenes, diazo, or small five-chain heterocycles. In addition, we noticed the weak presence of labile protons able to generate strong hydrogen bonds. With this description of the pharmacophoric model, we decided to combine an (aza)indole with an alkynyl fraction to evaluate the impact of rod-shaped molecules containing a H bond donor towards α-syn binding vs. Aβ and tau fibrils. We describe here the synthesis and in vitro evaluation of these new compounds (Figure 2) and then the preparation of one promising [^18^F]-labeled derivative for preliminary in vivo evaluation in the rat.

## 2. Results and Discussion

**Scheme 1:**
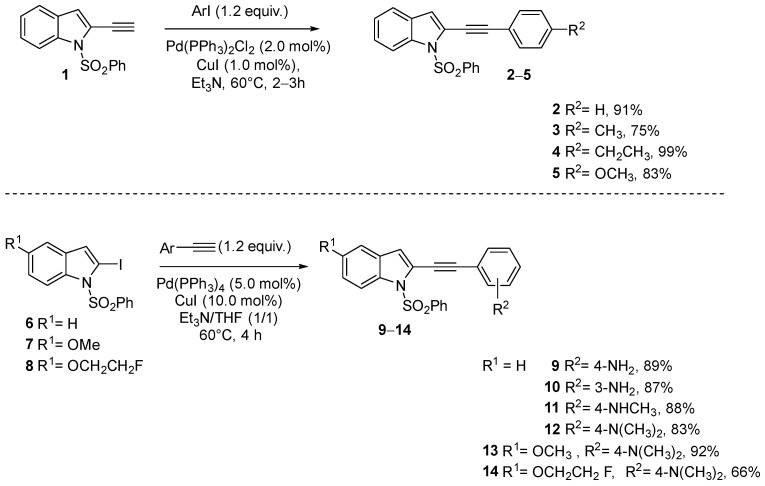
Synthetic pathways to access 2-alkynylindole derivatives.

As indicated in the literature, indole derivatives **31**–**34** can be obtained using a Sonogashira palladium-catalyzed reaction from **1** and the corresponding iodo benzenes (Scheme 1, Table 1) and after benzene sulfonyl deprotection in basic media [27,28]. To diversify access to the targeted molecules and prevent failure, we chose another method, which consisted of the cross-coupling reaction of several arylated alkynes with 2-iodo indoles **6**–**8** [29]. Derivatives **9**–**14** were readily obtained in 65–92% yields (Scheme 1).

**Scheme 2:**
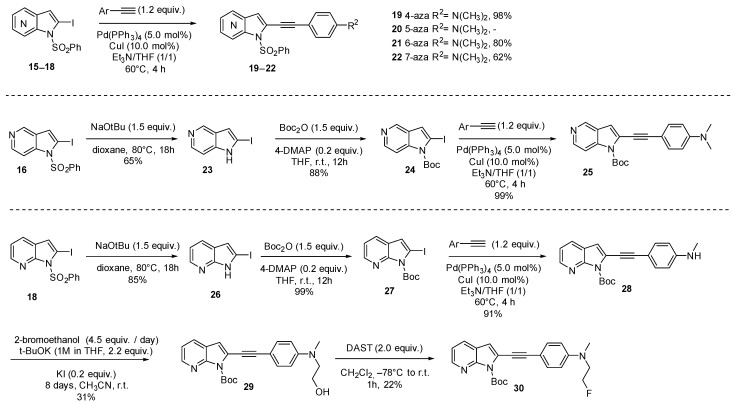
Synthesis of azaindoles derivatives.

Next, we applied this strategy to 2-iodo-azaindoles **15**–**18**, which were prepared using our procedures (Scheme 2) [30,31]. While derivatives **19** and **21** were purified and obtained in very high yield, a decrease in efficiency was observed starting with 7-aza indole compound **22**, and the 5-azaindolic derivative **20** was never obtained. We therefore replaced the benzenesulfonyl protection with a Boc group, and after two additional steps, the Sonogashira cross-coupling reaction afforded the attempted 2-alkynylindole **25** in a near quantitative yield (Scheme 2). This strategy was successfully used starting from **18** to modify the dimethyl amine group. After three steps, **28** was generated, and the 2-bromoethanol side chain was introduced by nucleophilic substitution using *t*-BuOK as a base in the presence of KI to yield **29** in 31% yield, whereas a further electrophilic fluorination with DAST led to **30** in 26% yield after 20 min of reaction [32].

**Scheme 3:**
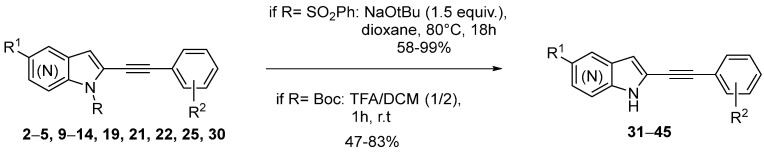
Cleavage of protecting groups.

As a last step, the indolic protective groups were removed in basic or acidic media to generate a library of 15 final compounds, which were evaluated for their binding to protein fibrils in vitro (Scheme 3).

### 2.1. In Vitro Binding Experiments

First, the affinity and selectivity of the new compounds were evaluated using competition experiments with thioflavin T (ThT). The fluorescence of ThT upon binding to α-syn, Aβ, or tau fibrils was measured in the presence of increasing concentrations of each compound, and the Ki values were calculated from IC_50_. The results (Table 1) showed that compounds **31** and **32** had a moderate to good affinity for Aβ and tau fibrils, respectively, but not for α-syn. The modification of R_2_ on the phenyl ring in the *C*-4 position led to an improvement in the affinity towards α-syn fibrils (**33**, **34**, **35**, **37**, and **39**) but not in the C-3 position (**36**). As the final aim was to obtain a [^18^F]-labeled tracer, we prepared compound **40** and observed that it was not a good candidate because of a rather good affinity for Aβ and tau fibrils (around 10–20 nM), but not for α-syn (>100 nM).

In view of the interesting properties of compounds **33**–**39**, we decided to switch to azaindole structures and to study the impact of the position of the nitrogen atom in the 6-membered ring on binding to fibrils. At this step we measured, in addition to the Ki values, the direct binding of each compound to fibrils through the fluorescence in the fraction bound to fibrils, which allowed us to determine the affinity values (Kd, Table 1). The 4 compounds (**41**, **42**, **43**, and **44**) were found to have an overall high to very high affinity for the three types of fibrils (Kd around 12–0.1 nM). The best selectivity was obtained for compound **43**, with sub-nanomolar affinity for both α-syn and tau fibrils, but Aβ showed a 20-fold reduction. We therefore prepared a fluorinated derivative of compound **43**, i.e., **45**. This structural modification led to a loss of selectivity, with similar high affinity of this compound for the 3 types of fibrils (Kd of 2.4 ± 1, 1.2 ± 1 and 0.97 ± 0.4 nM for α-syn, Aβ and tau, respectively). However, the measurement of Bmax values showed a preferential binding to our targets with 141.1 ± 45 nmol/µmol α-syn fibrils, 1.8 ± 0.6 nmol/µmol Aβ fibrils and 46.6 ± 5.0 nmol/µmol tau fibrils corresponding to 1 compound bound per 7 α-syn monomers, 600 Aβ monomers, and 20 tau monomers, respectively.

We also determined the octanol–water partition coefficient of compound **45** at pH 7.4 (LogD) and found a value of 2.588 ± 0.141 (mean ± SD on three independent experiments).

Given the pharmacological and physico-chemical properties of compound **45**, we decided to develop its [^18^F]-labeled analog for complementary in vivo investigations.

### 2.2. Preparation of **[^18^F]45**

**Scheme 4:**
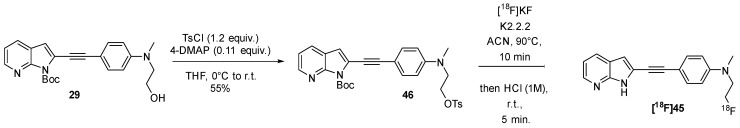
Synthesis of **[^18^F]45**.

The radiosynthesis of **[^18^F]45** was performed in a two-step procedure starting from the tosylated and protected precursor **46**, as the direct labeling of the unprotected pyrrole ring did not afford the desired radiotracer (Scheme 4) [33]. The first step consisted of aliphatic nucleophilic substitution at 90 °C for 10 min with the [18]KF complex. Then, the reaction was cooled, HCl (1 M) was added, and hydrolysis of the Boc group was conducted at r.t. for 5 min. HPLC purification was performed to obtain the pure desired compound which was formulated in EtOH/0.9% NaCl (*v*/*v*: 1/9). [^18^F]**45** was obtained in 85 ± 5 min with a radiochemical yield of 32 ± 3%. Molar activity, determined from a calibration with stable **45**, was around 130 GBq/µmole.

### 2.3. In Vivo Experiments

The next step in the characterization of our new PET tracer was to evaluate its ability to bind in vivo to α-syn fibrils. For this aim, we performed a simple preliminary imaging experiment in which the same α-syn fibrils as those used in in vitro experiments were surgically and unilaterally implanted in the brains of two adult Wistar rats. The accumulation of **[^18^F]45** after intravenous (i.v.) injection was compared, in each animal, between the implanted and intact non-implanted side. The fibrils were administered by stereotaxic injection into the right striatum, **[^18^F]45** was i.v. injected 1 day later, and image acquisition began at the time of injection using a microPET eXplore Vista (GE Healthcare, Buc, France)/CT system during 3 h. PET images were reconstructed and analyzed in three regions of interest, i.e., the left striatum without fibrils (C-ST), the right striatum with fibrils (I-ST), and the cerebellum (CE). At the end of imaging acquisition, each rat was sacrificed, and brain regions were removed, weighed, and their radioactivity measured.

**Figure 3:**
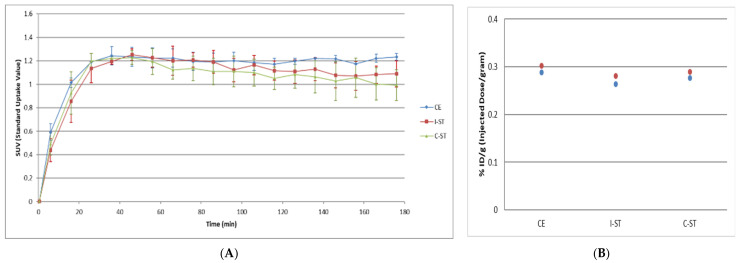
(**A**) Time-activity curves recorded during 3 h after i.v. injection of **[^18^F]45** (42–46 MBq) in the cerebellum (CE, blue), I-ST (right striatum with fibers, red), and C-ST (left striatum without fibers, green). Results are expressed as mean Standard Uptake Value (SUV) ± SEM, n = 2. (**B**) Radioactivity measured in each brain region (CE, I-ST, C-ST) of both rats sacrificed just after PET imaging. Results are expressed as percentage of injected dose per gram of tissue (%ID/g) for both rats (individual points).

As illustrated in Figure 3, the results of the dynamic PET imaging showed that **[^18^F]45** was able to cross the blood–brain barrier but had slow uptake and almost no washout. In addition, it homogeneously plateaued in the cerebellum as well as in the striatum receiving the fibrils (I-ST) and in the striatum without fibrils (C-ST), in agreement with the identical radioactivity accumulation found in the three brain regions at the end of imaging. This preliminary result thus indicated that the uptake of **[^18^F]45** was non-specific, as it was unable to bind in vivo to fibrils delivered within the brain.

## 3. Materials and Methods

### 3.1. Chemistry

^1^H NMR and ^13^C NMR spectra were recorded on a Bruker DPX 250 or 400 MHz instrument using CDCl_3_ and DMSO-*d*_6_. The chemical shifts are reported in parts per million (δ scale), and all coupling constant (*J*) values are reported in hertz. The following abbreviations were used for the multiplicities: s (singlet), br s (broad singlet), (doublet), t (triplet), q (quartet), p (pentuplet), m (multiplet), sext (sextuplet), and dd (doublet of doublets). Melting points are uncorrected. IR absorption spectra were obtained on a PerkinElmer PARAGON 1000 PC, and the values are reported in inverse centimeters. HRMS spectra were acquired in positive mode with an ESI source on a Q-TOF mass by the “Salsa” ICOA platform. Monitoring of the reactions was performed using silica gel TLC plates (silica Merck 60 F 254). Spots were visualized under UV light (254 nm and 356 nm). Column chromatography was performed using silica gel 60 (0.063–0.200 mm, Merck, Darmstadt, Germany). Microwave irradiation was carried out in sealed vessels placed in a Biotage Initiator or Biotage Initiator+ system (400 W maximum power). The temperatures were measured externally by IR. Pressure was measured by a non-invasive sensor integrated into the cavity lid. All reagents were purchased from commercial suppliers and were used without further purification.

***Sonogashira General procedure A:*** In a sealed tube and under argon, the 2-alkynylindole **1**, the desired iodobenzene (1.2 equiv.), CuI (1.0 mol %), and PdCl_2_(PPh_3_)_2_ (2.0 mol %) in Et_3_N (0.4 M) were successively introduced. The reaction mixture was heated at 60 °C for 3 h and after cooling at r.t., filtered using Celite^R^, and evaporated under reduced pressure.

***Sonogashira General procedure B*:** To a degassed (argon) solution of 2-iodoindoles **6**–**8**, the corresponding alkyne (1.2 equiv.), CuI (10.0 mol%) in a mixture of Et_3_N/THF (1:1) 0.1 M, was added the Pd(PPh_3_)_4_ (5.0 mol%). The reaction mixture was poured into a pre-heated oil bath at 60 °C and left for 4 h. After cooling at r.t., the reaction mixture was hydrolyzed with an aqueous solution of NH_4_Cl (10%). After extraction with EtOAc (3 times), the combined organic layers were dried over MgSO_4_, filtered and concentrated under reduced pressure.

***General procedure C for benzene sulfonyl removal:*** To a solution of *N*-benzenesulfonyl(aza)indolic compound (**2**–**5**, **9**–**14**, **21**, **22**, **25**, and **30**) in dioxane was added the sodium *tert*-butoxide (1.5 equiv.). The reaction mixture was heated at 80 °C for 18 h. After cooling to r.t. and concentration under reduced pressure, the crude material was dissolved in EtOAc and water was added. After extraction, the aqueous layer was washed with EtOAc (3 times), the combined organic layers were dried over MgSO_4,_ filtered and concentrated under reduced pressure.

***General procedure D for Boc protection*:** To a solution of 2-iodo(aza)indole **23** or **26** in THF (0.2 M) were added Boc_2_O (1.5 equiv.) and 4-DMAP (0.2 equiv.). The reaction mixture was stirred at r.t. for 12 h. After concentration under reduced pressure, purification was performed.

***General procedure E for Boc deprotection*:** To a solution of Boc protected derivative **25** or **28** in CH_2_Cl_2_ was added TFA (TFA/DCM ratio 1/2). After 1 h at r.t., a saturated aqueous solution of NaHCO_3_ was added. After extraction, the aqueous layer was extracted with CH_2_Cl_2_ (twice) and the combined organic layers were dried over MgSO_4_ and concentrated under reduced pressure.

### 3.2. In Vitro Experiments

#### 3.2.1. Expression and Purification of Recombinant α-Syn, Aβ1-42 and Tau

Wild-type α-syn and Aβ1-42 were expressed in the *Escherichia coli* strain BL21(DE3) (Stratagene, La Jolla, CA, USA) and purified as described [34,35]. The α-syn concentration was determined by measuring the absorbance at 280 nm using the extinction coefficient 5960 M^−1^ cm^−1^. For the Aβ1-42 peptide, the concentration was determined by a fluorometric method using the reactive compound fluorescamine (Sigma, San Jose, CA, USA) that reacts with primary amino groups to form a fluorescent product [36]. The human Tau protein (isoform h2N4RTau) was expressed in the pET11d vector in the *Escherichia coli* strain BL21(DE3) (Stratagene, La Jolla, CA, USA) and purified as described [37]. The concentration of purified Tau was determined by measuring the absorbance at 280 nm using the extinction coefficient 7575 M^−1^ cm^−1^.

#### 3.2.2. Production and Characterization of α-Syn, Aβ1-42, and Tau Fibrils

For assembly into fibrils, α-syn was incubated in 50 mM Tris-HCl, pH 7.5, 150 mM KCl at 37 °C under continuous shaking for 4 days. Lyophilized Aβ1-42 was dissolved in 1,1,1,3,3,3-hexafluoro-2-propanol (HFIP) at a concentration of 0.2 mg/mL and incubated for 24 h at 37 °C to achieve complete solubilization. HFIP was evaporated under a nitrogen stream to obtain a dry film. To start assembly into fibrils, the dry peptide was resuspended in PBS pH 7.4, 10% (*v*/*v*) DMSO and incubated at 37 °C without shaking for 3 days. The Tau protein was assembled in fibrils in a PBS buffer containing 1 mM of DTT in the presence of 1/12 heparin (*v*/*v*) at 37 °C under shaking. The assembly process was monitored using Thioflavin T (ThT) binding [38]. At regular time intervals, 10 µL protein aliquots were withdrawn and mixed with 400 µL of ThT (10 µM) in water. The ThT fluorescence was measured using a Cary Eclipse Spectrofluorometer (Varian Inc., Palo Alto, CA, USA) (Excitation wavelength set at 440 nm and emission wavelength set at 480 nm). Protein fibrils were systematically examined by transmission electron microscopy (TEM) in a Jeol 1400 transmission electron microscope following adsorption onto carbon-coated 200 mesh grids and negative staining with 1% uranyl acetate. The images were recorded with a Gatan Orius CCD camera (Gatan, Pleasanton, CA, USA). Finally, the percentage of α-syn or Aβ1-42 aggregated into fibrils was assessed by pelleting fibrils at 40,000× *g* for 20 min and by determining the concentration of soluble protein/peptide remaining in the supernatant.

#### 3.2.3. In Vitro Inhibition of Thioflavin T Binding and Ki Determination

Fresh solutions of each tested compound (2.5 mM in DMSO) were diluted in PBS pH 7.4 at concentrations ranging from 0.5 nM to 5 µM in the presence of α-syn, Aβ1-42 or Tau fibrils (200 nM, 500 nM, or 200 nM, respectively) and of 500 nM ThT in a final volume of 2 mL. The samples were incubated for 1 h at room temperature to achieve complete equilibration. The ThT binding to α-syn, Aβ1-42, or Tau fibrils was assessed by fluorescence measurements (excitation wavelength 440 nm, emission wavelength 480 nm). The ThT binding inhibition at increasing concentrations of each tested compound was measured as a reduction in ThT fluorescence. Due to the partial superposition between the fluorescence emission spectra of some compounds and ThT, the compounds were also incubated with α-syn, Aβ1-42 or Tau fibrils in the absence of ThT to assess their contribution to the measured fluorescence. For each concentration, the fluorescence values measured in the absence of ThT were subtracted from those measured in the presence of ThT. The data were reported as a fraction of the maximum value of ThT fluorescence measured in the absence of competitor compound (100% binding). For each curve, data were fitted to the dose–response 4-parameter logistic equation: Y = min + (max − min)/1 + (Y/IC_50_)^slope^ which allowed the IC_50_ value of each compound vs. ThT to be determined.

The corresponding inhibition constant Ki values were obtained using the Cheng and Prusoff equation [39]: Ki = IC_50/_(1 + [ThT]/Kd_ThT_) which takes into account the concentration of ThT used in the measurements (500 nM) and its affinities (Kd_ThT_) for α-syn, Aβ1-42, or Tau fibrils, which were 700 nM, 500 nM, and 350 nM, respectively. In the azaindole series, the IC_50_ could not be determined, as their emission spectra overlapped with that of ThT, making it impossible to distinguish their respective contributions to the measured fluorescence. In these cases, we used another method, direct fluorescence titration assays, that exhibited significant and measurable spectral changes upon binding to α-syn, Aβ1-42, or Tau fibrils to determine the affinity (Kd) and the density of binding sites within the fibrils (Bmax).

#### 3.2.4. In Vitro Fluorescence Titration Assays: Kd and Bmax Determination

Fresh solutions of each tested compound (2.5 mM in DMSO) were diluted in PBS pH 7.4 at concentrations ranging from 0.5 nM to 5 µM in the presence or absence of α-syn, Aβ1-42, or Tau fibrils (200 nM, 500 nM, or 200 nM, respectively) in a final volume of 2 mL. After 1 h incubation at room temperature, the binding of each compound to α-syn, Aβ1-42, or Tau fibrils was assessed by fluorescence measurements using the appropriate excitation/emission wavelengths (i.e., those yielding the highest fluorescence ratio between fibril-bound and free compound). For any given concentration of compound added to the fibrils, the concentrations of compound bound to the fibrils and free in solution were determined on the basis of the measured fluorescence. Since at each point of the titration: F_m_ = F_B_ × B + F_F_ × (T − B) where Fm is the measured fluorescence of the compound in the presence of fibrils at a given compound concentration T, F_B_ is the specific molar fluorescence of the bound compound, F_F_ is the specific molar fluorescence of the free compound, and B is the concentration of compound bound to the fibrils at any point of the titration. F_F_ was determined by measuring the fluorescence of known compound concentrations in the absence of fibrils. F_B_ was determined by measuring the maximum fluorescence of known compound concentrations upon saturation with α-syn, Aβ1-42, or Tau fibrils.

Therefore, at each point of the titration, the concentration of bound compound B could be determined and was B = (F_m_ − F_F_ × T)/F_B_ − F_F_. The B values thus determined versus the respective concentrations of free compound were analyzed by fitting to the Michaelis-Menten equation. The affinity values (Kd) and the densities of binding sites within α-syn, Aβ1-42 or Tau fibrils (Bmax) were determined by Scatchard analysis, which also revealed the presence of multiple classes of binding sites.

### 3.3. Preparation of the Precursor and [^18^F]45

***tert*-Butyl 2-((4-(methyl(2-(tosyloxy)ethyl)amino)phényl)éthynyl)-1*H*-pyrrolo[2,3-*b*]pyridine-1-carboxylate 46**

To a solution of TsCl (0.12 g, 0.63 mmol, 1.2 equiv.), Et_3_N (0.15 mL, 1.05 mmol, 2 equiv.) and DMAP (0.013 g, 0.11 mmol, 0,2 equiv.) in THF (11 mL) at 0 °C, a solution of **29** (0.21 g, 0.53 mmol, 1 equiv.) in THF (6 mL) was added dropwise. The solution was stirred at r.t. for 16 h, then was concentrated under reduced pressure. The crude material was purified by flash chromatography on silica gel (EtOAc/PE 30/70) to afford **46** as a yellow solid (25 mg, 55%). Mp 151–153 °C. *R*_f_ = 0.31 (EtOAc/PE 20/80). IR (ν, cm^−1^, neat) 2986, 2360, 2198, 1749, 1605, 1544, 1514, 1407, 1348, 1306, 1253, 1187, 1170, 1114, 1092, 1010, 972, 900, 804, 776, 663, 556, 508. RMN ^1^H (250 MHz, CDCl_3_) δ 8.48 (dd, *J* = 4.8, 1.7 Hz, 1H), 7.78 (dd, *J* = 7.8, 1.7 Hz, 1H), 7.73–7.52 (m, 2H), 7.39–7.26 (m, 2H), 7.30–7.17 (m, 2H), 7.14 (dd, *J* = 7.8, 4.8 Hz, 1H), 6.78 (s, 1H), 6.56–6.42 (m, 2H), 4.16 (t, *J* = 5.8 Hz, 2H), 3.62 (t, *J* = 5.8 Hz, 2H), 2.90 (s, 3H), 2.39 (s, 3H), 1.66 (s, 9H). RMN ^13^C (101 MHz, CDCl_3_,) δ 148.57 (Cq), 148.34 (Cq), 148.06 (Cq), 145.77 (CH), 145.00 (Cq), 132.83 (2×CH), 132.64 (Cq), 129.82 (2×CH), 128.33 (CH), 127.81 (2×CH), 121.86 (Cq), 121.53 (Cq), 118.85 (CH), 111.75 (CH), 111.64 (2×CH), 109.99 (Cq), 96.66 (Cq), 84.58 (Cq), 79.94 (Cq), 66.69 (CH_2_), 50.97 (CH_2_), 38.96 (CH_3_), 28.22 (3×CH_3_), 21.65 (CH_3_). HRMS (ESI^+^) calcd for C_30_H_32_N_3_O_5_S (M + H^+^): 546.2057, found: 546.2053.

Radiosynthesis was carried out in two steps: radiofluorination of the precursor **46** followed by hydrolysis of the BOC protecting group to afford [^18^F]**45**. Radiofluorination was performed using an FXFNPro synthesizer (GE Healthcare, Buc, France). [^18^F] ions were produced by the nuclear reaction of ^18^O(p,n)^18^F using a PETtrace cyclotron (GE Healthcare) and transferred under helium pressure to the synthesizer. The radioactivity was trapped on an anionic exchange cartridge QMA (Waters, Milford, MA, USA) which was activated by 10 mL of K_2_CO_3_ (0.5 M) rinsed by 10 mL of water. The radioactivity was released from the cartridge to the reactor by a solution containing 7.2 mg of K_2.2.2_ dissolved in 715 µL of ACN and 3.8 mg of K_2_CO_3_ dissolved in 285 µL of water. Azeotropic distillation was performed twice using each time 1 mL of acetonitrile at 100 °C under He flow and vacuum. To the anhydrous [^18^F]KF solution, precursor **26** (2 mg in 700 µL of ACN) was added and then heated to 90 °C for 10 min. Then, the mixture was cooled to RT, and 500 µL of HCl (1 M) were added, and the reaction was stirred at RT for 5 min. The reaction was stopped by adding 8 mL of water. The crude solution was passed through a *t*-C18 Sep-Pak Plus cartridge (Waters). The reactor and cartridge were rinsed with water (5 mL), and the crude compound was eluted from the cartridge with acetonitrile (2 mL) and diluted with 2 mL of ammonium acetate (0.1 M). The 4 mL of solution were loaded in an HPLC loop and purification occurred on a ZORBAX Eclipse XDB-C18 (9.4 × 250 mm, 5 μm, Agilent, Santa Clara, CA, USA) at a 5 mL/min flow with acetonitrile/ammonium acetate, 0.1 M: 50/50 as mobile phase. The peak was collected with a time retention of 23 min, diluted in water (25 mL), the solution was trapped on a *t*-C18 light cartridge (Waters) and the cartridge was rinsed with water (5 mL). The product was eluted by ethanol (1 mL) and the formulation was completed by 0.9%NaCl (9 mL). [^18^F]45 was obtained in 85 ± 5 min with a radiochemical yield of 32 ± 3% and molar activity around 130 GBq/µmole. Data for the determination of the molar activity are given in “Supplementary Materials”.

### 3.4. In Vivo Experiments

PET imaging experiments were performed with **[^18^F]45** on two adult male Wistar rats weighing 250–300 g (Janvier, Le Genest-Saint-Isle, France). The animals were housed in a temperature (23 °C) and humidity (43%) controlled environment under a 12-h light-dark cycle with food and water available ad libitum, and experimental procedures were conducted in accordance with the requirements of the European Community Council Directive 2010/63/EU for the care of laboratory animals and with the authorization of the Regional Ethical Committee (Authorization N°2012-03-1).

The rats were scanned one hour after receiving an intra-cerebral administration of α-syn. They were anesthetized with isoflurane (4% in O_2_, 500 mL/min, Baxter, France), placed on a stereotaxic frame (Stoelting, Phymep, Paris, France) and maintained under isoflurane 2.5% (500 mL/min) during surgery. Body temperature was monitored and kept at 37 °C. Fibrils of α-syn were prepared as described above (solution of 10 mg/mL in PBS). This solution was administered in two sites of the right striatum of each rat using a 25 μL Hamilton Gastight syringe (Hamilton, Massy, France) with a 30-gauge needle (Phymep, Paris, France) connected to a perfusion system. A volume of 5 μL corresponding to 50 µg of fibrils was infused in each site at the following coordinates from bregma: AP + 0.5/ML-2.5/DV-5, and AP-0.5/ML-4/DV-5 [40]. The animals received buprenorphine (0.05 mg/kg subcutaneously) following surgery for post-operative pain.

For the PET experiment, each rat was anesthetized with isoflurane (Baxter, Guyancourt, France), at 4–5% in O_2_ for induction and then 1.5–2% during scanning, and received an i.v. bolus injection of 42–46 MBq of **[^18^F]45** (molar activity 99.69 GBq/µmol) into the tail vein. Before PET acquisition, a 5-min computed tomography (CT) scan was acquired for attenuation correction. The acquisition was made during 181 min with a microPET eXplore Vista/CT system (GE Healthcare, France) which has an effective axial/trans axial field of view (FOV) of 4.8/6.7 cm, a spatial resolution of 1–2 mm and a sensitivity above 2.5% in the whole FOV. The PET list-mode scans were rebinned into 19 frames: 1 frame of 60 s (before injection of the tracer), followed by 18 of 600 s. The quantification of the tracer uptake was extracted from the last 6 120-s frames corresponding to 49–61 min of acquisition after tracer injection. Each image was corrected for randoms, scatter, and attenuation, and was reconstructed using a 2-dimensional OSEM algorithm (GE Healthcare, France) into voxels of 0.3875 × 0.3875 × 0.775 mm^3^. PET images were reconstructed and analyzed using PMOD (3.403, PMOD Technologies, Zurich, Switzerland). The regions of interest (ROIs) were the left non injected striatum (contralateral-striatum, C-ST) and right injected striatum (ipsilateral-striatum, I-ST), and cerebellum (CE).

After imaging, animals were sacrificed, their brains were removed, and the right and left striatum and cerebellum were dissected, weighed, and their radioactivity measured using a γ-counter (2480 Gamma counter Wizard, Perkin Elmer, counting efficiency of 48% for fluorine-18), and the percent injected dose per gram of tissue (%ID/g) was calculated by comparison with samples to standard dilutions of the injected solution. Using the cerebellum (CE) as reference region, the ratio of %ID/g in each region to that obtained for the CE ([%ID/g]r) was used as quantitative criterion.

## 4. Conclusions

We have developed original alkynyl(aza)indole ligands targeting alphasynuclein using Sonogashira cross-coupling chemistry to obtain the alkynyl function. Fifteen final ligands were synthesized bearing indoles or azaindoles from one side of the alkyne and a substituted phenyl ring on the opposite side of the alkyne. The final ligands were tested to determine Ki and/or Kd toward α-syn, tau, and Aβ. The SAR showed that the indole series exhibited moderate to low affinity for α-syn and, moreover, lower Ki toward Aβ and tau, highlighting the low potency of these series to afford an α-syn tracer. The introduction of a nitrogen on the different positions of the phenyl to obtain the corresponding azaindole (**41**–**44**) resulted for most of the compounds in better affinity for α-syn compared to the indole analog **38**. Among the four different positions tested, the 7th proved to be superior to the others in terms of affinity for α-syn and selectivity towards Aβ. For most of the azaindole compounds, as for the indole series, no selectivity towards tau was observed. A fluorinated derivative of **43** was prepared with a view to obtaining a ^18^F tracer. The resulting compound **45** exhibited the highest affinity for α-syn but without selectivity against tau and Aβ. Nevertheless, **45** was efficiently labeled with ^18^F and preclinically evaluated, but non-specific uptake was observed, confirming the in vitro results. Thus, further pharmacomodulations on the azaindole series are required to improve at least the selectivity towards tau in order to attain the objective of developing a selective ^18^F α-syn radiopharmaceutical [41].

## Figures and Tables

**Table 1:** In vitro affinity of the new compounds for α-syn, Aβ, and Tau fibrils.

N°	Compound	Ki (nM) ^a^			Kd (nM) ^b^		
		α-Syn	Aβ	Tau	α-Syn	Aβ	Tau
**31**	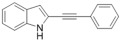	>100	68.9 ± 30	19.6 ± 2	ND	ND	ND
**32**	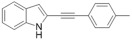	>100	37.9 ± 13	13.1 ± 2	ND	ND	ND
**33**	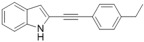	35.9 ± 5	3.3 ± 1	32.9 ± 8	ND	ND	ND
**34**	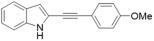	96.7 ± 14	68.9 ± 14	33.1 ± 7	ND	ND	ND
**35**	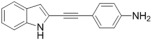	40.4 ± 6	26.2 ± 11	123 ± 28	ND	ND	ND
**36**	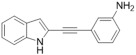	>100	15.3 ± 7	7.2 ± 1	ND	ND	ND
**37**	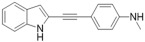	47.8 ± 9	6.6 ± 2	17.2 ± 3	ND	ND	ND
**38**	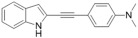	36 ± 7	64.4 ± 40	27.6 ± 3	ND	ND	ND
**39**	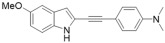	21.7 ± 6	2.2 ± 1	12.7 ± 1	ND	ND	ND
**40**	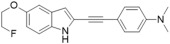	>100	10.1 ± 5	15.8 ± 3	ND	ND	ND
**41**	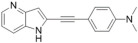	26 ± 16	21.3 ± 7	12.0 ± 1	3.25 ± 0.2	2.9 ± 2	0.13 ± 0.05
**42**	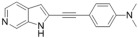	28.7 ± 8	91.5 ± 11	8.4 ± 1	1.4 ± 1	5.2 ± 3	0.49 ± 0.2
**43**	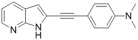	4.7 ± 2	24.4 ± 9	4.61 ± 0.2	0.53 ± 0.2	11.8 ± 3	0.44 ± 0.1
**44**	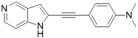	26.0 ± 16	21.3 ± 7	12.1 ± 1	1.0 ± 0.5	3.6 ± 2	2.4 ± 0.9
**45**	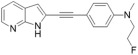	ND	ND	ND	2.4 ± 1	1.2 ± 1	0.97 ± 0.4

^a^ The Ki were calculated from IC_50_ values determined by competition experiments with thioflavin T (ThT). ^b^ The Kd by direct assay on α-syn, Aβ or tau fibrils. The results are expressed as mean ± SD of 3 independent experiments. ND, non determined.

## Data Availability

Data is contained within the article or Supplementary Materials.

## Supplementary Materials

The following supporting information can be downloaded at: https://www.mdpi.com/article/10.3390/ph18111638/s1: Methods for synthesis and characterization (^1^H and ^13^C NMR spectra) of products **2**–**45**.
