# Supplementary material for: Design, Synthesis and Evaluation of the First 2-Alkynyl(aza)indole 18F Probe Targeting α-Synuclein Aggregates
Source: Pharmaceuticals (Basel). 2025 Oct 29;18(11):1638. doi: 10.3390/ph18111638 (PMC12655733; doi:10.3390/ph18111638)

Design, Synthesis and Evaluation of the First 2-Alkynyl(aza)indole ^{18}F Probe Targeting α-Synuclein Aggregates

Liliana Boiaryna [1], Laura Pieri [2,3,†], Sylvie Chalon [2], Sophie Serrière [2], Sylvie Bodard [2], Gabrielle Chicheri [2], Elisa Chenaf [1], Franck Suzenet [1], Ronald Melki [3,‡], Frédéric Buron [1,*], Sylvain Routier [1,*], Johnny Vercouillie [2]

[1] Université d'Orléans, CNRS, ICOA, UMR 7311, 45067 Orléans, France

[2] UMR 1253, iBrain, Université de Tours, Inserm, 37044 Tours, France

[3] Paris-Saclay Institute of Neuroscience, CNRS UMR 9197, Université Paris-Saclay, 91190 Gif-sur-Yvette, France

* Correspondence: frederic.buron@univ-orleans.fr (F.B.); sylvain.routier@univ-orleans.fr (S.R.)

[†] Present addresses: Institute for Integrative Biology of the Cell (I2BC), CEA, CNRS, Université Par-is-Saclay, 91190 Gif-sur-Yvette, France.

[‡] Present addresses: Laboratory of Neurodegenerative Diseases, CEA, CNRS, Institute François Jacob (MIRCen), 92260 Fontenay-Aux-Roses, France.

Synthesis of compounds 2–45……………………………………………………...2–11

Spectra of ^{1}H, ^{13}C for products 2–45………………………………………………12–47

Determination of molar activity: Method and HPLC data…………………………..48

Molar activity data and the microSPECT images ……………………………………49

2-(Phenylethynyl)-1-(phenylsulfonyl)-1*H*-indole 2 [25]

The compound was prepared from 2-ethynyl-1-(phenylsulfonyl)-1*H*-indole **1** after flash chromatography on silica gel (EtOAc/Petroleum ether 2/98) to afford **2** as a white solid (91%). Mp 98-100°C. R_f = 0.53 (EtOAc/Petroleum ether 10/90). ^{1}H RMN (250 MHz, $CDCl_3$) δ 8.28 (dd, *J* = 8.4, 0.8 Hz, 1H), 8.04-7.96 (m, 2H), 7.71-7.61 (m, 2H), 7.57-7.46 (m, 2H), 7.46-7.35 (m, 6H), 7.33-7.22 (m, 1H), 6.95 (d, *J* = 0.7 Hz, 1H). ^{13}C RMN (100 MHz, $CDCl_3$,) δ 138.8 (Cq), 136.7 (Cq), 134.0 (CH), 131.6 (2×CH), 129.2 (2×CH), 129.2 (Cq), 129.1 (Cq), 128.7 (2×CH), 127.1 (2×CH), 126.1 (CH), 124.1 (CH), 122.6 (Cq), 121.2 (CH), 121.0 (Cq), 117.1 (CH), 114.8 (CH), 97.0 (Cq), 80.7 (Cq).

1-(Phenylsulfonyl)-2-(*p*-tolylethynyl)-1*H*-indole 3.

The compound was prepared from 2-ethynyl-1-(phenylsulfonyl)-1*H*-indole **1** after flash chromatography on silica gel (EtOAc/Petroleum ether 2/98) to afford **3** as a white solid (75%). Mp 121-123°C. R_f = 0.48 (EtOAc/Petroleum ether 10/90). ^{1}H RMN (250 MHz, $CDCl_3$) δ 8.28 (d, *J* = 8.4 Hz, 1H), 8.05-7.96 (m, 2H), 7.55 (d, *J* = 8.1 Hz, 2H), 7.53-7.45 (m, 2H), 7.44-7.35 (m, 3H), 7.31-7.18 (m, 3H), 6.92 (s, 1H), 2.41 (s, 3H). ^{13}C RMN (101 MHz, $CDCl_3$) δ 139.3 (Cq), 138.7 (Cq), 136.6 (Cq), 133.9 (CH), 131.5 (2×CH), 129.3 (2×CH), 129.1 (2×CH, 1Cq), 127.0 (2×CH), 125.9 (CH), 124.0 (CH), 121.2 (Cq), 121.1 (CH), 119.4 (Cq), 116.7 (CH), 114.7 (CH), 97.2 (Cq), 80.0 (Cq), 21.7 (CH_3).

2-((4-Ethylphenyl)ethynyl)-1-(phenylsulfonyl)-1*H*-indole 4.

The compound was prepared from 2-ethynyl-1-(phenylsulfonyl)-1*H*-indole **1** after flash chromatography on silica gel (EtOAc/Petroleum ether 5/95) to afford **4** as a pale yellow oil (99%). R_f = 0.29 (EtOAc/Petroleum ether 10/90). IR (ν, cm^{-1}, neat) 2964, 2930, 2211, 1682, 1581, 1412, 1375, 1308, 1269, 1175, 1048, 925, 832, 745. ^{1}H RMN (250 MHz, $CDCl_3$) δ 8.29 (d, *J* = 8.4 Hz, 1H), 8.06-7.96 (m, 2H), 7.59 (d, *J* = 8.1 Hz, 2H), 7.56-7.47 (m, 2H), 7.48-7.37 (m, 3H), 7.31-7.21 (m, 3H), 6.94 (s, 1H), 2.73 (q, *J* = 7.6 Hz, 2H), 1.30 (t, *J* = 7.6 Hz, 3H). ^{13}C RMN (63 MHz, $CDCl_3$) δ 145.6 (Cq), 138.7 (Cq), 136.6 (Cq), 133.8 (CH), 131.6 (2×CH), 129.1 (2×CH), 128.5 (Cq), 128.1 (2×CH), 127.0 (2×CH), 125.9 (CH), 123.9 (CH), 121.2 (Cq), 121.0 (CH), 119.7 (Cq), 116.6 (CH), 114.7 (CH), 97.2 (Cq), 79.9 (Cq), 29.0 (CH_2), 15.3 (CH_3). HRMS (ESI$^+$) calcd for $C_{24}H_{20}NO_2S$ (M+H$^+$) : 386.1209, found: 386.1211.

2-((4-Methoxyphenyl)ethynyl)-1-(phenylsulfonyl)-1*H*-indole 5.

The compound was prepared from 2-ethynyl-1-(phenylsulfonyl)-1*H*-indole **1** after flash chromatography on silica gel (EtOAc/Petroleum ether 5/95) to afford **5** as a white solid (76%). Mp 94-96°C. R_f = 0.24 (EtOAc/Petroleum ether 10/90). ^{1}H RMN (400 MHz, $CDCl_3$) δ 8.30 (d, *J* = 8.5 Hz, 1H), 8.05-7.98 (m, 2H), 7.65-7.58 (m, 2H), 7.57-7.47 (m, 2H), 7.46-7.37 (m, 3H), 7.29 (t, *J* = 7.5 Hz, 1H), 7.00-6.94 (m, 2H), 6.92 (s, 1H), 3.88 (s, 3H). ^{13}C RMN (100 MHz, $CDCl_3$) δ 160.3 (Cq), 138.8 (Cq), 136.6 (Cq), 133.9 (CH), 133.2 (2×CH), 129.2 (Cq), 129.2 (2×CH), 128.6 (Cq), 127.1 (2×CH), 125.9 (CH), 124.0 (CH), 121.4 (Cq), 121.1 (CH), 116.4 (CH), 114.7 (CH), 114.6 (Cq), 114.3 (CH), 97.2 (Cq), 79.5 (Cq), 55.5 (CH_3).

4-((1-Phenylsulfonyl)-1*H*-indol-2-yl)ethynyl)aniline 9.

The compound was prepared from 2-iodo-1-(phenylsulfonyl)-1*H*-indole **6** using the general procedure B to afford **9** after flash chromatography on silica gel (EtOAc/Petroleum ether 10/90) as a white solid (89%). Mp 127-129°C, R_f = 0.28 (EtOAc/Petroleum ether 30/70). IR (ν, cm^{-1}, neat) 3471, 3378, 3072, 2205, 1622, 1605, 1508, 1448, 1360, 1268, 1148, 1088, 1032, 999, 816, 753, 724, 627. ^{1}H RMN (400 MHz, $CDCl_3$) δ 8.29 (d, *J* = 8.5 Hz, 1H), 8.07-7.91 (m, 2H), 7.60-7.33 (m, 7H), 7.25 (m, 1H), 6.88 (s, 1H), 6.77-6.51 (m, 2H), 3.96 (large s, 2H). ^{13}C RMN (101 MHz, $CDCl_3$) δ 147.5 (Cq), 138.7 (Cq), 136.5 (Cq), 133.8 (CH), 133.0 (2×CH), 129.3 (Cq), 129.1 (2×CH), 127.0 (2×CH), 125.6 (CH), 123.9 (CH), 121.7 (Cq), 120.9 (CH), 115.8 (CH), 114.8 (2×CH), 114.6 (CH), 111.5 (Cq), 98.1 (Cq), 78.6 (Cq). HRMS (ESI$^+$) calcd for $C_{22}H_{17}N_2O_2S$ (M+H$^+$) : 373.1005, found: 373.1007.

3-((1-(Phenylsulfonyl)-1*H*-indol-2-yl)ethynyl)aniline 10.

The compound was prepared from 2-iodo-1-(phenylsulfonyl)-1*H*-indole **6** using the general procedure B to afford **10** after flash chromatography on silica gel (EtOAc/Petroleum ether 20/80) as an orange solid (87%). Mp 107-109°C. R_f = 0.21 (EtOAc/Petroleum ether 20/80). IR (ν, cm^{-1}, neat) 3445, 3368, 3069, 2212, 1625, 1599, 1471, 1367, 1330, 1284, 1185, 1042, 955, 860, 815, 725, 684, 603. ^{1}H RMN (400 MHz, $CDCl_3$) δ 8.30 (dd, *J* = 8.5, 1.1 Hz, 2H), 8.09-7.88 (m, 2H), 7.55-7.46 (m, 2H), 7.47-7.37 (m, 2H), 7.30 (m, 1H), 7.21 (t, *J* = 7.7 Hz, 1H), 7.07 (dt, *J* = 7.6, 1.1 Hz, 1H), 7.00 (t, *J* = 2.0 Hz, 1H), 6.94 (s, 1H), 6.75 (ddd, *J* = 8.2, 2.5, 1.0 Hz, 1H), 3.68 (large s, 2H). ^{13}C RMN (101 MHz, $CDCl_3$) δ 146.6 (Cq), 138.6 (Cq), 136.6 (Cq), 133.9 (CH), 129.5 (CH), 129.2

(2×CH), 129.1 (Cq), 127.0 (2×CH), 126.0 (CH), 124.0 (CH), 123.1 (Cq), 121.8 (CH), 121.1 (CH), 121.1 (Cq), 117.6 (CH), 116.9 (CH), 116.1 (CH), 114.7 (CH), 97.3 (Cq), 79.9 (Cq). HRMS (ESI$^+$) calcd for$C_{22}H_{17}N_2O_2S$ (M+H$^+$) : 373.1005, found: 373.1007.

***N*-Methyl-4-((1-(phenylsulfonyl)-1*H*-indol-2-yl)ethynyl)aniline 11.**

The compound was prepared from 2-iodo-1-(phenylsulfonyl)-1*H*-indole **6** using the general procedure B to afford **11** after flash chromatography on silica gel (EtOAc/Petroleum ether 10/90) as a pale yellow oil (88%). R_f = 0.25 (EtOAc/Petroleum ether 20/80). IR (ν, cm^{-1}, neat) 3424, 2887, 2820, 2203, 1606, 1581, 1553, 1446, 1371, 1267, 1150, 1047, 821, 724, 629. ^{1}H RMN (400 MHz, $CDCl_3$) δ 8.30 (dq, *J* = 8.4, 1.0 Hz, 1H), 8.07-7.95 (m, 2H), 7.58-7.34 (m, 7H), 7.27 (m, 1H), 6.87 (m, 1H), 6.67-6.55 (m, 2H), 4.07 (large s, 1H), 2.91 (s, 3H). ^{13}C RMN (101 MHz, $CDCl_3$) δ 149.9 (Cq), 138.8 (CH), 136.5 (Cq), 133.8 (Cq), 133.0 (2×CH), 129.3 (Cq), 129.1 (2×CH), 127.1 (2×CH), 125.5 (CH), 123.9 (CH), 121.9 (Cq), 120.9 (CH), 115.5 (CH), 114.6 (CH), 112.1 (2×CH), 109.8 (Cq), 98.6 (Cq), 78.5 (Cq), 30.3 (CH_3). HRMS (ESI$^+$) calcd for $C_{23}H_{19}N_2O_2S$ (M+H$^+$) : 387.1162, found: 387.1163.

***N,N*-Dimethyl-4-((1-(phenylsulfonyl)-1*H*-indol-2-yl)ethynyl)aniline 12.**

The compound was prepared from 2-iodo-1-(phenylsulfonyl)-1*H*-indole **6** using the general procedure B to afford **12** after flash chromatography on silica gel (EtOAc/Petroleum ether 5/95) as a white solid (83%). Mp 162-164°C. R_f = 0.44 (EtOAc/Petroleum ether 15/85). IR (ν, cm^{-1}, neat) (ν, cm^{-1}, neat) 2886, 2207, 1888, 1605, 1551, 1516, 1479, 1447, 1378, 1360, 1305, 1271, 1230, 1207, 1188, 1177, 1151, 1126, 1047, 1020, 999, 945, 923, 866, 785, 752, 726, 681, 649, 629, 614. ^{1}H RMN (400 MHz, $CDCl_3$) δ 8.29 (d, *J* = 8.5 Hz, 1H), 8.06-7.99 (m, 2H), 7.57-7.46 (m, 4H), 7.44-7.36 (m, 3H), 7.30-7.24 (m, 1H), 6.86 (s, 1H), 6.73 (d, *J* = 8.9 Hz, 2H), 3.05 (s, 6H). ^{13}C RMN (100 MHz, $CDCl_3$) δ 150.7 (Cq), 138.9 (Cq), 136.6 (Cq), 133.9 (CH), 132.9 (2×CH), 129.4 (Cq), 129.2 (2×CH), 127.2 (2×CH), 125.5 (CH), 123.9 (CH), 122.1 (Cq), 120.9 (CH), 115.5 (CH), 114.7 (CH), 111.9 (2×CH), 109.1 (Cq), 98.7 (Cq), 78.8 (Cq), 40.3 (2×CH_3). HRMS (ESI$^+$) calcd for $C_{24}H_{21}N_2O_2S$ (M+H$^+$) : 401.1318, found: 401.1321.

4-((5-Methoxy-1-(phenylsulfonyl)-1*H*-indol-2-yl)ethynyl)-*N,N*-dimethylaniline 13.

The compound was prepared from 2-iodo-1-(phenylsulfonyl)-1*H*-indole **6** using the general procedure B to afford **13** after flash chromatography on silica gel (EtOAc/Petroleum ether

20/80) as a white solid (92%). Mp 141-143°C. R_f = 0.37 (EtOAc/Petroleum ether 20/80). IR (ν, cm^{-1}, neat) 2895, 2204, 1605, 1582, 1446, 1369, 1217, 1190, 1090, , 911, 852, 761, 706, 685. ^{1}H NMR (400 MHz, $CDCl_3$) δ 8.16 (d, J = 9.1 Hz, 1H), 7.98 (m, 2H), 7.58-7.48 (m, 3H), 7.41 (m, 2H), 7.00 (dd, J = 9.1, 2.5 Hz, 1H), 6.91 (d, J = 2.5 Hz, 1H), 6.78 (s, 1H), 6.75-6.68 (m, 2H), 3.84 (s, 3H), 3.05 (s, 6H). ^{13}C NMR (101 MHz, $CDCl_3$) δ 156.7 (Cq), 150.5 (Cq), 138.7 (Cq), 133.7 (CH), 132.8 (2×CH), 131.1 (Cq), 130.4 (Cq), 129.0 (2×CH), 127.0 (2×CH), 122.6 (Cq), 115.6 (CH), 115.4 (CH), 114.6 (CH), 111.8 (2×CH), 109.0 (Cq), 102.7 (CH), 98.6 (Cq), 78.8 (Cq), 55.6 (CH_3), 40.2 (2×CH_3). HRMS (ESI^+) calcd for $C_{25}H_{22}N_2O_3S$ ($M+H^+$) : 431.1424, found: 431.1424.

***N,N*-Dimethyl-4-((1-(phenylsulfonyl)-1*H*-pyrrolo[2,3-*c*]pyridin-2-yl)ethynyl)aniline 21.**

The compound was prepared from 2-iodo-1-(phenylsulfonyl)-1*H*-pyrrolo[2,3-*c*]pyridine **17** using the general procedure B to afford **21** after flash chromatography on silica gel (EtOAc/Petroleum ether 30/70) as a pale brown solid (80%). Mp 168-170°C. R_f = 0.34 (EtOAc/Petroleum ether 30/70). IR (ν, cm^{-1}, neat) 3058, 2205, 1679, 1536, 1446, 1351, 1272, 1123, 1088, 836, 729, 680, 574, 512. ^{1}H RMN (250 MHz, $CDCl_3$) δ 9.55 (s, 1H), 8.43 (d, J = 5.1 Hz, 1H), 8.12-7.96 (m, 2H), 7.64-7.50 (m, 3H), 7.47-7.37 (m, 3H), 6.79 (s, 1H), 6.72 (d, J = 8.9 Hz, 2H), 3.05 (s, 6H). ^{13}C RMN (63 MHz, $CDCl_3$) δ 150.9 (Cq), 142.7 (CH), 142.9 (Cq), 138.3 (Cq), 136.7 (CH), 134.8 (Cq), 134.2 (CH), 133.1 (2×CH), 129.3 (2×CH), 127.3 (2×CH), 125.2 (Cq), 114.9 (CH), 113.2 (CH), 111.7 (2×CH), 107.9 (Cq), 101.0 (Cq), 78.0 (Cq), 40.1 (2×CH_3). HRMS (ESI^+) calcd for $C_{23}H_{20}N_3O_2S$ ($M+H^+$) : 402.1271, found : 402.1273.

***N,N*-Dimethyl-4-((1-(phenylsulfonyl)-1*H*-pyrrolo[2,3-*b*]pyridin-2-yl)ethynyl)aniline 22.**

The compound was prepared from 2-iodo-1-(phenylsulfonyl)-1*H*-pyrrolo[2,3-*c*]pyridine **17** using the general procedure B to afford **22** after flash chromatography on silica gel (EtOAc/Petroleum ether 10/90) as a pale yellow solid (62%). Mp 185-187°C. R_f = 0.18 (EtOAc/Petroleum ether 10/90). IR (ν, cm^{-1}, neat) 2918, 2194, 1732, 1605, 1578, 1447, 1398, 1226, 1183, 1089, 997, 811, 726, 626. ^{1}H RMN (250 MHz, $CDCl_3$) δ 8.47 (dd, J = 4.8, 1.6 Hz, 1H), 8.28-8.17 (m, 2H), 7.73 (dd, J = 7.9, 1.6 Hz, 1H), 7.57-7.48 (m, 3H), 7.47-7.37 (m, 2H), 7.16 (dd, J = 7.9, 4.8 Hz, 1H), 6.75 (s, 1H), 6.73-6.63 (m, 2H), 3.02 (s, 6H). ^{13}C RMN (63 MHz, $CDCl_3$) δ 150.7 (Cq), 148.3 (CH), 145.3 (Cq), 139.2 (Cq), 133.8 (CH), 132.9 (2×CH), 129.0 (2×CH), 128.6 (CH), 127.8

(2×CH), 122.6 (Cq), 121.8 (Cq), 119.4 (CH), 111.8 (2×CH), 111.5 (CH), 108.7 (Cq), 99.7 (Cq), 78.6 (Cq), 40.15 (2×CH_3). HRMS (ESI$^+$) calcd for $C_{23}H_{20}N_3O_2S$ (M+H$^+$) : 402.1271, trouve: 402.1274.

2-Iodo-1*H*-pyrrolo[3,2-*c*]pyridine 23 [32]

The compound was prepared from 2-iodo-1-(phenylsulfonyl)-1*H*-pyrrolo[3,2-*c*]pyridine **16** using the general procedure C to afford **23** after precipitation in Et2O as a white solid (65%) which was readily used. Mp 247-249°C. R_f = 0.19 (EtOAc/Petroleum ether 30/70). IR (ν, cm^{-1}, neat) 3349, 3120, 3080, 2930, 2770, 2644, 1668, 1574, 1423, 1324, 1292, 1168, 1028, 948, 808, 796, 629, 511. ^{1}H RMN (400 MHz, DMSO-d$_6$) δ 12.08 (s, 1H), 8.73 (s, 1H), 8.09 (d, *J* = 5.6 Hz, 1H), 7.29 (d, *J* = 5.6 Hz, 1H), 6.81 (s, 1H). ^{13}C RMN (101 MHz, DMSO-*d*$_6$) δ ^{13}C NMR (101 MHz, DMSO) δ 142.3 (Cq), 141.6 (CH), 140.6 (CH), 127.0 (Cq), 110.3 (CH), 106.3 (CH), 81.5 (Cq). HRMS (ESI$^+$) calcd for $C_7H_6IN_2$ (M+H$^+$) : 244.9570, found : 244.9568.

***tert*-Butyl 2-iodo-1*H*-pyrrolo[3,2-*c*]pyridine-1-carboxylate 24**

The compound was prepared from **23** using the general procedure D to afford **24** after flash chromatography on silica gel (EtOAc/Petroleum ether 20/80) as a white solid (88%). Mp 82-84°C. R_f = 0.19 (EtOAc/Petroleum ether 20/80). IR (ν, cm^{-1}, neat) 3041, 2983, 1733, 1592, 479, 1394, 1271, 1178, 1056, 914, 816, 768, 644, 544, 505. ^{1}H RMN (250 MHz, $CDCl_3$) δ 8.66 (d, *J* = 1.0 Hz, 1H), 8.27 (dd, *J* = 5.9, 1.0 Hz, 1H), 7.81 (dd, *J* = 5.9, 0.9 Hz, 1H), 6.92 (t, *J* = 0.9 Hz, 1H), 1.62 (s, 9H). ^{13}C RMN (63 MHz, $CDCl_3$) δ 148.6 (Cq), 144.0 (CH), 142.0 (CH), 141.6 (Cq), 127.6 (Cq), 120.0 (CH), 110.2 (CH), 86.5 (Cq), 28.3 (3×CH_3). HRMS (ESI$^+$) calcd for $C_{12}H_{14}IN_2O_2$ (M+H$^+$) : 345.0095, found : 345.0093.

***tert*-Butyl 2-((4-(dimethylamino)phenyl)ethynyl)-1*H*-pyrrolo[3,2-*c*]pyridine-1-carboxylate 25**

The compound was prepared from *tert*-butyl 2-iodo-1*H*-pyrrolo[3,2-*c*]pyridine-1-carboxylate **24** using the general procedure B to afford **25** after flash chromatography on silica gel (EtOAc/Petroleum ether 10/90) as a pale yellow solid (99%). Mp 162-164°C. R_f = 0.44 (EtOAc/Petroleum ether 10/90). IR (ν, cm^{-1}, neat) 2983, 2205, 1733, 1604, 1549, 1456, 1338, 1247, 1149, 1092, 814, 507. ^{1}H RMN (400 MHz, $CDCl_3$) δ 8.82 (s, 1H), 8.45 (d, *J* = 5.8 Hz, 1H), 8.00 (d, *J* = 5.8 Hz, 1H), 7.43 (d, *J* = 7.9 Hz, 2H), 6.93 (s, 1H), 6.67 (d, *J* = 8.0 Hz, 2H), 3.00 (s, 6H), 1.69 (s, 9H). ^{13}C RMN (101 MHz, $CDCl_3$) δ 150.4 (Cq), 149.1 (Cq), 144.6 (CH), 143.2 (CH), 140.3 (Cq), 132.8 (2×CH),125.4 (Cq), 122.8 (Cq), 112.9 (CH), 111.7 (2×CH), 110.3 (CH), 109.1 (Cq), 97.3 (Cq),

85.1 (Cq), 79.3 (Cq), 40.1 (2×CH_3), 28.2 (3×CH_3). HRMS (ESI$^+$) calcd for $C_{22}H_{24}N_3O_2$ (M+H$^+$) : 362.1863, found : 362.1862.

2-Iodo-1*H*-pyrrolo[2,3-*b*]pyridine 26 [33]

The compound was prepared from 2-iodo-1-(phenylsulfonyl)-1*H*-pyrrolo[2,3-*b*]pyridine **8** using the general procedure C to afford **26** after flash chromatography on silica gel (EtOAc/Petroleum ether 10/90) as a white solid (85%). Mp 189-191°C. R_f = 0.43 (EtOAc/Petroleum ether 20/80). IR (ν, cm^{-1}, neat) 2790, 1580, 1481, 1431, 1403, 1336, 1308, 1275, 911, 812, 762, 622, 510. ^{1}H RMN (400 MHz, DMSO-d$_6$) δ 12.20 (s, 1H), 8.13 (dd, *J* = 4.7, 1.6 Hz, 1H), 7.86 (dd, *J* = 7.8, 1.6 Hz, 1H), 7.02 (dd, *J* = 7.8, 4.7 Hz, 1H), 6.70 (s, 1H). ^{13}C RMN (101 MHz, DMSO-*d*$_6$) δ 151.0 (Cq), 142.9 (CH), 126.9 (CH), 122.1 (Cq), 116.3 (CH), 110.0 (CH), 81.1 (Cq). HRMS (ESI$^+$) calcd for $C_7H_6IN_2$ (M+H$^+$) : 244.9570, found : 244.9570.

***tert*-Butyl 2-iodo-1*H*-pyrrolo[2,3-*b*]pyridine-1-carboxylate 27**

The compound was prepared from **26** using the general procedure E to afford **27** after flash chromatography on silica gel (EtOAc/Petroleum ether 10/90) as a white solid (99%). Mp 74-76°C.R_f = 0.40 (EtOAc/Petroleum ether 20/80). IR (ν, cm^{-1}, neat) 3067, 2981, 1725, 1504, 1460, 1392, , 1206, 1132, 1044, 912, 839, 765, 665, 589, 511. ^{1}H RMN (400 MHz, $CDCl_3$) δ 8.36 (dd, *J* = 4.8, 1.6 Hz, 1H), 7.72 (dd, *J* = 7.8, 1.6 Hz, 1H), 7.08 (dd, *J* = 7.8, 4.8 Hz, 1H), 6.87 (s, 1H), 1.70 (s, 9H). ^{13}C RMN (101 MHz, $CDCl_3$) δ 149.65 (Cq), 147.67 (Cq), 144.74 (CH), 127.30 (CH), 123.28 (Cq), 118.59 (CH), 118.02 (CH), 85.84 (Cq), 76.99 (Cq), 28.20 (3×CH_3). HRMS (ESI$^+$) calcd for $C_{12}H_{14}IN_2O_2$ (M+H$^+$) : 345.0095, found : 345.0094.

***tert*-Butyl 2-((4-(methylamino)phenyl)ethynyl)-1*H*-pyrrolo[2,3-*b*]pyridine-1-carboxylate 28**

The compound was prepared from *tert*-butyl 2-iodo-1*H*-pyrrolo[2,3-*c*]pyridine-1-carboxylate **27** using the general procedure B to afford **28** after flash chromatography on silica gel (EtOAc/Petroleum ether 20/80) as a pale yellow solid (91%). Mp 166-168°C. R_f = 0.34 (EtOAc/Petroleum ether 20/80). IR (ν, cm^{-1}, neat) 3368, 3066, 2983, 2815, 2198, 1742, 1607, 1575, 1404, 1367, 1251, 1177, 1087, 876, 772, 640, 561, 515. ^{1}H RMN (400 MHz, $CDCl_3$) δ 8.53 (d, *J* = 3.9 Hz, 1H), 7.83 (d, *J* = 7.7 Hz, 1H), 7.40 (d, *J* = 8.3 Hz, 2H), 7.19 (dd, *J* = 7.5, 4.9 Hz, 1H), 6.81 (s, 1H), 6.59 (d, *J* = 8.3 Hz, 2H), 4.02 (ls, 1H), 2.89 (s, 3H), 1.70 (s, 9H). ^{13}C RMN (101 MHz, $CDCl_3$) δ 149.6 (Cq), 148.6 (Cq), 148.1 (Cq), 145.7 (CH), 132.9 (2×CH), 128.3 (CH), 122.0 (Cq), 121.6 (Cq),

118.8 (CH), 112.0 (2×CH), 111.6 (CH), 110.2 (Cq), 97.0 (Cq), 84.6 (Cq), 79.5 (Cq), 30.3 (CH_3), 28.2 (3×CH_3). HRMS (ESI+) calcd for $C_{21}H_{22}N_3O_2$ (M+H+) : 348.1707, found : 348.1705.

***tert*-Butyl 2-((4-((2-hydroxyethyl)(methyl)amino)phenyl)ethynyl)-1*H*-pyrrolo[2,3-*b*]pyridine-1-carboxylate 29**

To a solution of **28** (0.6 g, 1.73 mmol, 1.0 equiv.), 2-bromoethanol (0.55 mL, 7.77 mmol, 4.5 equiv.) and KI (0.06 g, 0.35 mmol, 0.2 equiv.) in CH_3CN (30 mL) was added a 1M solution if *t*BuOK in THF (3.8 mL, 3.80 mmol, 2.2 equiv.). The reaction mixture was stirred at r.t. for 8 days and 4.5 equiv. of 2-bromoethanol was additionally added each day. Water (30 mL) was added and the reaction mixture was extracted with EtOAc (3x30 mL). The combined organic layers were successively washed with water (20 mL), brine (20 mL) and dried over $MgSO_4$. After filtration and concentration under reduced pressure, the crude material was purified by flash chromatography under silica gel (EtOAc/Petroleum ether 50/50 + MeOH1%) to afford **29** as a pale yellow solid (209 mg, 31%). Mp 154-156°C. R_f = 0.22 (EtOAc/Petroleum ether 40/60). IR (ν, cm^{-1}, neat) 3386, 2980, 2926, 2206, 1747, 1603, 1546, 1473, 1384, 1249, 1194, 1085, 980, 847, 776, 643, 525. ^{1}H RMN (250 MHz, $CDCl_3$) δ 8.50 (dd, *J* = 4.8, 1.7 Hz, 1H), 7.81 (dd, *J* = 7.8, 1.7 Hz, 1H), 7.46-7.34 (m, 2H), 7.17 (dd, *J* = 7.8, 4.8 Hz, 1H), 6.80 (s, 1H), 6.79-6.64 (m, 2H), 3.85 (t, *J* = 5.7 Hz, 2H), 3.54 (t, *J* = 5.7 Hz, 2H), 3.04 (s, 3H), 1.82 (ls, 1H), 1.68 (s, 9H). ^{13}C RMN (63 MHz, $CDCl_3$) δ 149.8 (Cq), 148.6 (CH), 148.1 (Cq), 145.7 (Cq), 132.8 (2×CH), 128.3 (CH), 121.9 (Cq), 121.6 (Cq), 118.8 (CH), 112.1 (2×CH), 111.7 (CH), 109.9 (Cq), 96.9 (Cq), 84.6 (Cq), 79.8 (Cq), 60.2 (CH_2), 54.8 (CH_2), 38.9 (CH_3), 28.2 (3×CH_3). HRMS (ESI+) calcd for $C_{23}H_{26}N_3O_3$ (M+H+) : 392.1969, found : 392.1968.

2-(Phenylethynyl)-1*H*-indole 31 [34]

The compound was prepared from **2** using the general procedure C to afford **31** after flash chromatography on silica gel (EtOAc/Petroleum ether 2/98) as a white solid (91%). Mp 161-163°C. R_f = 0.53 (EtOAc/Petroleum ether 10/90). ^{1}H RMN (400 MHz, acetone-d_6) δ 10.71 (large s, 1H), 7.63-7.53 (m, 3H), 7.48-7.41 (m, 4H), 7.21 (m, 1H), 7.09 (td, *J* = 7.6, 0.8 Hz, 1H), 6.84 (s, 1H).

2-(*p*-Tolylethynyl)-1*H*-indole 32 [26]

The compound was prepared from **3** using the general procedure C to afford **32** after flash chromatography on silica gel (EtOAc/Petroleum ether 2/98) as a white solid (91%). Mp 179-

181°C, R_f = 0.53 (EtOAc/Petroleum ether 10/90). ^{1}H RMN (400 MHz, acetone-d_6) δ 10.66 (br s, 1H), 7.58 (d, *J* = 8.0 Hz, 1H), 7.46-7.39 (m, 3H), 7.28-7.16 (m, 3H), 7.07 (t, *J* = 7.5 Hz, 1H), 6.80 (m, 1H), 2.36 (s, 3H).

2-((4-Ethylphenyl)ethynyl)-1*H*-indole 33

The compound was prepared from **4** using the general procedure C to afford **33** after flash chromatography on silica gel (EtOAc/Petroleum ether 20/80) as a white solid (58%). Mp 131-133°C. R_f = 0.27 (EtOAc/Petroleum ether 20/80). IR (ν, cm^{-1}, neat) 3393, 3051, 2965, 1538, 1450, 1399, 1235, 1117, 825, 792, 652, 522, 507. ^{1}H RMN (250 MHz, $CDCl_3$) δ 11.66 (s, 1H), 7.63-7.43 (m, 3H), 7.39-7.22 (m, 3H), 7.16 (t, *J* = 7.3 Hz, 1H), 7.03 (t, *J* = 7.4 Hz, 1H), 6.79 (s, 1H), 2.65 (q, *J* = 7.4 Hz, 2H), 1.19 (t, *J* = 7.4 Hz, 3H). ^{13}C RMN (63 MHz, $CDCl_3$) δ 145.6 (Cq), 136.6 (Cq), 131.6 (2×CH), 129.1 (2×CH), 128.5 (Cq), 123.9 (Cq), 121.2 (CH), 120.7 (CH), 119.7 (Cq), 119.5 (Cq), 113.7 (CH), 107.8 (CH) 97.2 (Cq), 84.9 (Cq), 25.1 (CH_2), 15.6 (CH_3). HRMS (ESI$^+$) calcd for $C_{18}H_{16}N$ (M+H$^+$) : 246.1277, found : 246.1279.

2-((4-Methoxyphenyl)ethynyl)-1*H*-indole 34 [35]

The compound was prepared from **5** using the general procedure C to afford **34** after flash chromatography on silica gel (EtOAc/Petroleum ether 7/93) as a white solid (83%). Mp 163-165°C. R_f = 0.41 (EtOAc/Petroleum ether 10/90). IR (ν, cm^{-1}, neat) 3414, 3051, 2944, 2839, 1602, 1575, 1438, 1397, 1290, 1171, 1027, 958, 893, 798, 650, 610. ^{1}H RMN (250 MHz, acetone-d_6) δ 10.63 (br s, 1H), 7.59 (d, *J* = 7.6 Hz, 1H), 7.49 (d, 2H, *J* = 8.8 Hz, 1H), 7.42 (m, 1H), 7.19 (t, *J* = 7.6 Hz, 1H), 7.07 (m, 1H), 7.01-6.90 (m, 2H), 6.78 (m, 1H), 3.83 (s, 3H).

4-((1*H*-Indol-2-yl)ethynyl)aniline 35

The compound was prepared from **9** using the general procedure C to afford **35** after flash chromatography on silica gel (EtOAc/Petroleum ether 30/70) as a white solid (79%). Mp 200-202°C. R_f = 0.45 (EtOAc/Petroleum ether 30/70). IR (ν, cm^{-1}, neat) 3449, 3382, 3210, 2205, 1604, 1574, 1448, 1395, 1233, 1150, 1005, 935, 850, 827, 725, 701, 651, 607. ^{1}H RMN (400 MHz, DMSO-d_6)) δ 11.52 (d, *J* = 2.3 Hz, 1H), 7.50 (dd, *J* = 8.1, 1.2 Hz, 1H), 7.30 (dd, *J* = 8.3, 1.1 Hz, 1H), 7.25-7.19 (m, 2H), 7.12 (ddd, *J* = 8.3, 7.0, 1.2 Hz, 1H), 7.01 (ddd, *J* = 8.1, 7.0, 1.1 Hz, 1H), 6.67 (dd, *J* = 2.2, 1.0 Hz, 1H), 6.61-6.54 (m, 2H), 5.61 (s, 2H). ^{13}C RMN (101 MHz, DMSO-d_6) δ 150.2 (Cq), 136.7 (Cq), 133.0 (2×CH), 127.9 (Cq), 122.8 (CH), 120.5 (CH), 120.0 (CH), 119.8 (Cq), 114.2

(2×CH), 111.5 (CH), 108.1 (Cq), 106.7 (CH), 93.9 (Cq), 80.2 (Cq). HRMS (ESI$^+$) calcd for $C_{16}H_{13}N_2$ (M+H$^+$) : 233.1073, found : 233.1075.

3-((1*H*-Indol-2-yl)ethynyl)aniline 36

The compound was prepared from **10** using the general procedure C to afford **36** after flash chromatography on silica gel (EtOAc/Petroleum ether 25/75) as a white solid (99%). Mp 163-165°C. R_f = 0.35 (EtOAc/Petroleum ether 30/70). IR (ν, cm^{-1}, neat) 3395, 3319, 3122, 2205, 1598, 1486, 1357, 1233, 1187, 992, 929, 874, 781, 736, 608. ^{1}H RMN (400 MHz, acetone-*d$_6$*) δ 10.66 (large s, 1H), 7.59 (d, *J* = 8.1 Hz, 1H), 7.43 (dd, *J* = 8.2, 1.2 Hz, 1H), 7.21 (ddd, *J* = 8.2, 7.0, 1.2 Hz, 1H), 7.15-7.04 (m, 2H), 6.90 (t, *J* = 1.9 Hz, 1H), 6.82 (m, 2H), 6.74 (ddd, *J* = 8.1, 2.4, 1.1 Hz, 1H), 4.82 (large s, 2H). ^{13}C RMN (101 MHz, acetone-*d$_6$*) δ 148.6 (Cq), 136.8 (Cq), 129.3 (CH), 127.9 (Cq), 123.0 (CH), 123.0 (Cq), 120.4 (CH), 120.0 (CH), 119.8 (CH), 119.1 (Cq), 116.5 (CH), 115.0 (CH), 111.1 (CH), 107.7 (CH), 92.6 (Cq), 81.0 (Cq). HRMS (ESI$^+$) calcd for $C_{16}H_{13}N_2$ (M+H$^+$) : 233.1073, found : 233.1077.

4-((1*H*-Indol-2-yl)ethynyl)-*N*-methylaniline 37

The compound was prepared from **11** using the general procedure C to afford **37** after flash chromatography on silica gel (EtOAc/Petroleum ether 20/80) as a white solid (99%). Mp189-191°C. R_f = 0.38 (EtOAc/Petroleum ether 20/80). IR (ν, cm^{-1}, neat) 3411, 3053, 2897, 2200, 1605, 1577, 1451, 1395, 1264, 1178, 1063, 930, 853, 794, 650, 610. ^{1}H RMN (400 MHz, DMSO-*d$_6$*) δ 11.53 (s, 1H), 7.51 (d, *J* = 7.9 Hz, 1H), 7.31 (m, 3H), 7.13 (t, *J* = 7.3 Hz, 1H), 7.02 (t, *J* = 7.3 Hz, 1H), 6.68 (d, *J* = 1.1 Hz, 1H), 6.57 (d, *J* = 8.6 Hz, 2H), 6.20 (d, *J* = 5.0 Hz, 1H), 2.72 (d, *J* = 5.0 Hz, 3H). ^{13}C RMN (101 MHz, DMSO- *d$_6$*) δ 150.8 (Cq), 136.7 (Cq), 132.9 (2×CH), 127.9 (Cq), 122.8 (CH), 120.5 (CH), 120.0 (CH), 119.8 (Cq), 112.0 (2×CH), 111.5 (CH), 107.9 (Cq), 106.7 (CH), 93.9 (Cq), 80.4 (Cq), 29.8 (CH_3). HRMS (ESI$^+$) calcd for $C_{17}H_{15}N_2$ (M+H$^+$) : 247.1230, found : 247.1232.

4-((1*H*-Indol-2-yl)ethynyl)-*N*,*N*-dimethylaniline 38.

The compound was prepared from **12** using the general procedure C to afford **38** after flash chromatography on silica gel (EtOAc/Petroleum ether 20/80) as a white solid (77%). Mp 209-211°C. R_f = 0.33 (EtOAc/Petroleum ether 10/90). IR (ν, cm^{-1}, neat) 3379, 2202, 1601, 1538, 1448, 1395, 1367, 12281186, 1063, 978, 817, 718, 611. ^{1}H RMN (400 MHz, DMSO-*d$_6$*) δ 11.55 (s, 1H), 7.51 (d, *J* = 8.0 Hz, 1H), 7.38 (d, *J* = 8.8 Hz, 2H), 7.31 (d, *J* = 8.0 Hz, 1H), 7.13 (t, *J* = 7.5 Hz, 1H),

7.02 (t, J = 7.4 Hz, 1H), 6.73 (d, J = 8.9 Hz, 2H), 6.70 (d, J = 1.4 Hz, 1H), 2.96 (s, 6H). ^{13}C RMN (100 MHz, DMSO-d_6) δ 150.2 (Cq), 136.3 (Cq), 132.3 (2×CH), 127.4 (Cq), 122.4 (CH), 120.0 (CH), 119.6 (CH), 119.2 (Cq), 111.9 (2×CH), 111.0 (CH), 107.9 (Cq), 106.4 (CH), 93.1 (Cq), 80.5 (Cq), 39.7 (2×CH_3). HRMS (ESI$^+$) calcd for $C_{18}H_{17}N_2$ (M+H$^+$) : 261.1386, found : 261.1389.

4-((5-Methoxy-1*H*-indol-2-yl)ethynyl)-*N,N*-dimethylaniline 39

The compound was prepared from **13** using the general procedure C to afford **39** after flash chromatography on silica gel (EtOAc/Petroleum ether 10/90) as a white solid (88%). Mp 213-215°C, R_f = 0.33 (EtOAc/Petroleum ether 10/90). IR (ν, cm^{-1}, neat) 3379, 2202, 1601, 1538, 1448, 1395, 1203, 1186, 1168, 1063, 929, 817, 718, 699, 654, 611. ^{1}H RMN (400 MHz, DMSO- d_6) δ 11.81 (m, 1H), 7.37 (d, J = 8.7 Hz, 2H), 7.21 (d, J = 8.8 Hz, 1H), 7.00 (d, J = 2.5 Hz, 1H), 6.79 (dd, J = 8.8, 2.5 Hz, 1H), 6.73 (d, J = 8.8 Hz, 2H), 6.62 (m, 1H), 3.75 (s, 3H), 2.96 (s, 6H). ^{13}C RMN (100 MHz, DMSO- d_6) δ 154.2 (Cq), 150.6 (Cq), 132.7 (2×CH), 131.9 (Cq), 128.2 (Cq), 120.0 (Cq), 113.5 (CH), 112.4 (2×CH), 112.2 (CH), 108.5 (Cq), 106.7 (CH), 101.6 (CH), 93.4 (Cq), 81.1 (Cq), 55.7 (CH_3), 40.2 (2CH_3). HRMS (ESI$^+$) calcd for $C_{19}H_{19}N_2O$ (M+H$^+$) : 291.1492, found : 291.1495.

4-((5-(2-Fluoroethoxy)-1*H*-indol-2-yl)ethynyl)-*N,N*-dimethylaniline 40

The compound was prepared from **8** using the general procedure B to afford **14** after filtration through celite (66%). The crude was directly engaged in the general procedure C to afford **40** after flash chromatography on silica gel (EtOAc/Petroleum ether 10/90) as a yellow solid (86%). Mp 174-176°C. R_f = 0.40 (EtOAc/Petroleum ether 30/70). IR (ν, cm^{-1}, neat) 3406, 2921, 2202, 1604, 1583, 1541, 1511, 1481, 1445, 1396, 1364, 1298, 1260, 1218, 1192, 1171, 1112, 1070, 1051, 962, 944, 902, 885, 819, 654, 617, 521. ^{1}H RMN (400 MHz, DMSO- d_6) δ 11.43 (s, 1H), 7.37 (d, J = 8.9 Hz, 2H), 7.22 (d, J = 8.8 Hz, 1H), 7.04 (d, J = 2.3 Hz, 1H), 6.83 (dd, J = 8.8, 2.3 Hz, 1H), 6.73 (d, J = 8.9 Hz, 2H), 6.61 (d, J = 1.3 Hz, 1H), 4.81 (m, 1H), 4.69 (m, 1H), 4.25 (m, 1H), 4.17 (m, 1H), 2.96 (s, 6H). ^{13}C RMN (101 MHz, DMSO- d_6) δ 153.0 (Cq), 150.7 (Cq), 132.7 (2×CH), 132.1 (Cq), 128.2 (Cq), 120.2 (Cq), 113.8 (CH), 112.4 (2×CH), 112.3 (CH), 108.5 (Cq), 106.7 (CH), 102.9 (CH), 93.5 (Cq), 82.8 (d, J = 165.5 Hz, CH_2), 81.0 (Cq), 68.0 (d, J = 19.1 Hz, CH_2), 40.2 (2×CH_3). ^{19}F RMN (376 MHz, DMSO- d_6) δ -221.7 (tt, J = 48.0, 30.7 Hz). HRMS (ESI$^+$) calcd for $C_{20}H_{20}FN_2O$ (M+H$^+$) : 323.1554, found : 323.1557.

4-((1*H*-Pyrrolo[3,2-*b*]pyridin-2-yl)ethynyl)-*N,N*-dimethylaniline 41

The compound was prepared from 2-iodo-1-(phenylsulfonyl)-1H-pyrrolo[3,2-b]pyridine **15** using the general procedure B to afford **19** after filtration through celite. The crude was directly engaged in the general procedure C to afford **41** after flash chromatography on silica gel (EtOAc/Petroleum ether 30/70) as an orange solid (87%). Mp 191-193°C. R_f = 0.24 (EtOAc/Petroleum ether 30/70). IR (ν, cm^{-1}, neat) 3064, 2889, 2806, 2703, 2199, 1673, 1512, 1406, 1355, 1223, 1186, 913, 814, 778, 623, 558, 514. ^1H RMN (400 MHz, DMSO-d_6) δ 11.82 (s, 1H), 8.35 (dd, *J* = 4.6, 1.2 Hz, 1H), 7.69 (d, *J* = 8.1 Hz, 1H), 7.40 (t, *J* = 8.9 Hz, 2H), 7.14 (dd, *J* = 8.2, 4.6 Hz, 1H), 6.81 (d, *J* = 0.9 Hz, 1H), 6.75 (d, *J* = 8.9 Hz, 2H), 2.97 (s, 6H). ^{13}C RMN (101 MHz, DMSO-d_6) δ 150.9 (Cq), 145.9 (Cq), 143.8 (CH), 132.9 (2×CH), 129.7 (Cq), 123.2 (Cq), 118.4 (CH), 117.8 (CH), 112.4 (2×CH), 107.8 (Cq), 106.8 (CH), 95.4 (Cq), 80.4 (Cq), 40.1 (2×CH_3). HRMS (ESI^+) calcd for $C_{17}H_{16}N_3$ ($M+H^+$) : 262.1339, found : 262.1342.

4-((1*H*-Pyrrolo[2,3-*c*]pyridin-2-yl)ethynyl)-*N,N*-dimethylaniline 42

The compound was prepared from **21** using the general procedure C to afford **42** after flash chromatography on silica gel (EtOAc/Petroleum ether 20/80) as a yellow solid (88%). Mp 242-244°C. R_f = 0.27 (EtOAc/Petroleum ether 30/70). IR (ν, cm^{-1}, neat) 2669, 2207, 1609, 1573, 1361, 1224, 1187, 906, 811, 598, 515. ^1H RMN (250 MHz, DMSO-d_6) δ 12.08 (large s, 1H), 8.67 (s, 1H), 8.10 (d, *J* = 5.5 Hz, 1H), 7.49 (dd, *J* = 5.5, 1.0 Hz, 1H), 7.45-7.36 (m, 2H), 6.79-6.71 (m, 3H), 2.98 (s, 6H). ^{13}C RMN (63 MHz, DMSO-d_6) δ 151.0, 138.6 (CH), 134.4 (CH), 133.0 (2×CH), 132.2 (Cq), 123.6 (Cq) , 118.2 (CH), 117.3 (Cq) 112.4 (2×CH), 107.5 (Cq), 105.8 (CH), 95.1 (Cq), 80.1 (Cq), 40.1 (2×CH_3). HRMS (ESI^+) calcd for $C_{17}H_{16}N_3$ ($M+H^+$) : 262.1339, found : 262.1338.

4-((1*H*-Pyrrolo[2,3-b]pyridin-2-yl)ethynyl)-*N,N*-dimethylaniline 43

The compound was prepared from **22** using the general procedure C to afford **43** after flash chromatography on silica gel (EtOAc/Petroleum ether 15/85) as a yellow solid (92%). Mp 229-231°C, R_f = 0.36 (EtOAc/Petroleum ether 30/70). IR (ν, cm^{-1}, neat) 3055, 2886, 2198, 1603, 1584, 1405, 1326, 1223, 1186, 1061, 976, 807, 763, 625. RMN ^1H (400 MHz, DMSO-d_6) δ 12.07 (s, 1H), 8.20 (m, 1H), 7.92 (dd, *J* = 7.8, 1.7 Hz, 1H), 7.39 (d, *J* = 8.4 Hz, 2H), 7.08 (dd, *J* = 7.8, 4.6 Hz, 1H), 6.84-6.46 (m, 3H), 2.97 (s, 6H). RMN ^{13}C (100 MHz, DMSO-*d6*) δ 150.8 (Cq), 148.8 (Cq), 144.3 (CH), 132.9 (2×CH), 128.4 (CH), 120.6 (Cq), 120.2 (Cq), 116.6 (CH), 112.4 (2×CH), 108.0 (Cq), 105.4 (CH), 94.9 (Cq), 80.5 (Cq), 40.1 (2×CH_3). HRMS (ESI^+) calcd for $C_{17}H_{16}N_3$ ($M+H^+$) : 262.1339, found : 262.1340.

4-((1*H*-Pyrrolo[3,2-*c*]pyridin-2-yl)ethynyl)-*N*,*N*-dimethylaniline 44

The compound was prepared from **25** using the general procedure E to afford **44** after flash chromatography on silica gel (EtOAc) as an orange solid (47%). Mp 250-252°C. R_f = 0.22 (EtOAc). IR (ν, cm^{-1}, neat) 2899, 2623, 2203, 1605, 1543, 13691233, 1182, 814, 762, 519, 506. ^{1}H RMN (400 MHz, DMSO-d_6) δ 11.99 (s, 1H), 8.80 (s, 1H), 8.19 (d, *J* = 5.7 Hz, 1H), 7.43-7.36 (m, 2H), 7.29 (dt, *J* = 5.8 Hz, *J* =1.0 Hz, 1H), 6.84 (s, 1H), 6.78-6.68 (m, 2H, 2.97 (s, 6H). ^{13}C RMN (101 MHz, DMSO-d_6) δ 150.8 (Cq), 143.5 (CH), 141.58 (CH), 139.8 (Cq), 132.9 (2×CH), 125.0 (Cq), 121.0 (Cq), 112.3 (2×CH), 107.8 (Cq), 106.7 (CH), 105.8 (CH), 94.4 (Cq), 80. (Cq)1, 40.1 (2×CH_3). HRMS (ESI$^+$**)** calcd for $C_{17}H_{16}N_3$ (M+H$^+$) : 262.1339, found : 262.1340.

4-((1*H*-Pyrrolo[2,3-*b*]pyridin-2-yl)ethynyl)-*N*-(2-fluoroethyl)-*N*-methylaniline 45

To a solution of **29** (0.115 g, 0.29 mmol,) in CH_2Cl_2 (3 mL) at -78°C and under argon, DAST (0.077 mL, 0.59 mmol, 2.0 equiv.) was added dropwise. After 1h, temperature was allowed to return to r.t. and water (10 mL) was added. The aqueous layer was washed with CH_2Cl_2 (2x10 mL) and the combined organic layers dried over $MgSO_4$ and concentrated under reduced pressure. The crude material was filtrated on silica gel (EtOAc/Petroleum ether 20/80) to afford **30** as a white solid (25 mg, 22%). The compound was directly engaged in the general procedure E to afford **45** after flash chromatography on silica gel (EtOAc/Petroleum ether 10/90) as a yellow solid (83%). Mp 242-244°C. R_f = 0.28 (EtOAc/Petroleum ether 20/80). IR (ν, cm^{-1}, neat) 3113, 3057, 2977, 2892, 2360, 2202, 1601, 1510, 1432, 1378, 1214, 1137, 1076, 1042, 977, 810, 692, 515. ^{1}H RMN (400 MHz, DMSO-d_6) δ 12.07 (s, 1H), 8.24 (m, 1H), 7.90 (m, 1H), 7.39 (d, *J* = 8.4 Hz, 2H), 7.08 (dd, *J* = 7.9, 4.6 Hz, 1H), 6.78 (d, *J* = 8.4 Hz, 2H), 6.71 (d, *J* = 1.7 Hz, 1H), 4.66 (t, *J* = 5.0 Hz, 1H), 4.54 (t, *J* = 5.0 Hz, 1H), 3.76 (t, *J* = 5.1 Hz, 1H), 3.69 (t, *J* = 5.1 Hz, 1H), 3.00 (s, 3H). ^{13}C RMN (101 MHz, DMSO-d_6) δ 149.7 (Cq), 148.8 (Cq), 144.3 (CH), 133.0 (2×CH), 128.4 (CH), 120.5 (Cq), 120.2 (Cq), 116.6 (CH), 112.4 (2×CH), 108.3 (Cq), 105.5 (CH), 94.7 (Cq), 82.4 (d, *J* = 166.2 Hz, CH_2), 80.5 (Cq), 51.9 (d, *J* = 19.8 Hz, CH_2), 38.9 (CH_3). ^{19}F RMN (235 MHz, DMSO-d_6) δ -221.28 (tt, *J* = 47.5, 26.2 Hz). HRMS (ESI$^+$) calcd for $C_{18}H_{17}FN_3$ (M+H$^+$) : 294.1401, found : 294.1401.

2-(Phenylethynyl)-1-(phenylsulfonyl)-1*H*-indole 2 [23]

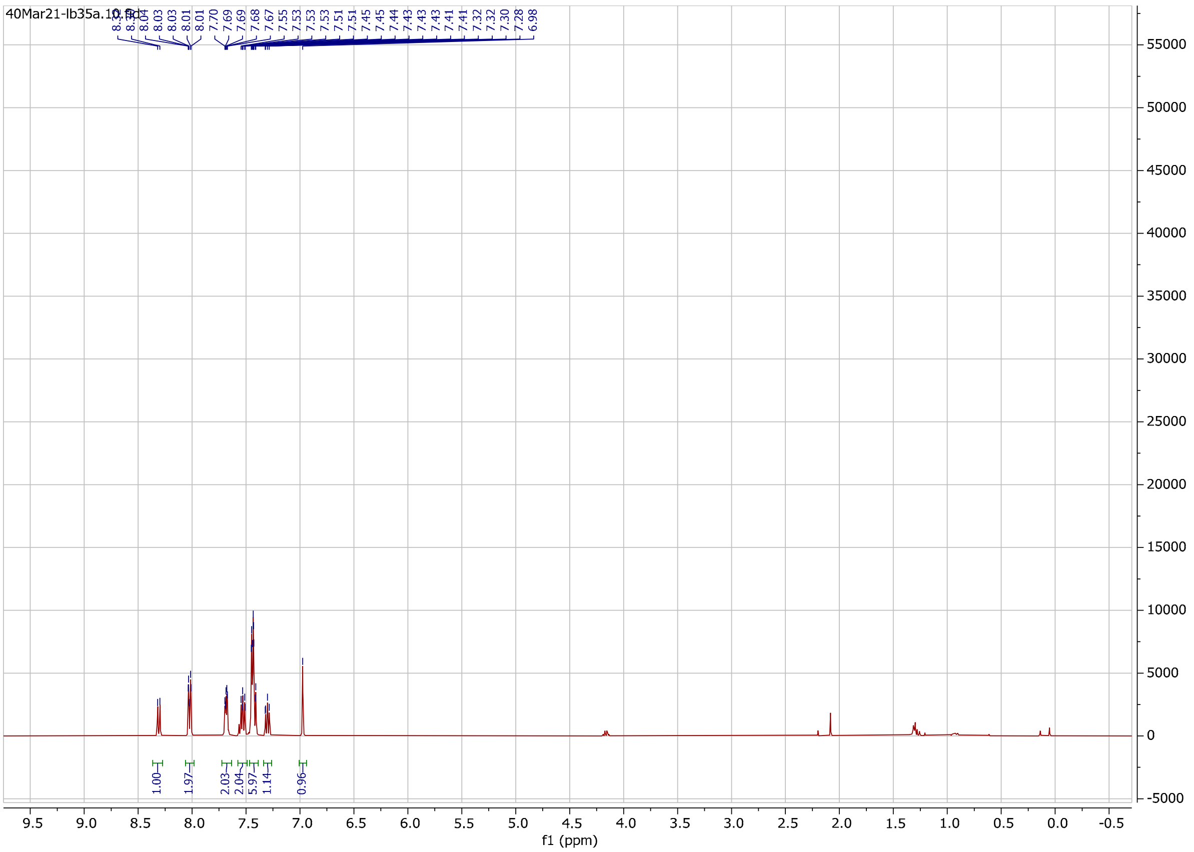

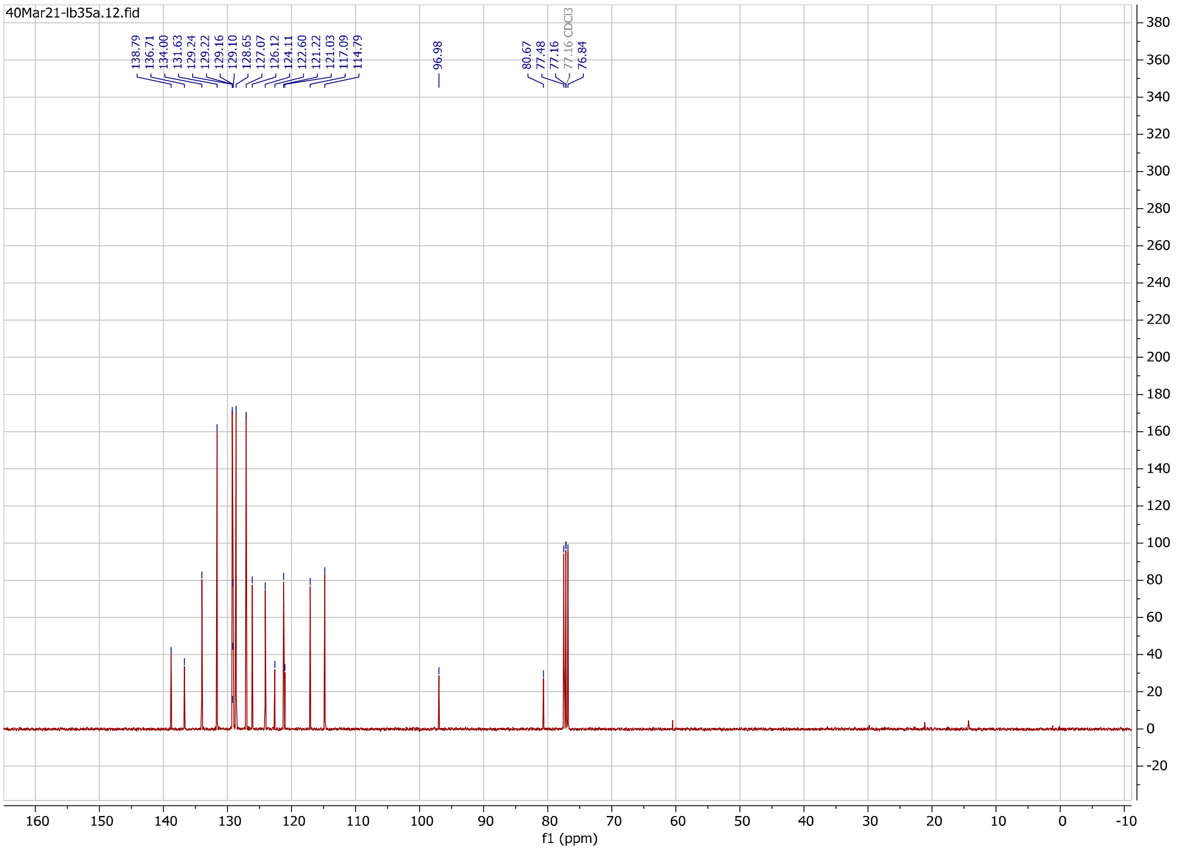

1-(Phenylsulfonyl)-2-(*p*-tolylethynyl)-1*H*-indole 3

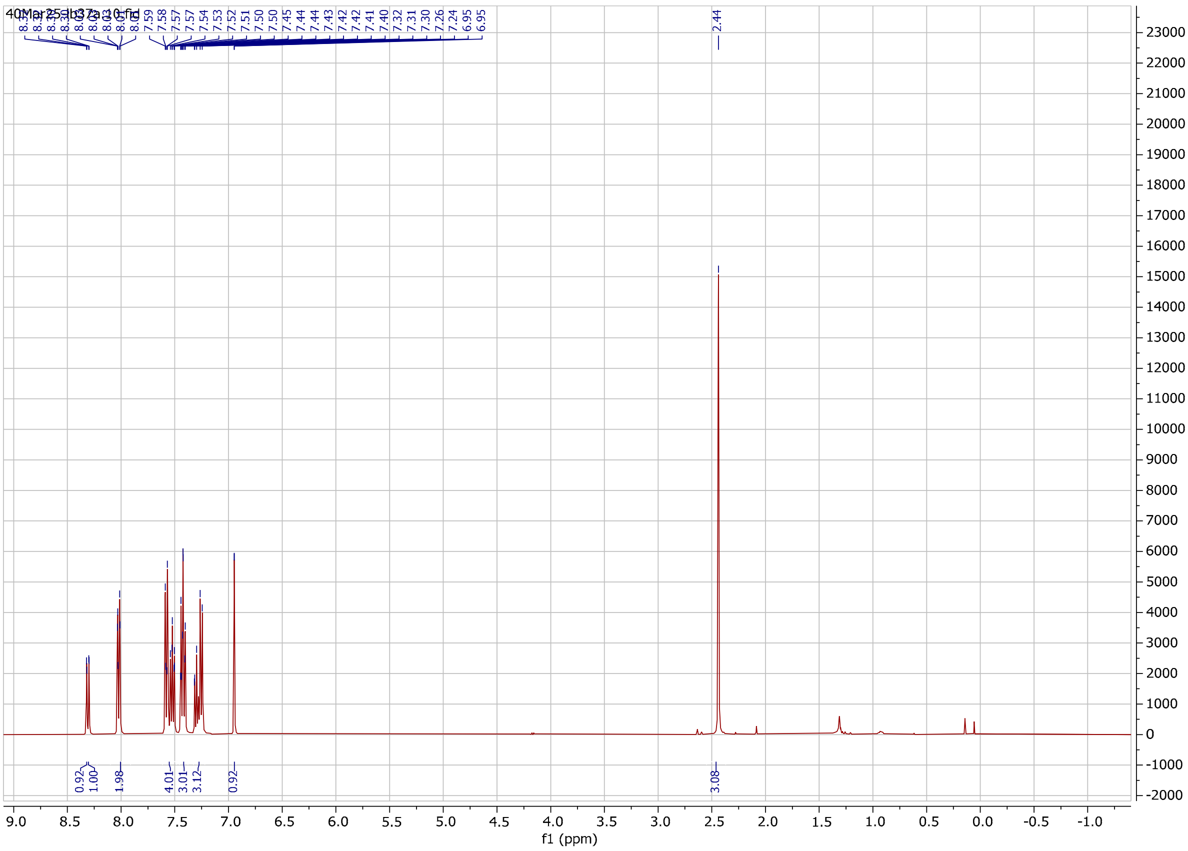

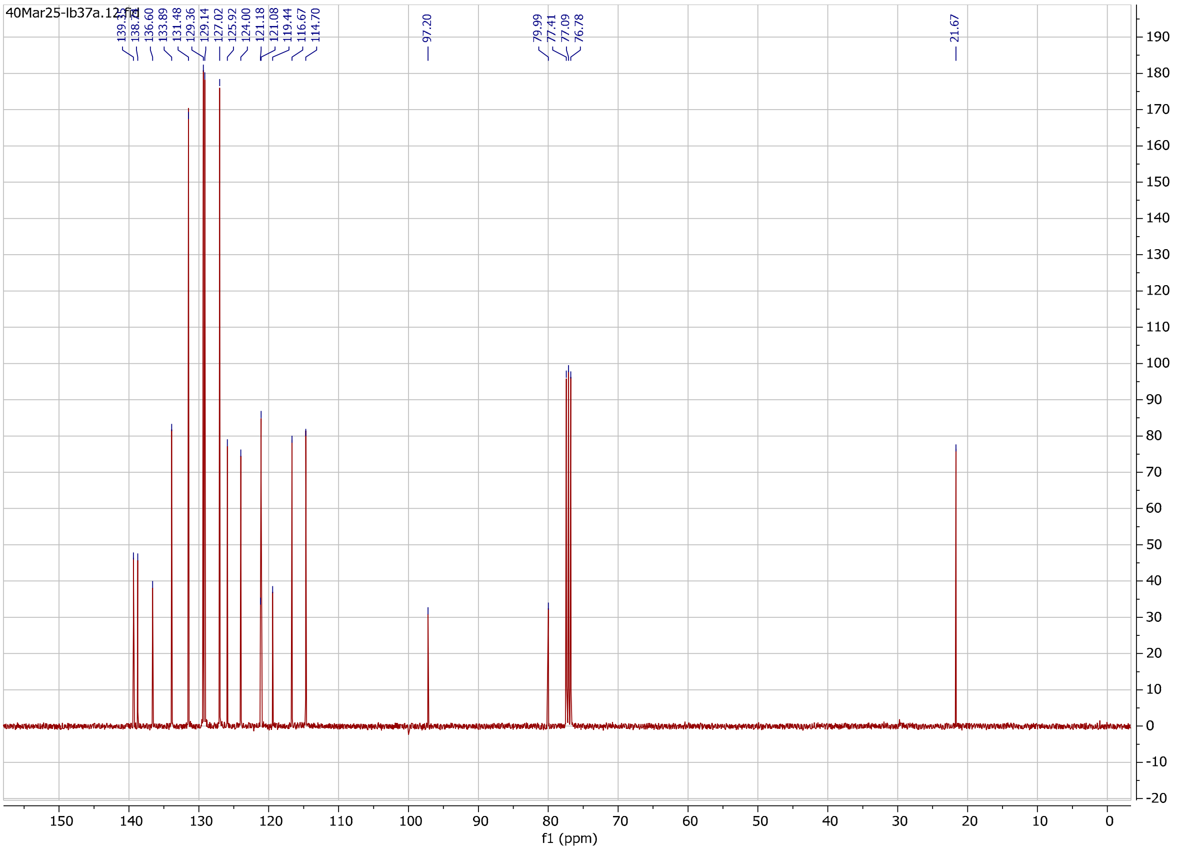

2-((4-Ethylphenyl)ethynyl)-1-(phenylsulfonyl)-1*H*-indole 4

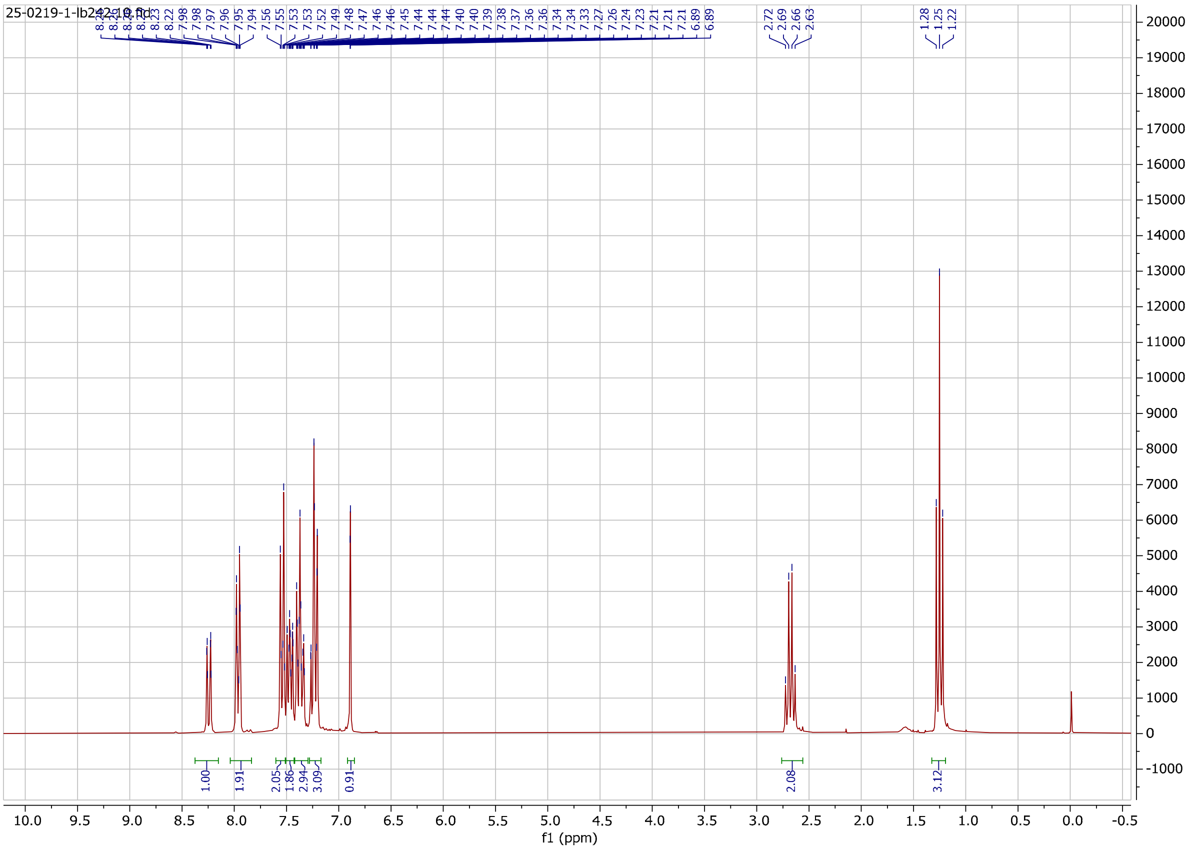

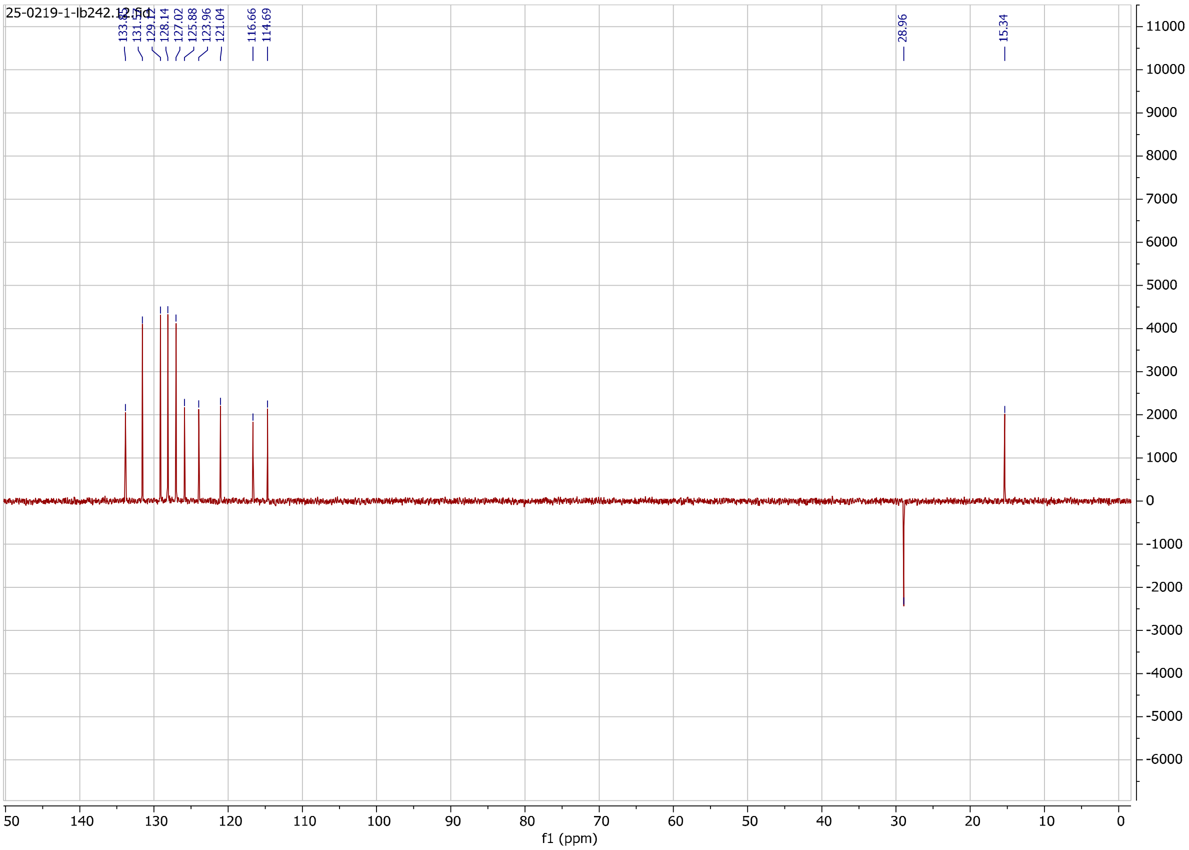

2-((4-Methoxyphenyl)ethynyl)-1-(phenylsulfonyl)-1*H*-indole 5

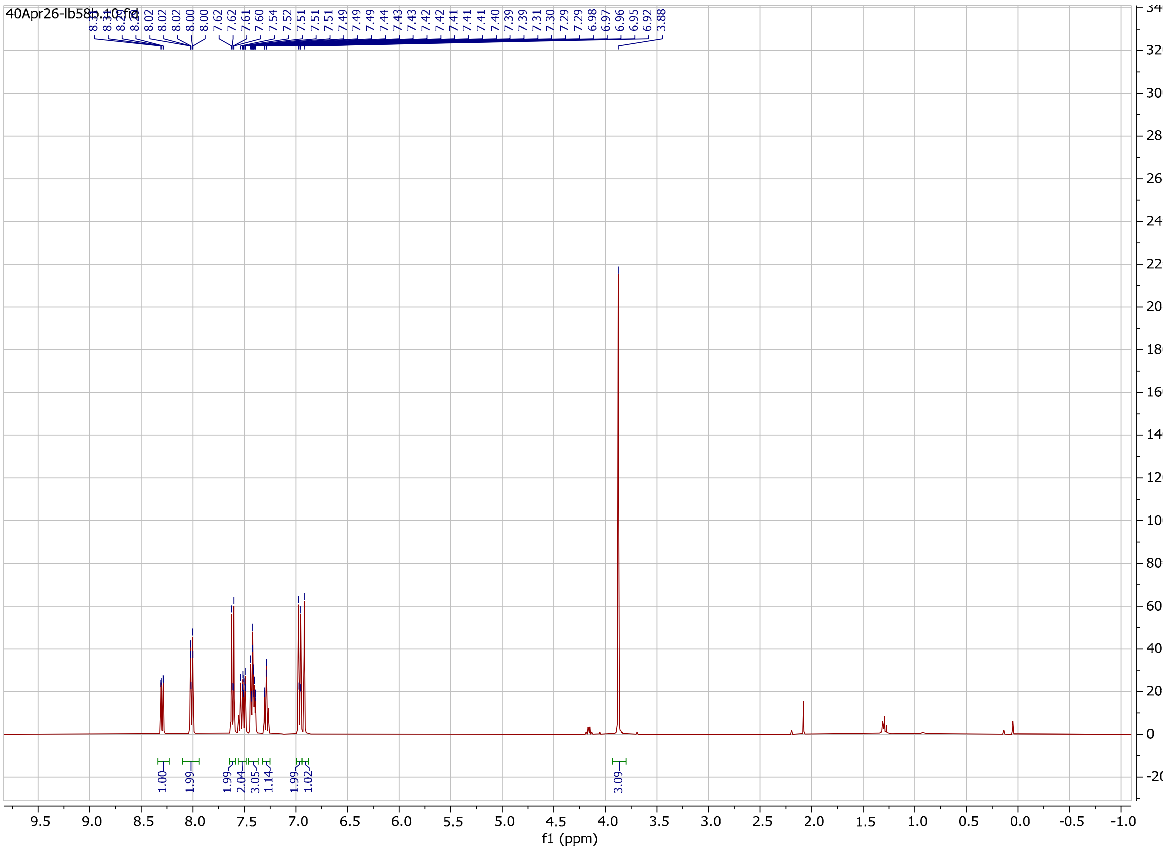

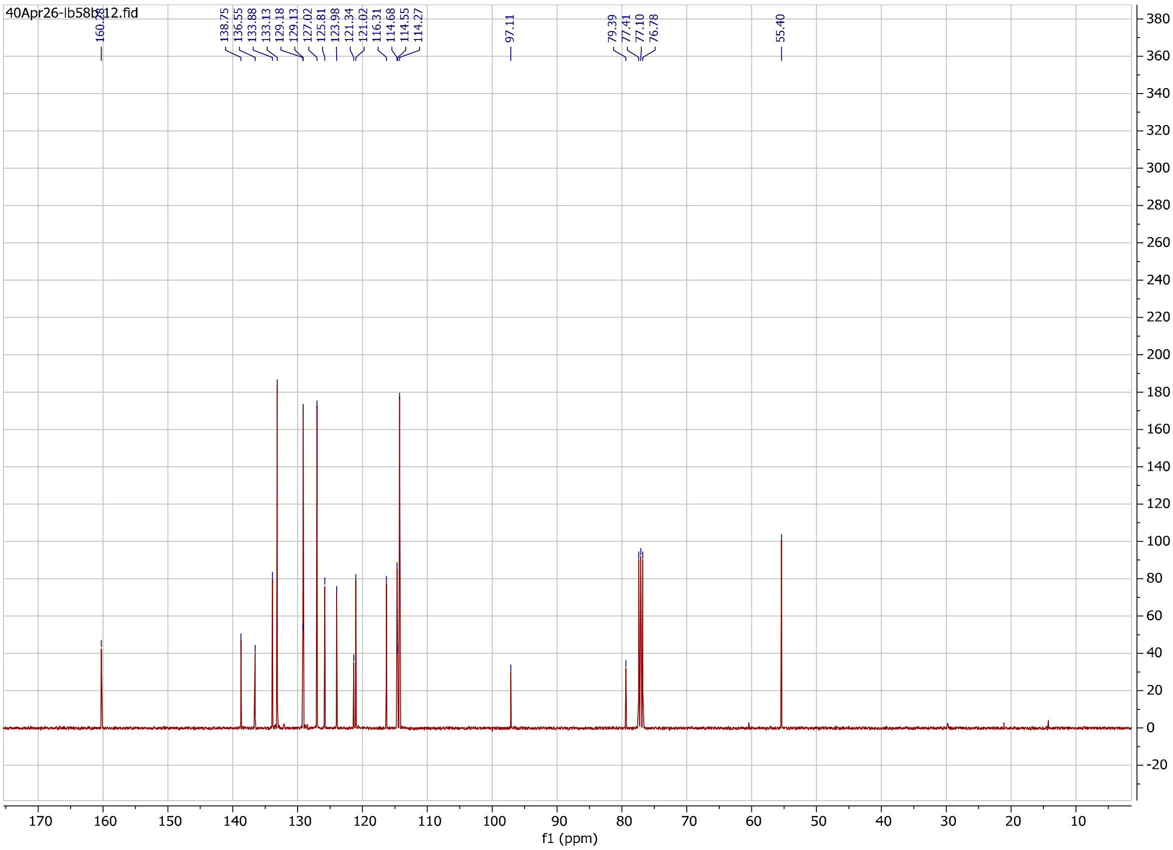

4-((1-Phenylsulfonyl)-1*H*-indol-2-yl)ethynyl)aniline 9

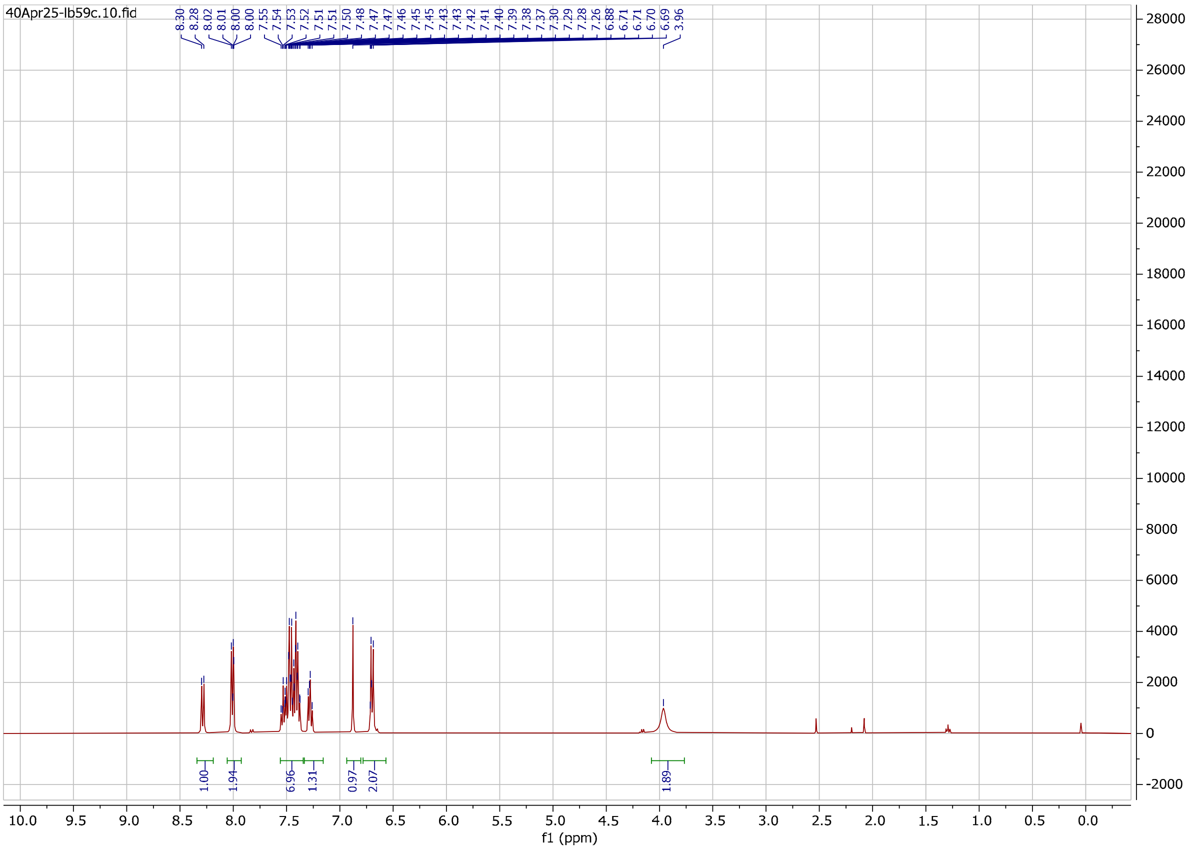

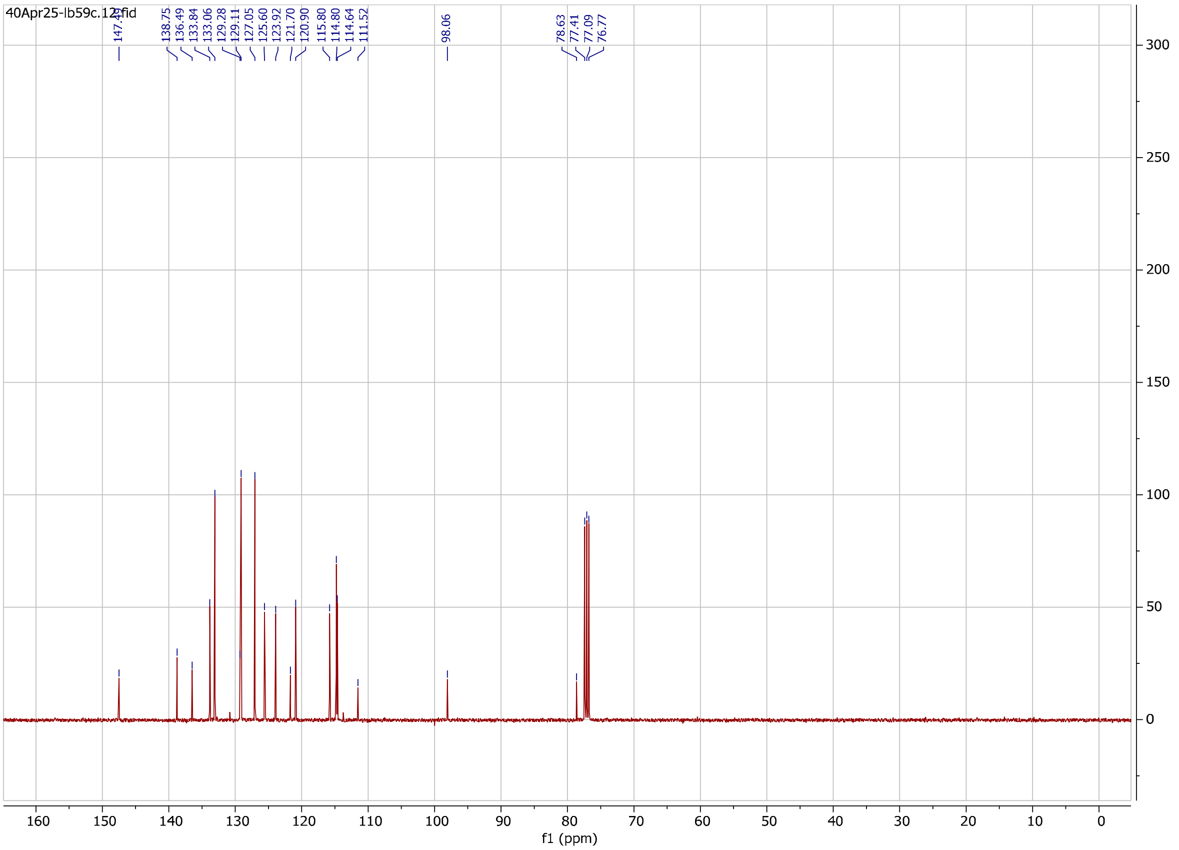

3-((1-(Phenylsulfonyl)-1*H*-indol-2-yl)ethynyl)aniline 10

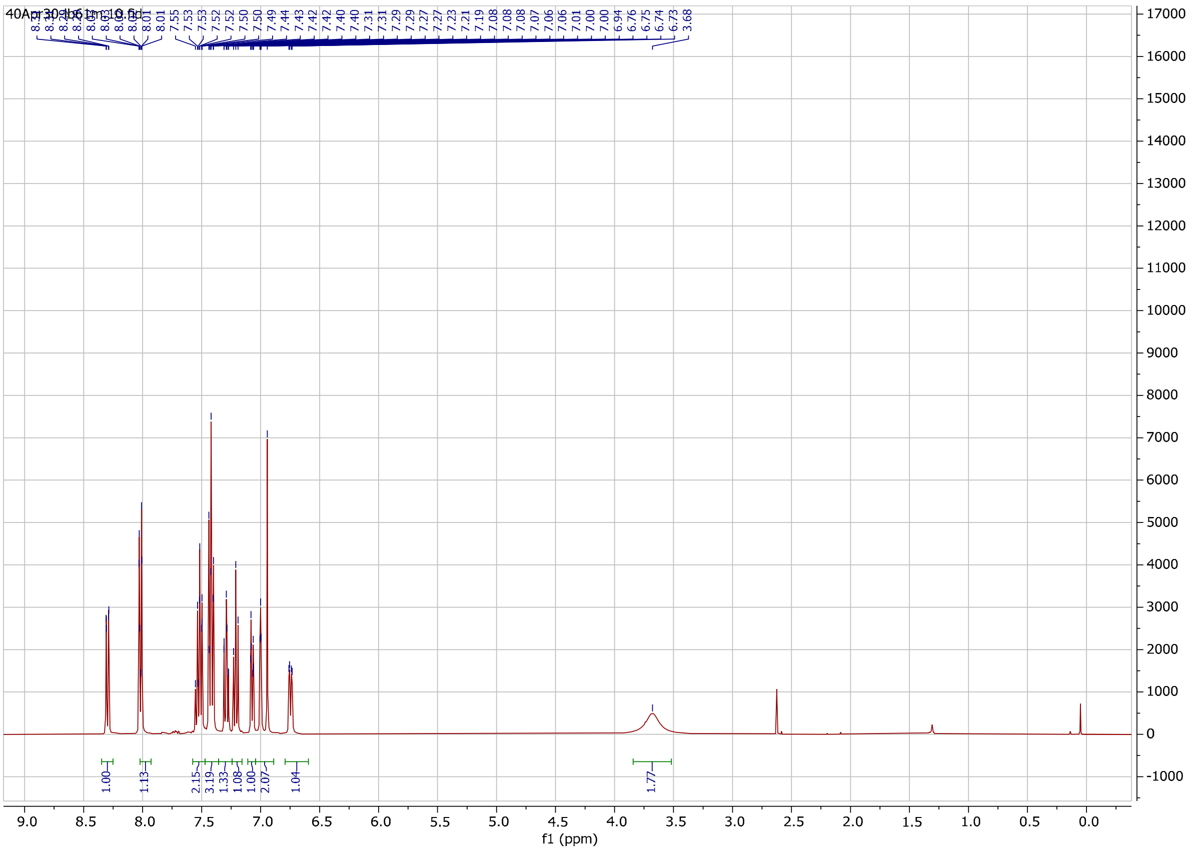

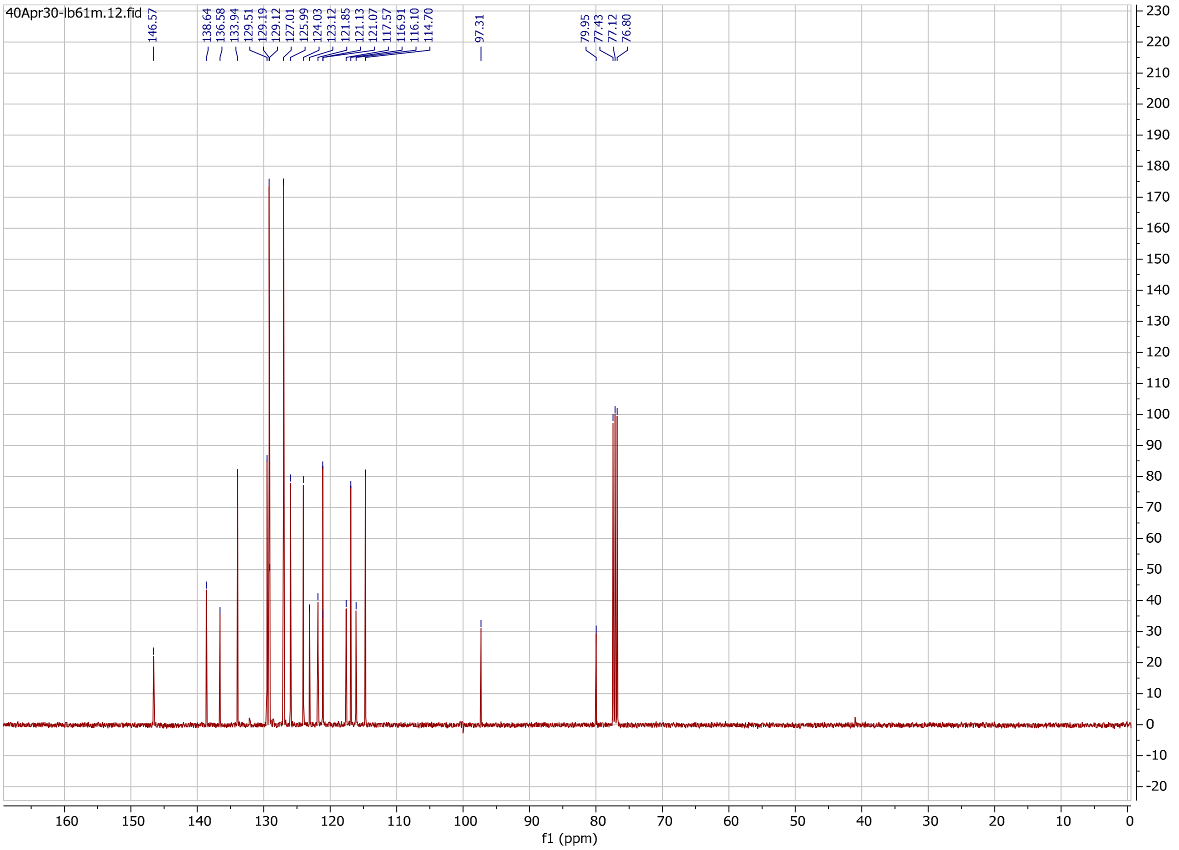

N-Methyl-4-((1-(phenylsulfonyl)-1*H*-indol-2-yl)ethynyl)aniline 11

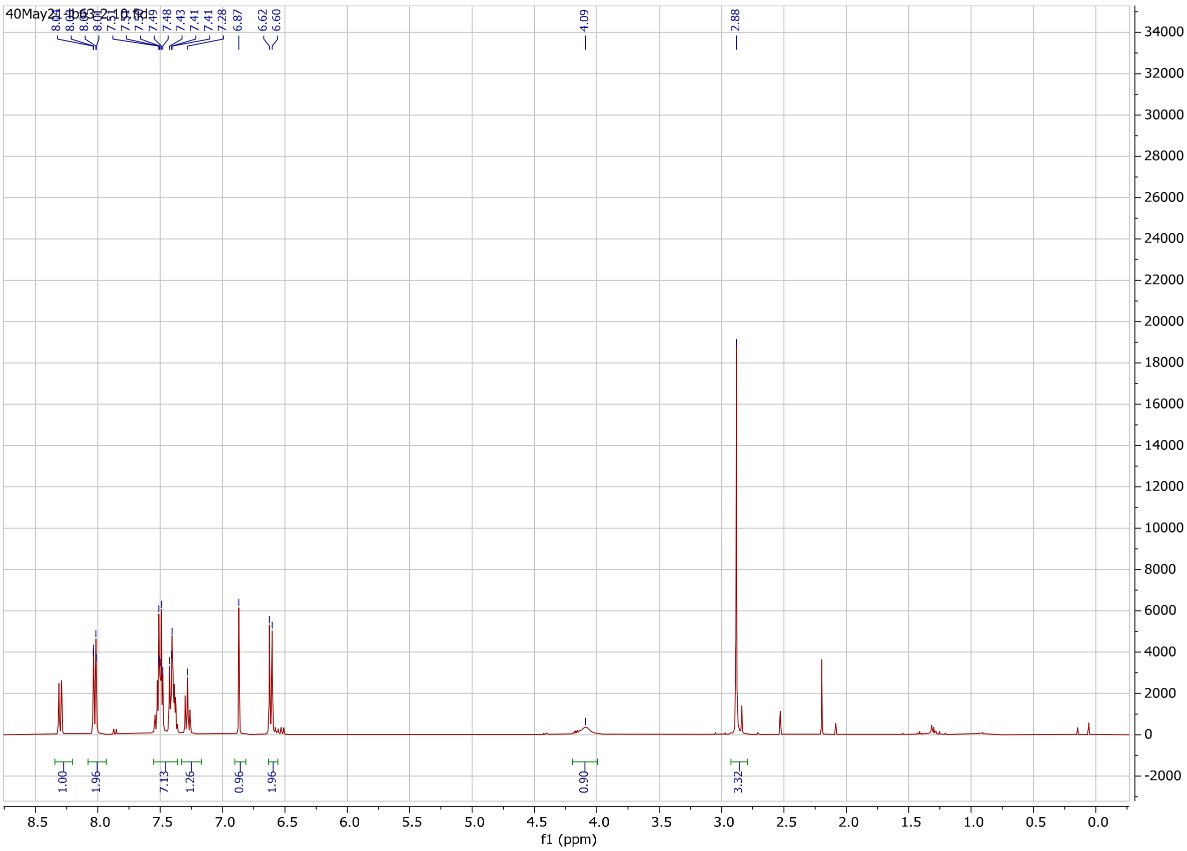

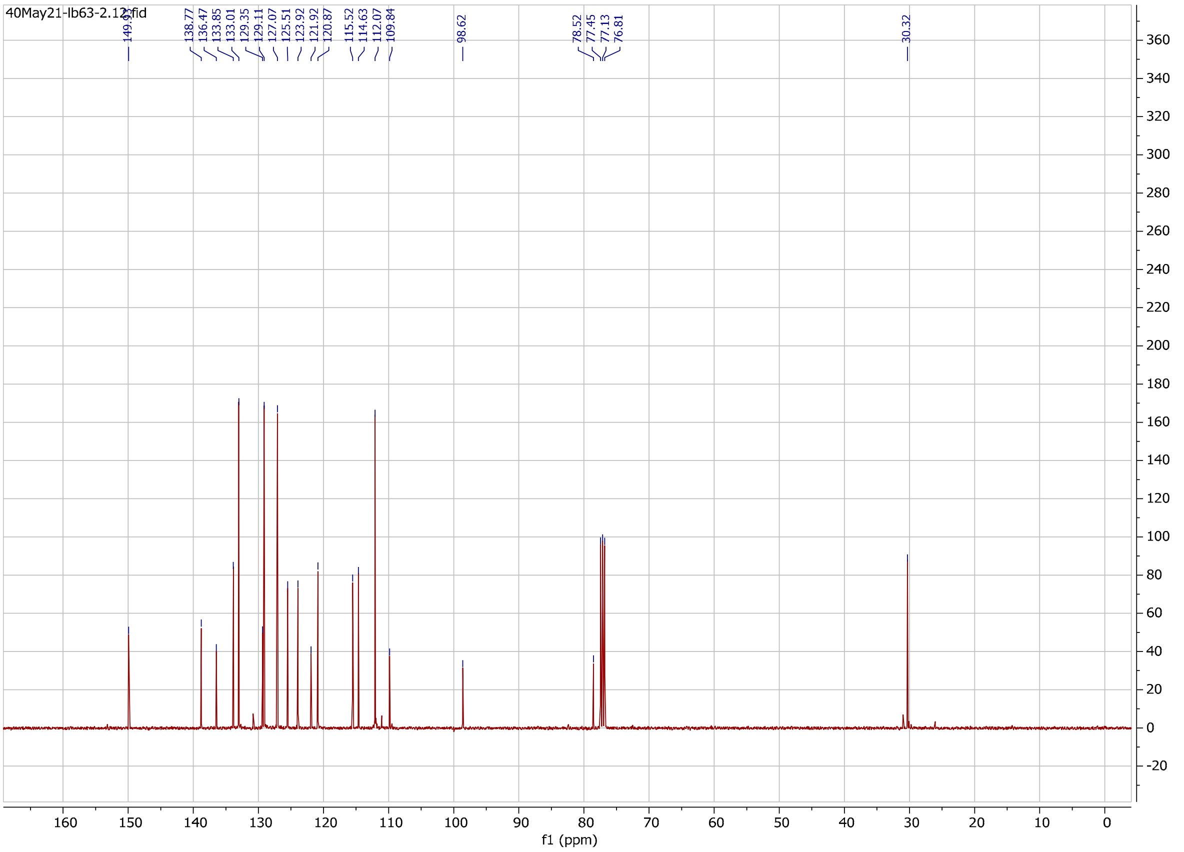

N,N-Dimethyl-4-((1-(phenylsulfonyl)-1*H*-indol-2-yl)ethynyl)aniline 12

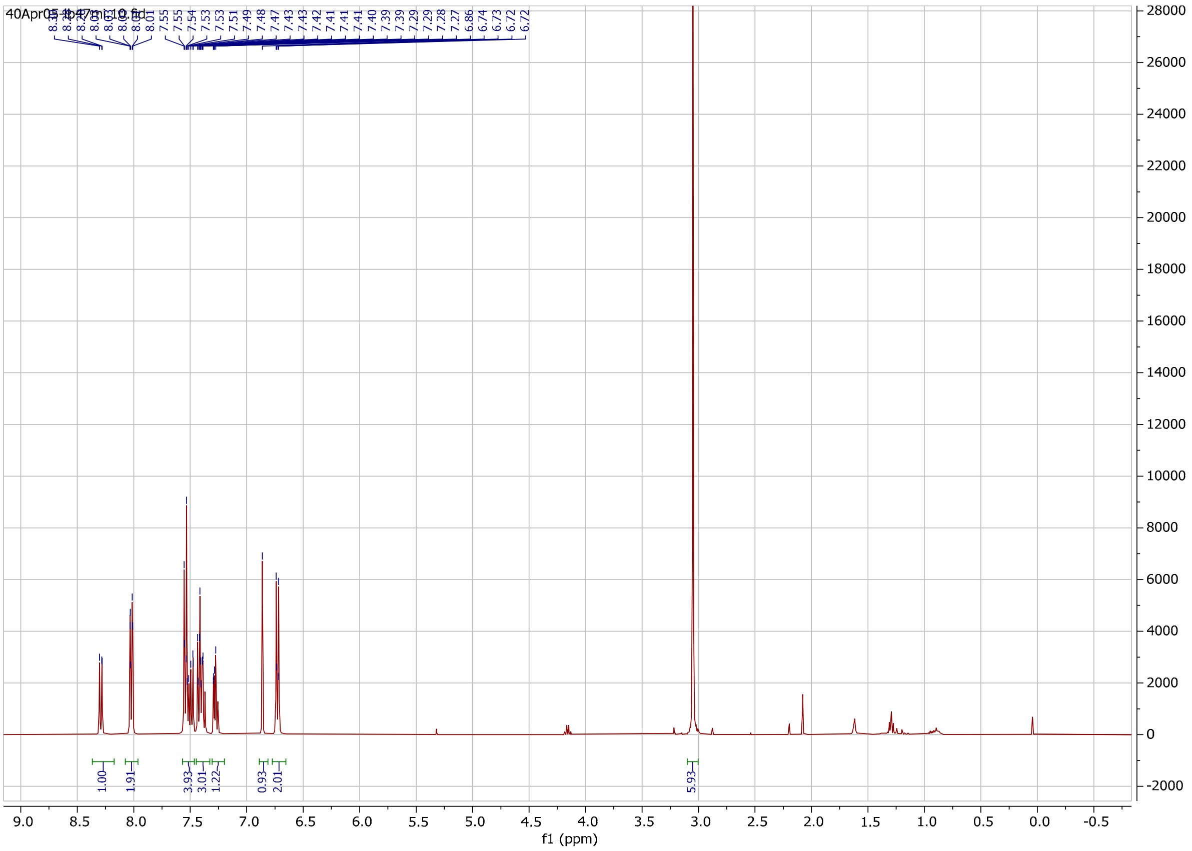

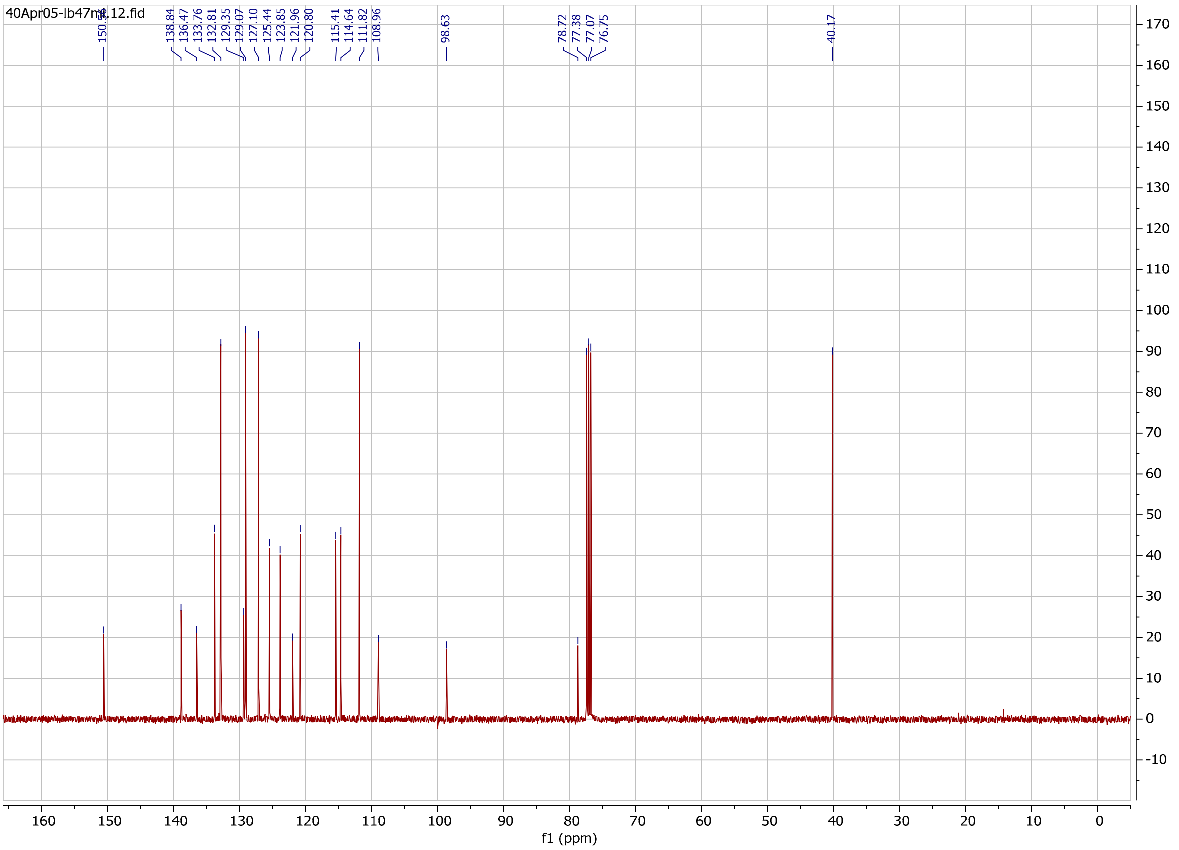

4-((5-Methoxy-1-(phenylsulfonyl)-1*H*-indol-2-yl)ethynyl)-*N*,*N*-dimethylaniline 13

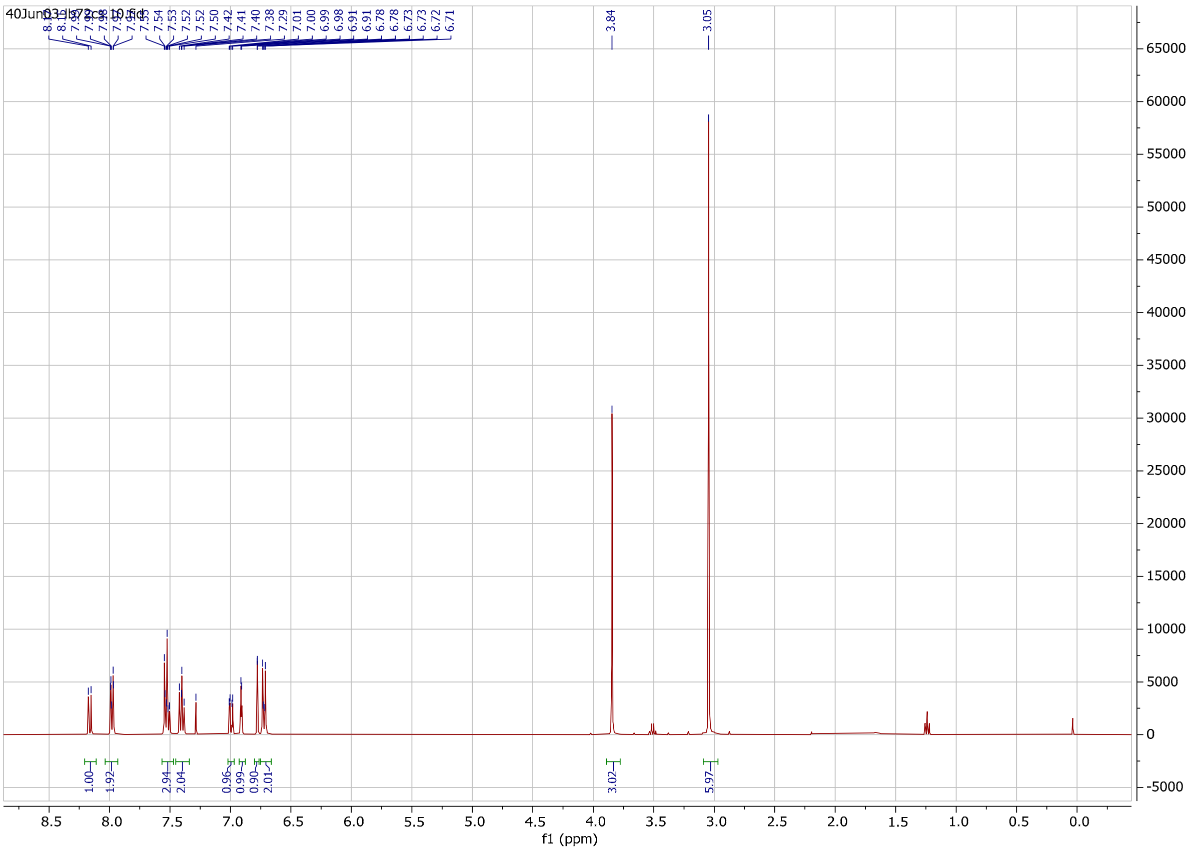

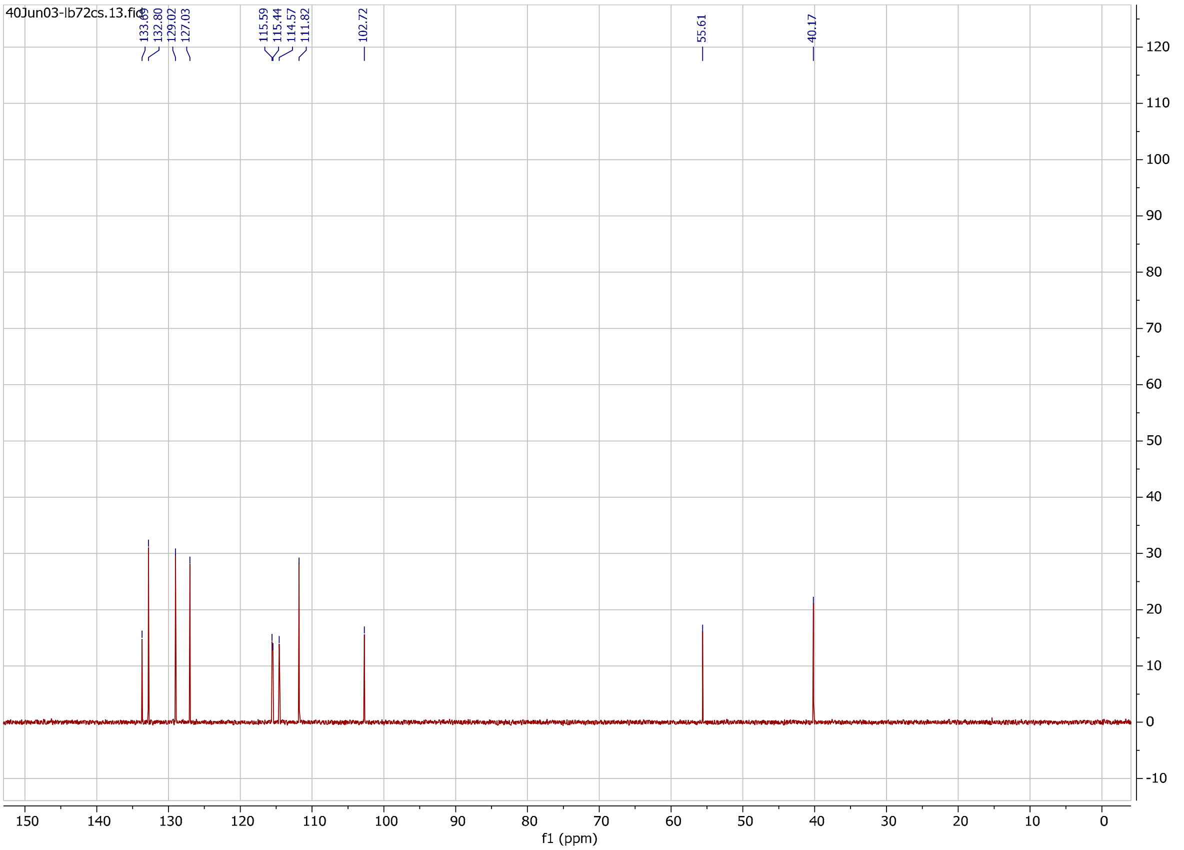

N,N-Dimethyl-4-((1-(phenylsulfonyl)-1*H*-pyrrolo[2,3-*c*]pyridin-2-yl)ethynyl)aniline 21

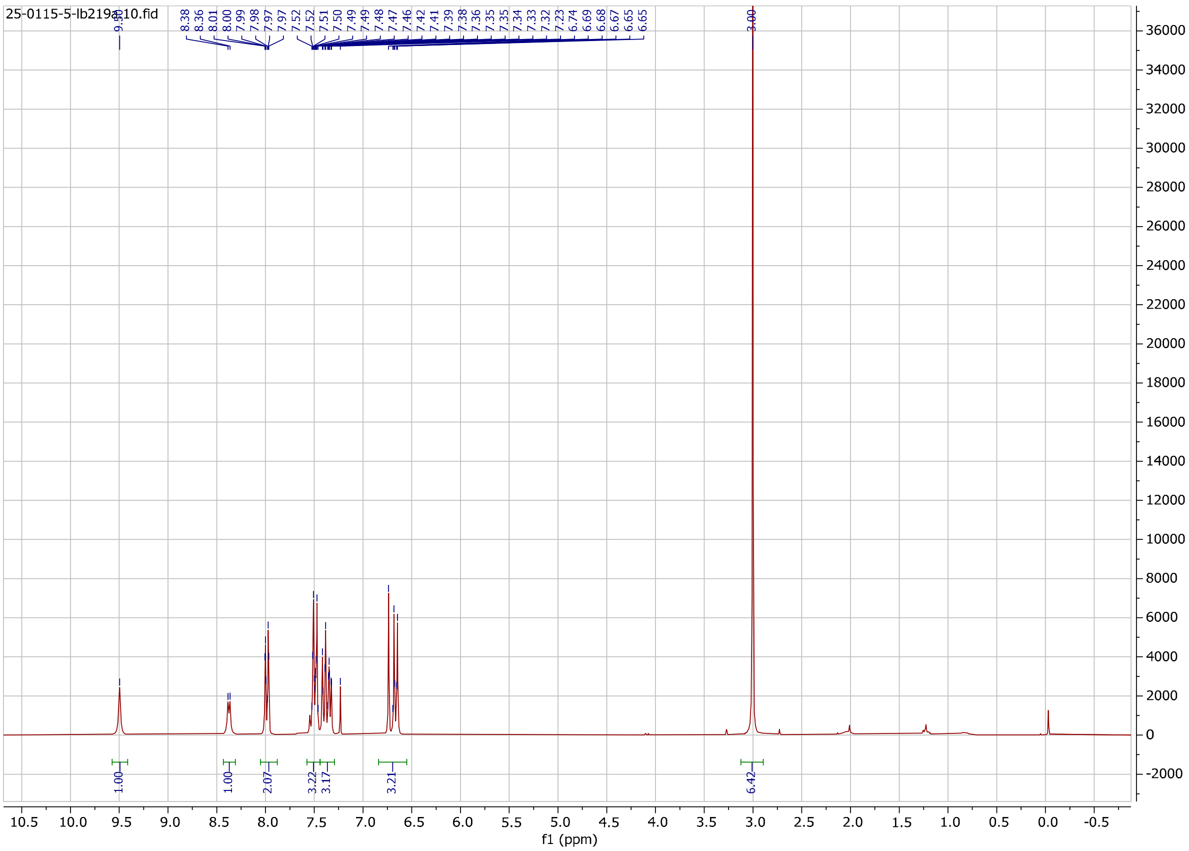

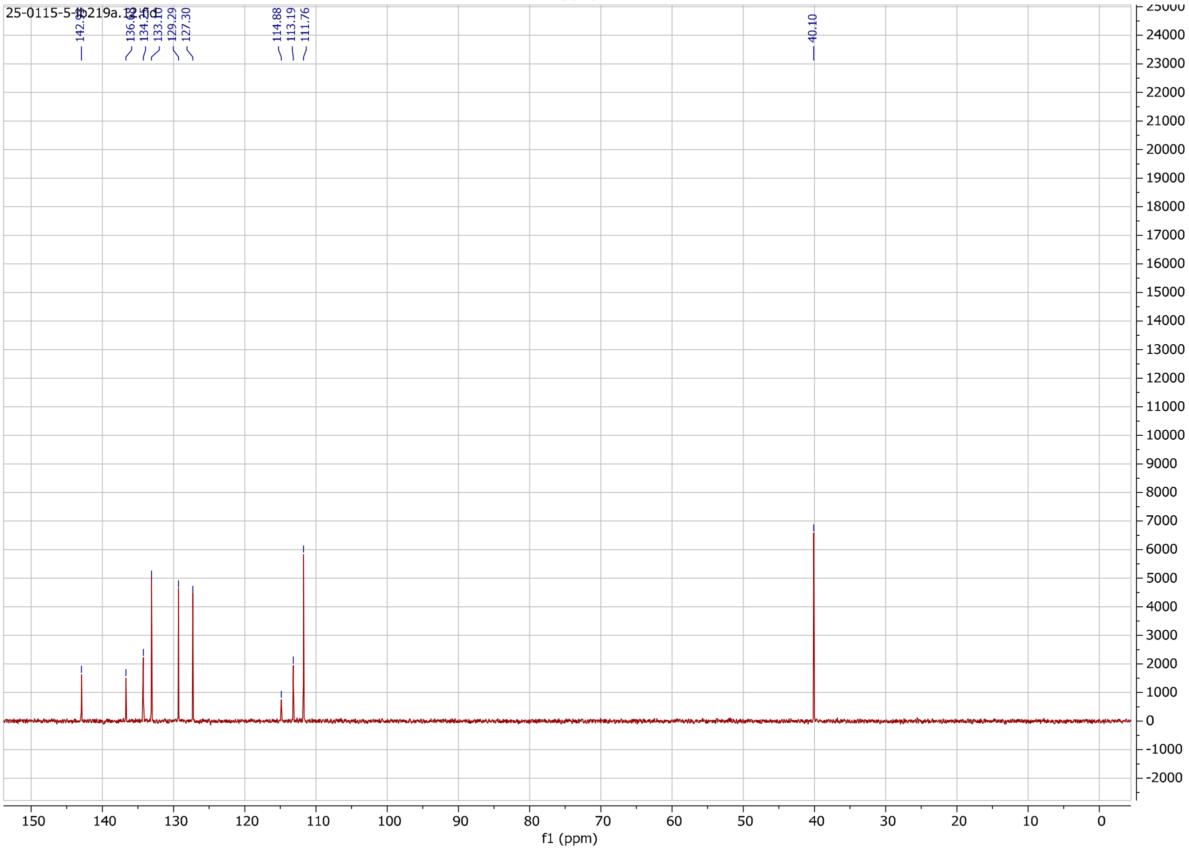

N,N-Dimethyl-4-((1-(phenylsulfonyl)-1*H*-pyrrolo[2,3-*b*]pyridin-2-yl)ethynyl)aniline 22

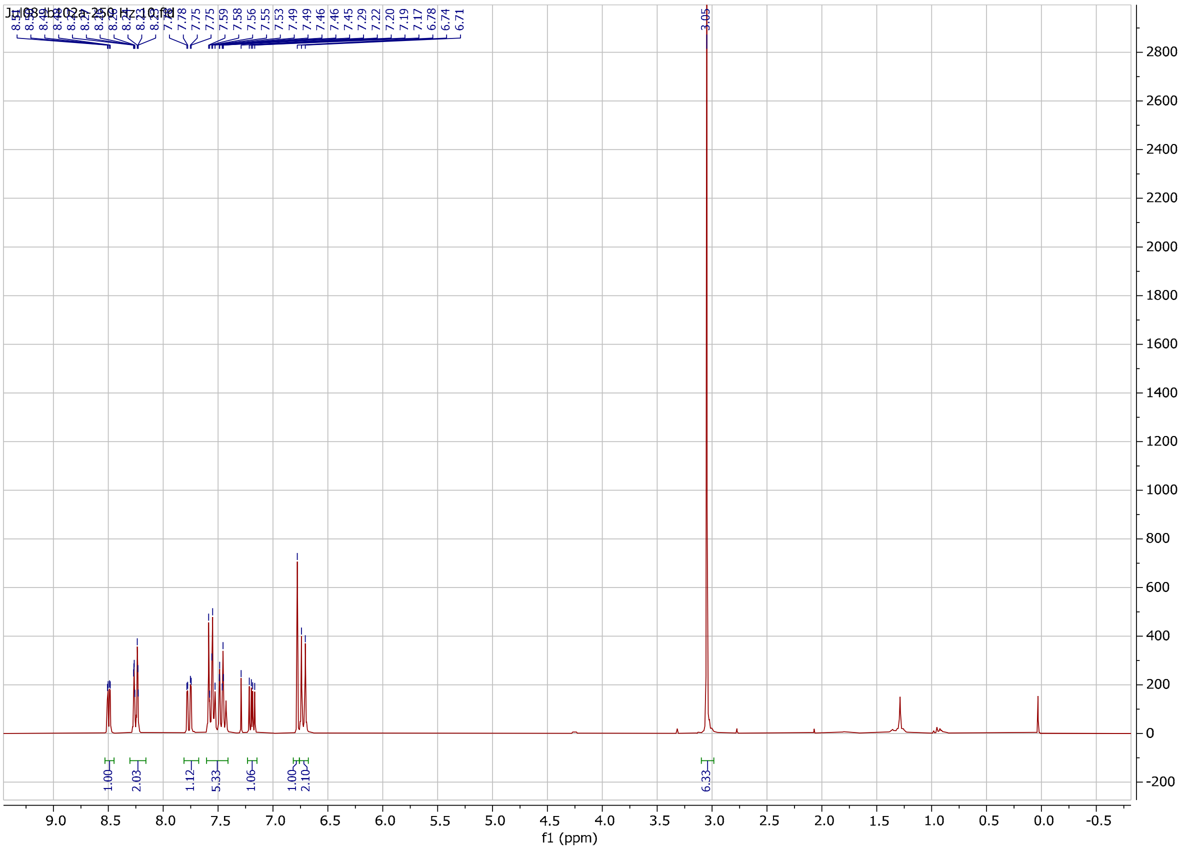

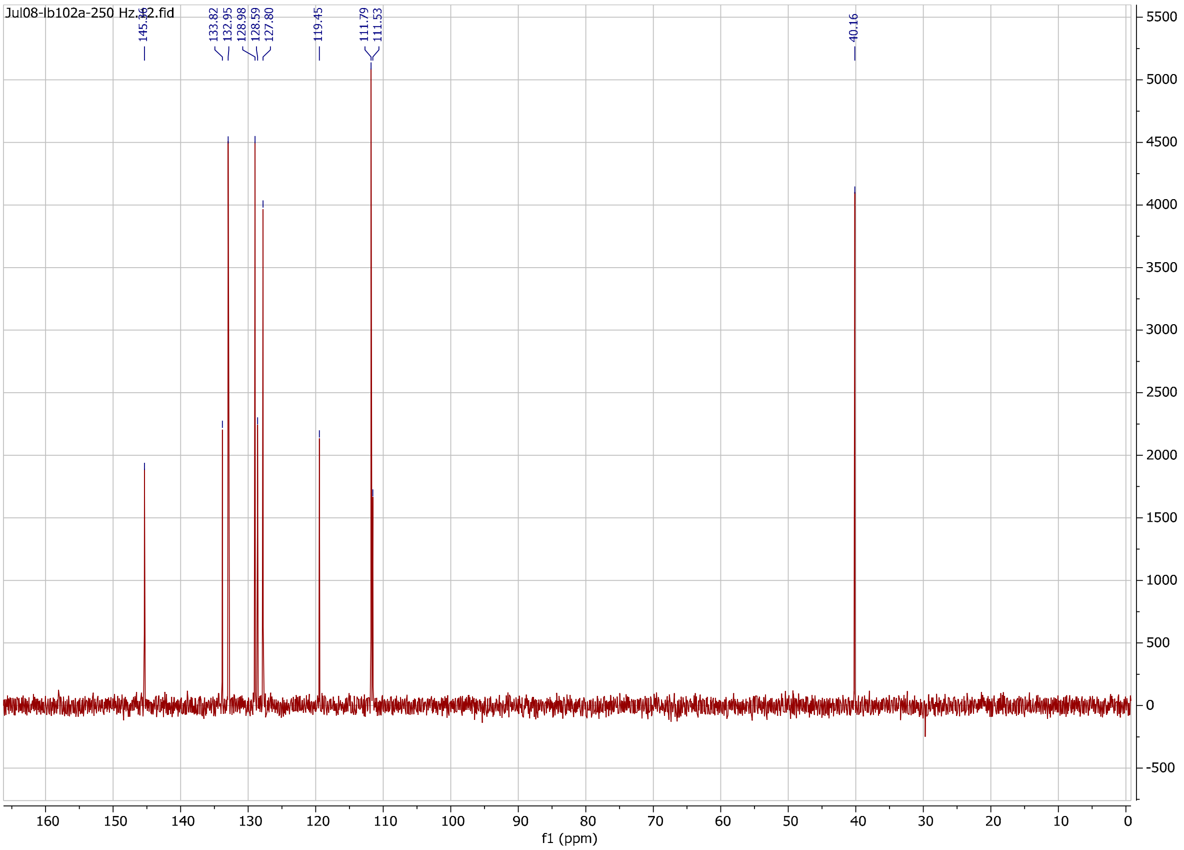

tert-Butyl 2-iodo-1*H*-pyrrolo[3,2-*c*]pyridine-1-carboxylate 24

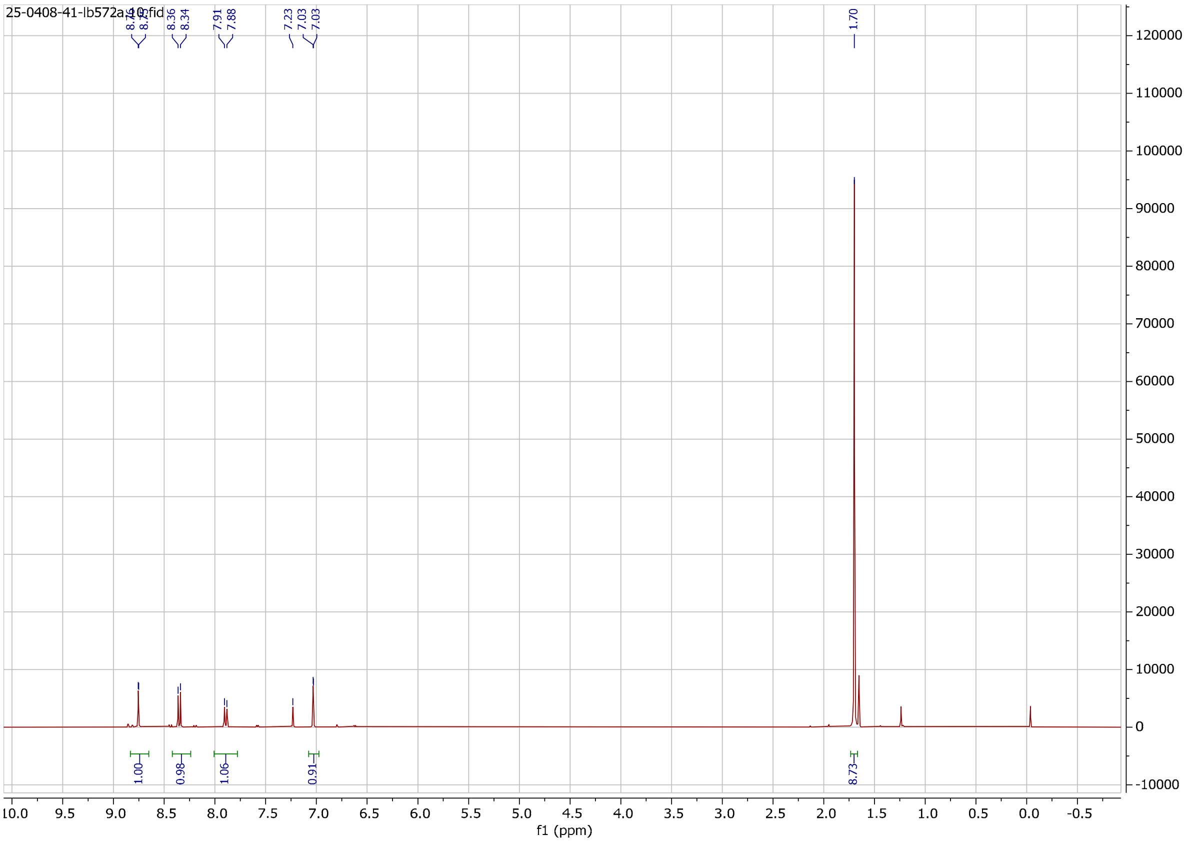

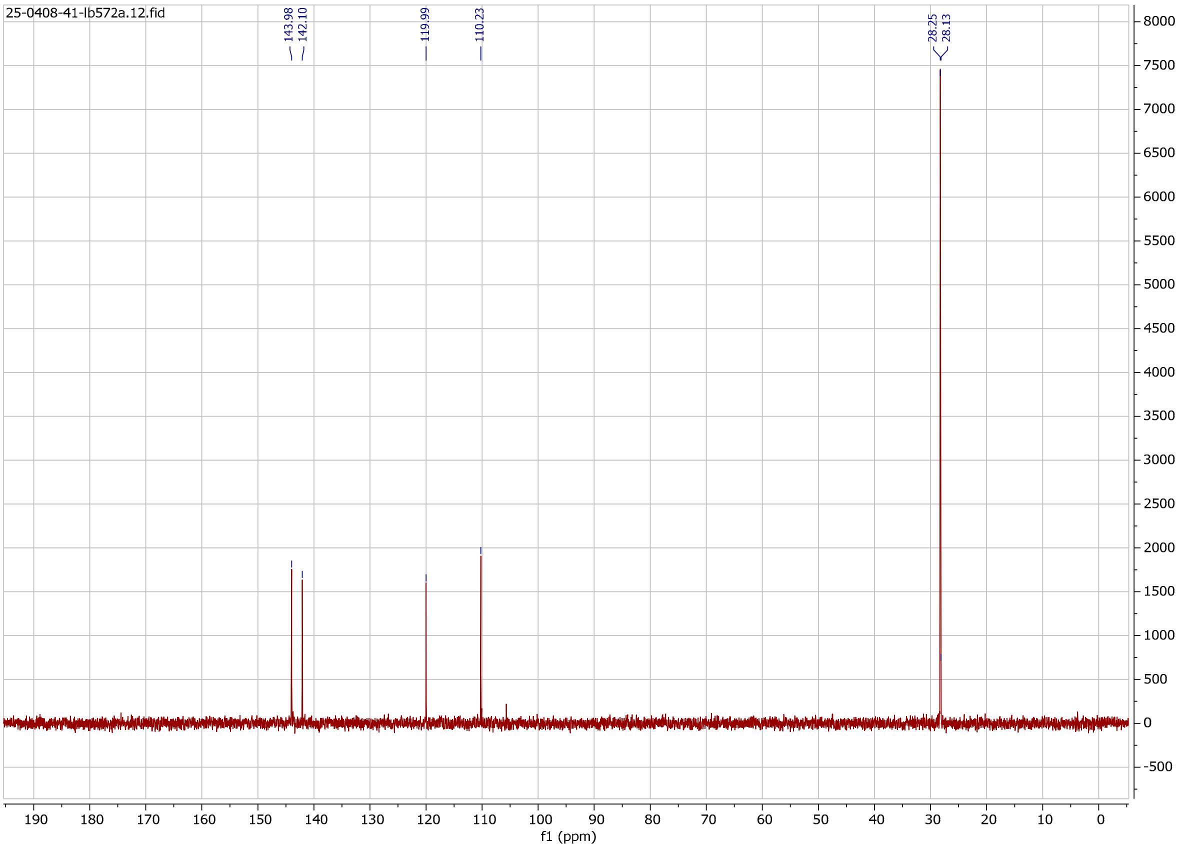

tert-Butyl 2-((4-(dimethylamino)phenyl)ethynyl)-1_H_-pyrrolo[3,2-_c_]pyridine-1-carboxylate 25

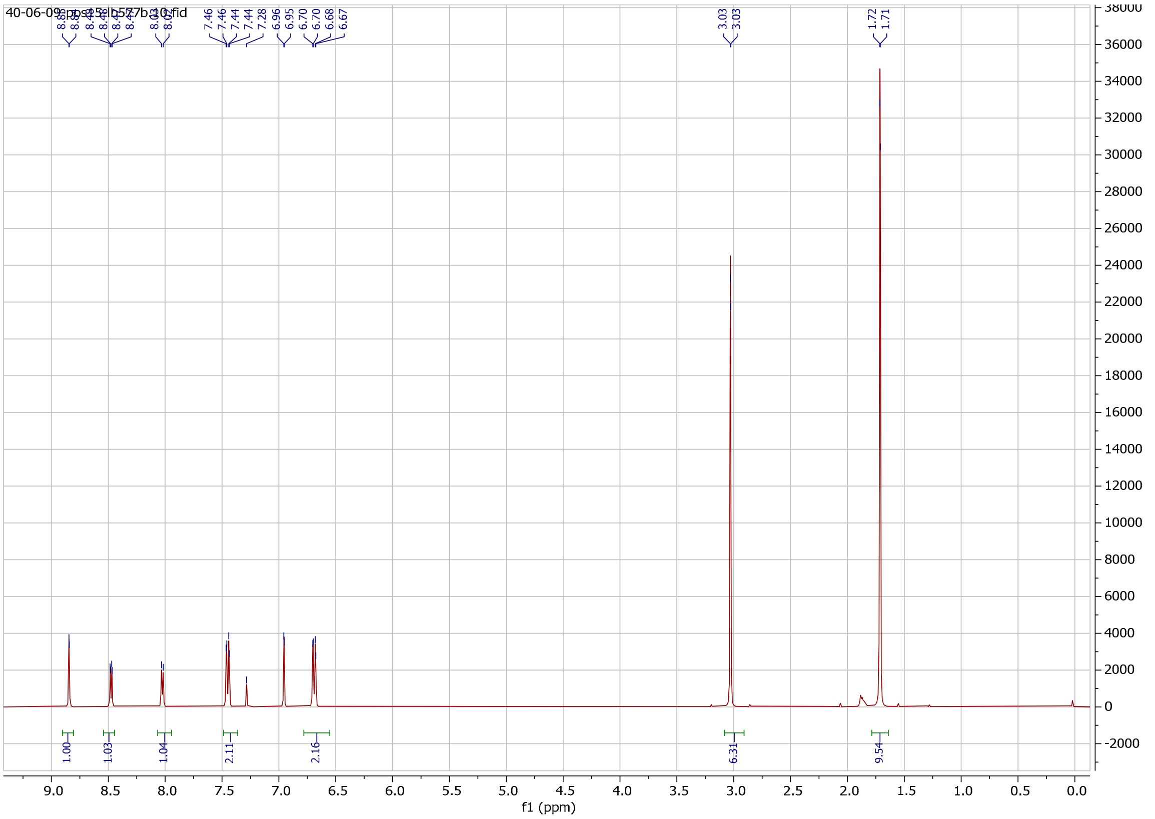

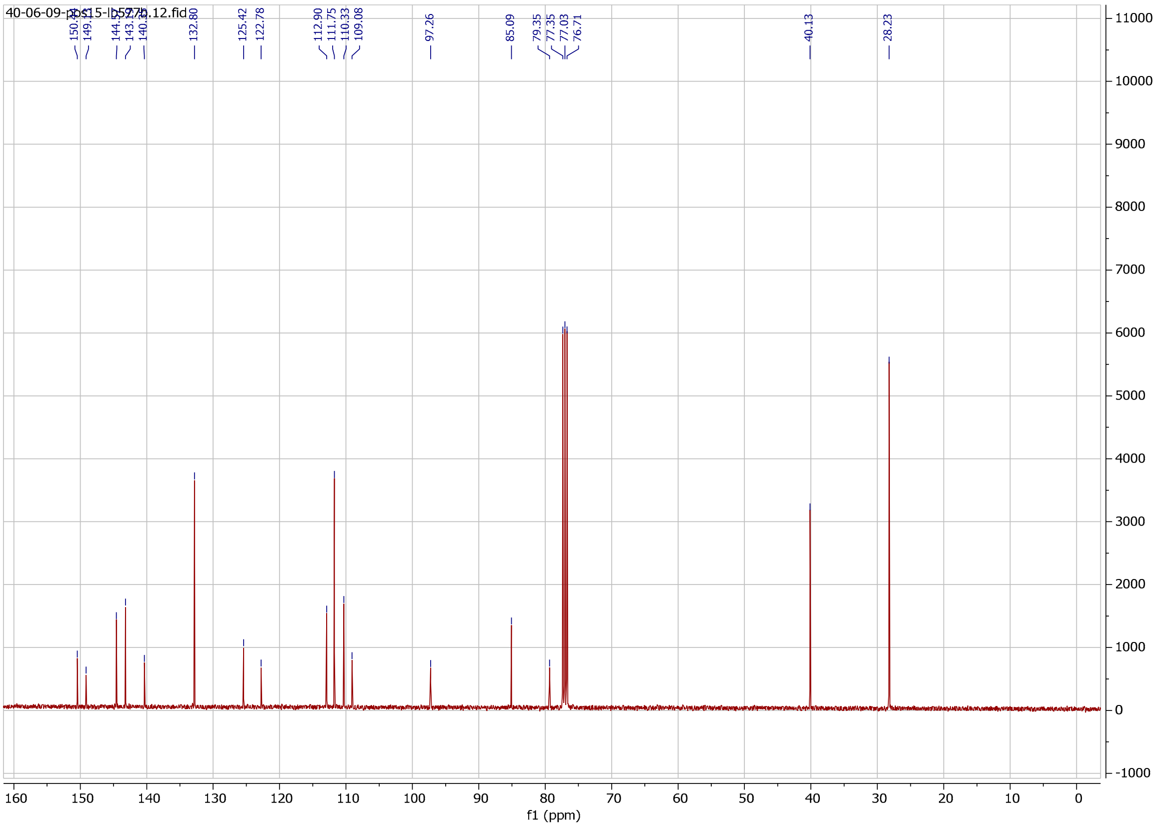

tert-Butyl 2-iodo-1*H*-pyrrolo[2,3-*b*]pyridine-1-carboxylate 27

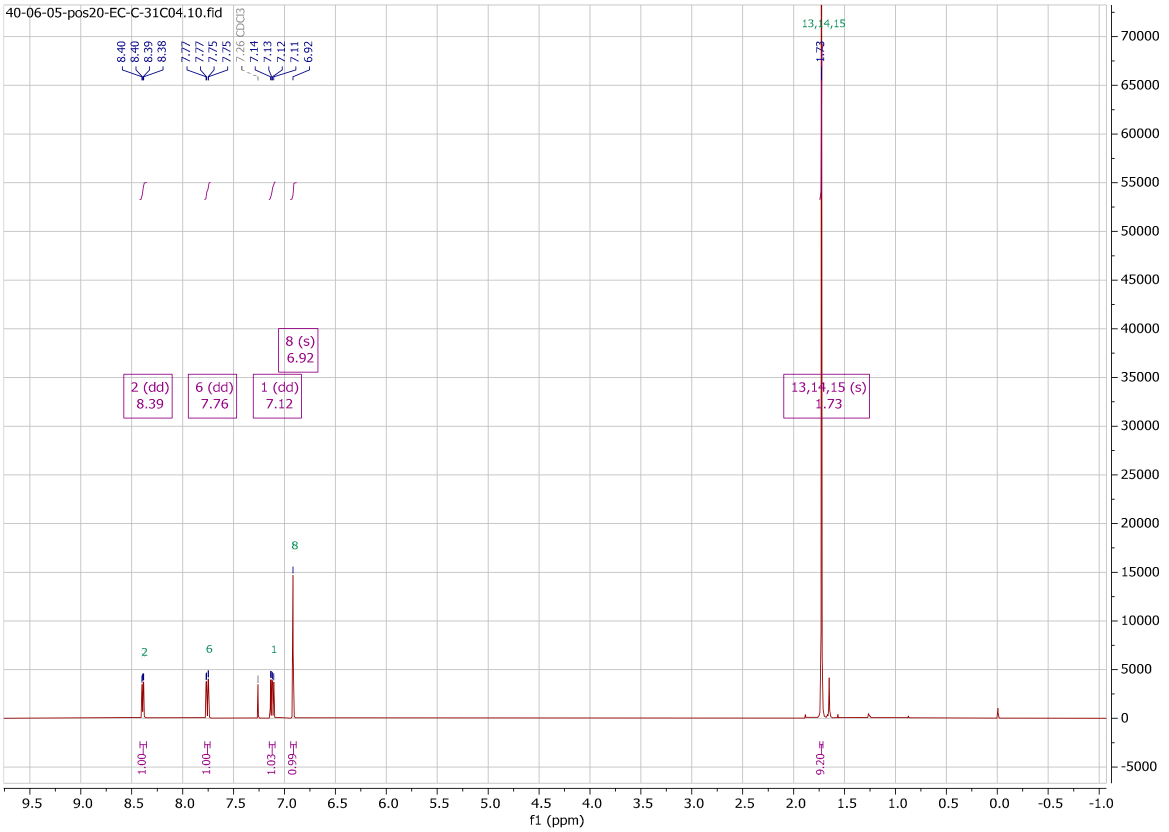

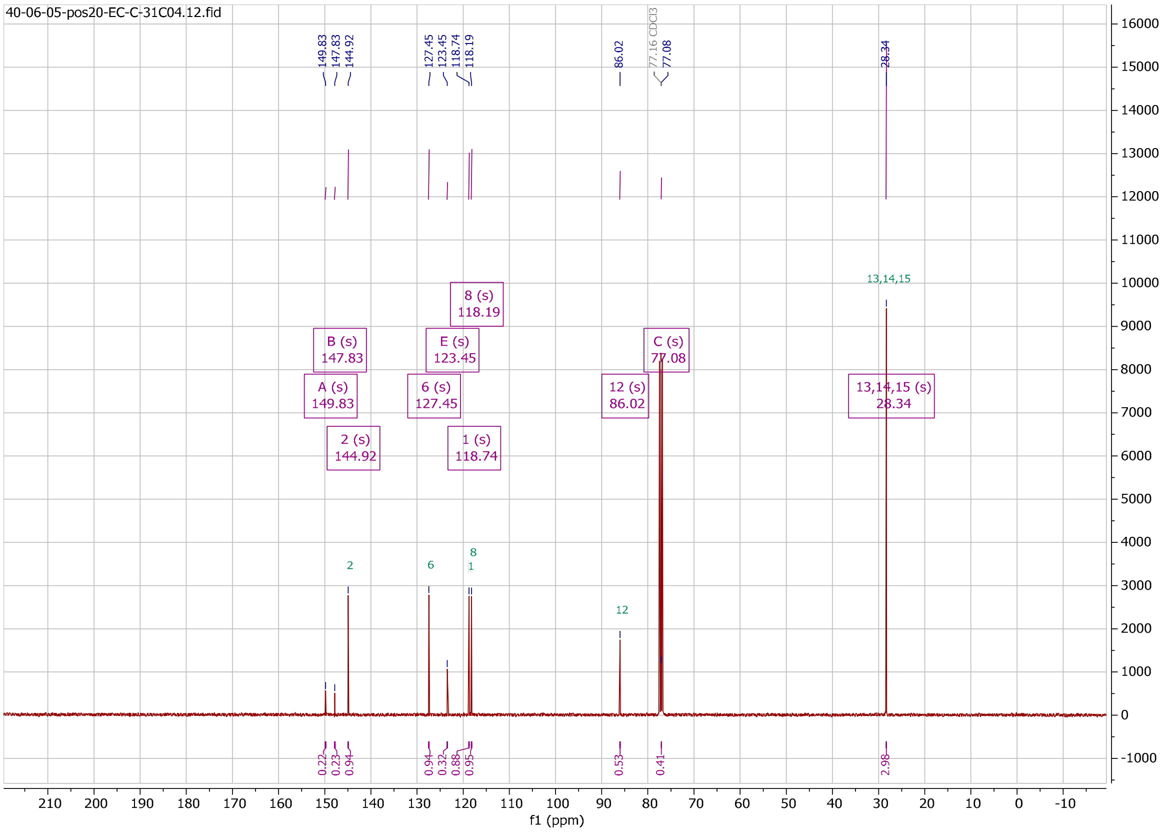

tert-Butyl 2-((4-(methylamino)phenyl)ethynyl)-1*H*-pyrrolo[2,3-*b*]pyridine-1-carboxylate 28

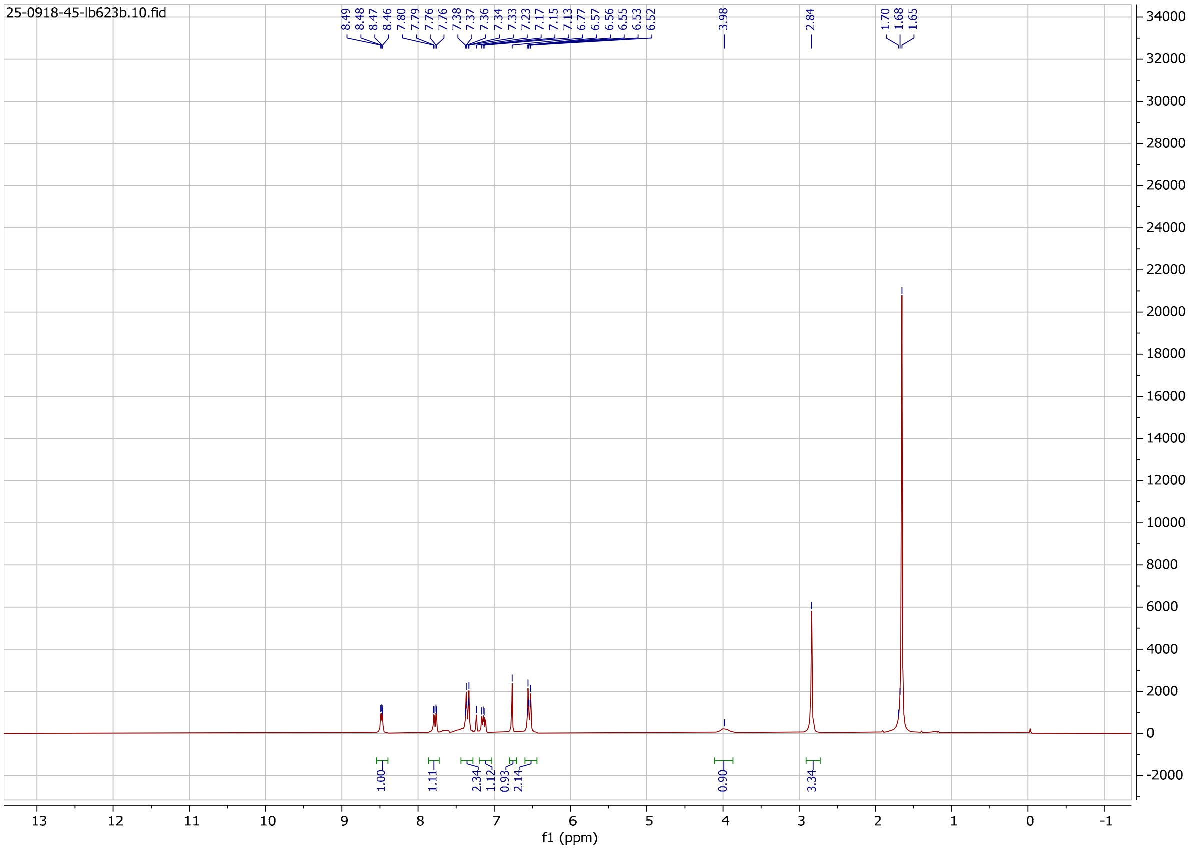

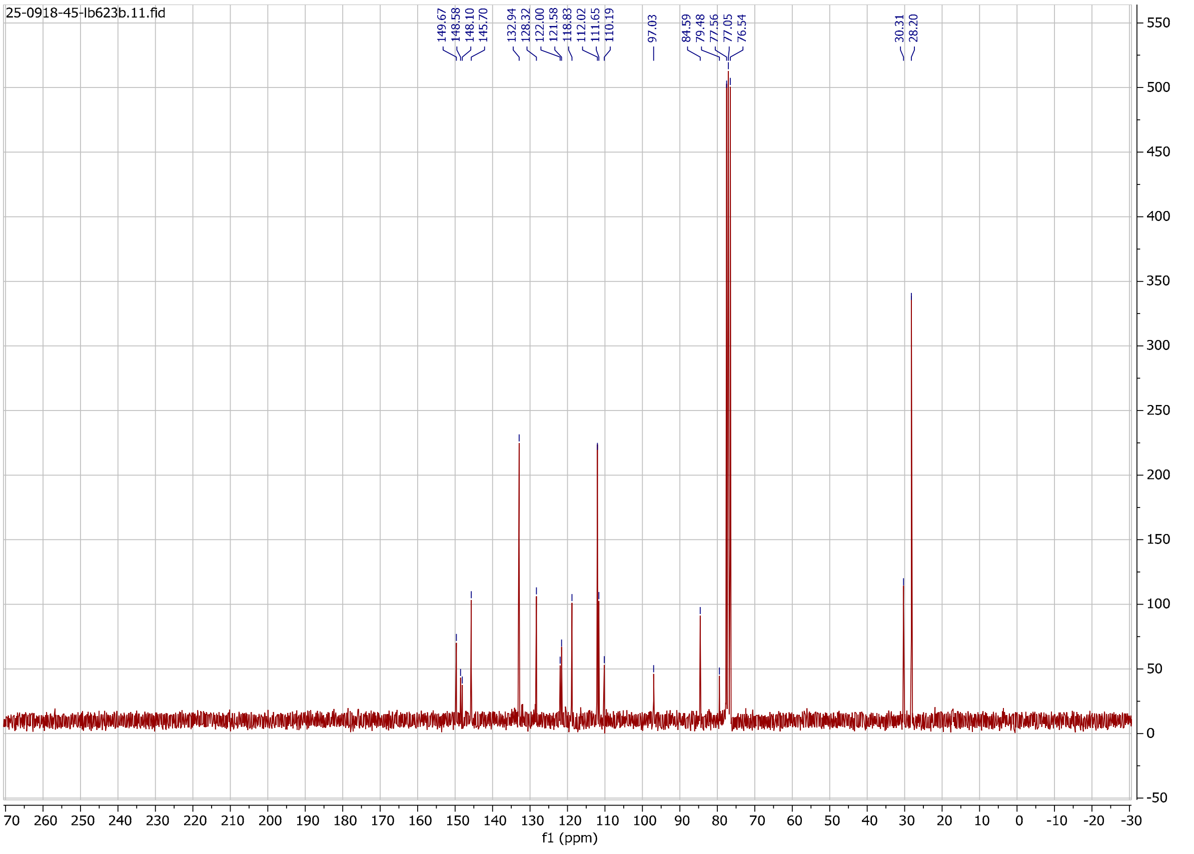

tert-Butyl 2-((4-((2-hydroxyethyl)(methyl)amino)phenyl)ethynyl)-1*H*-pyrrolo[2,3-*b*]pyridine-1-carboxylate 29

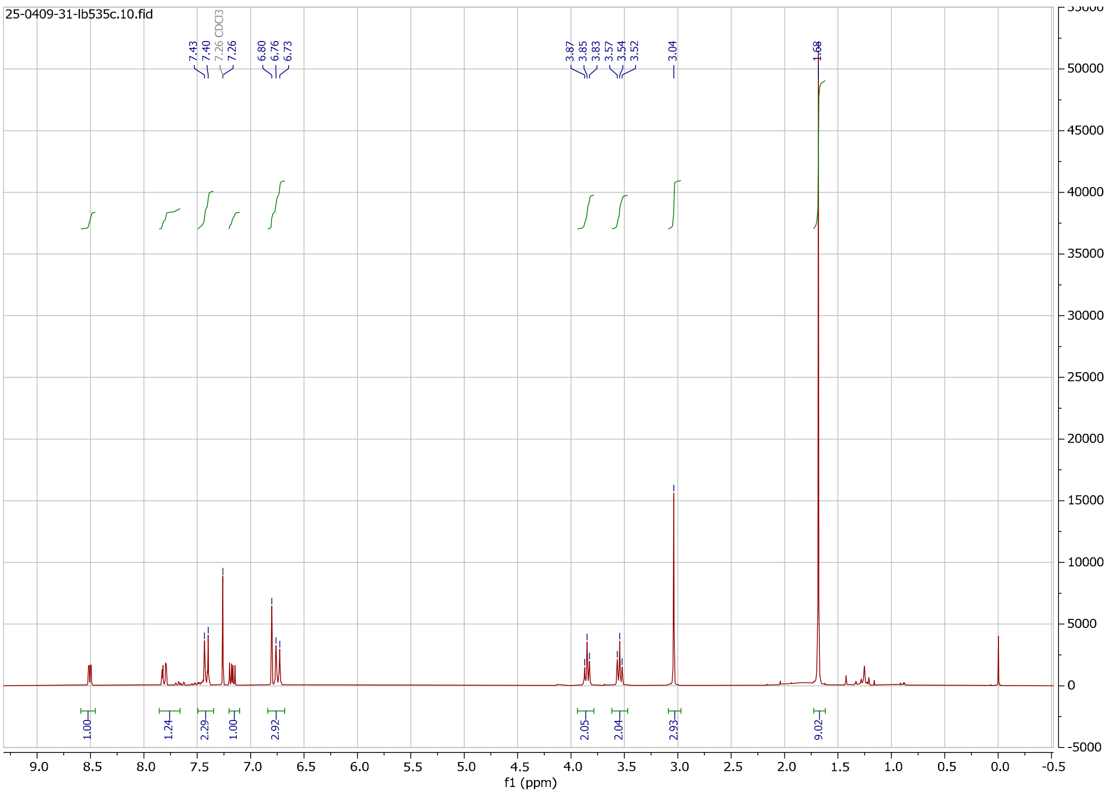

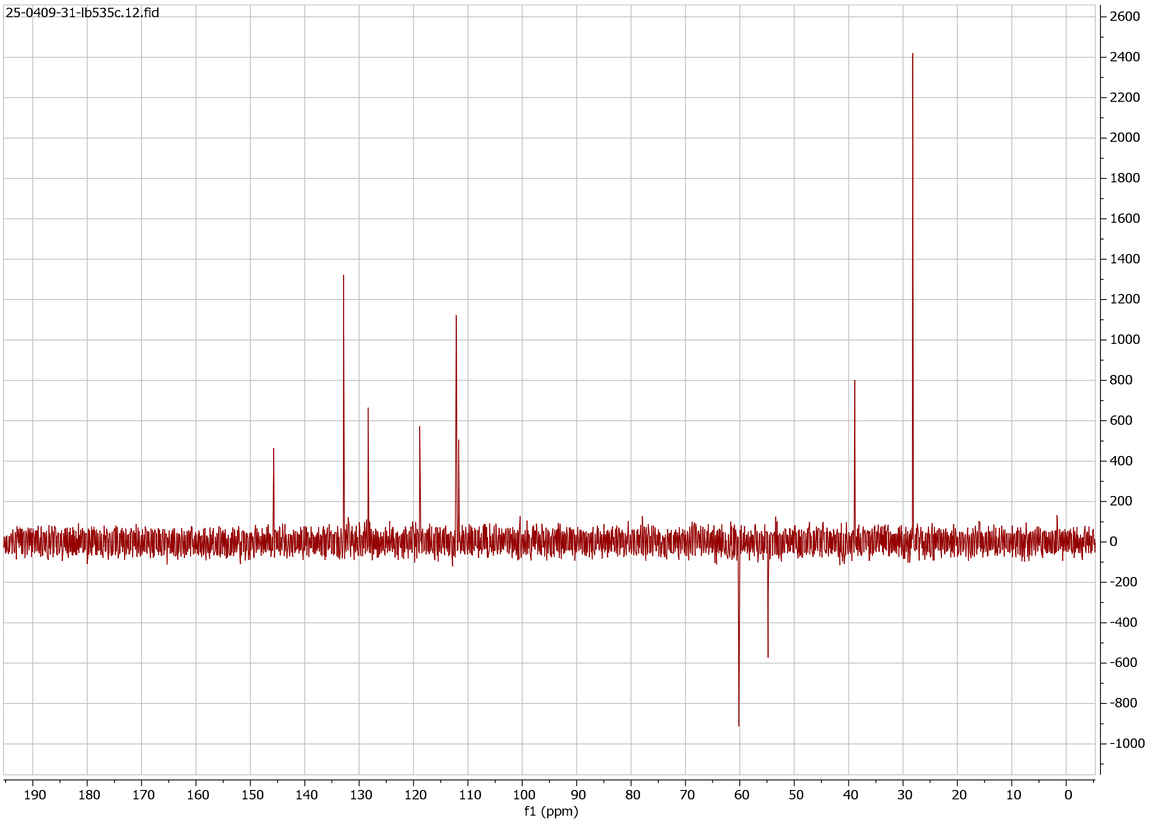

2-(Phenylethynyl)-1*H*-indole 31 [28]

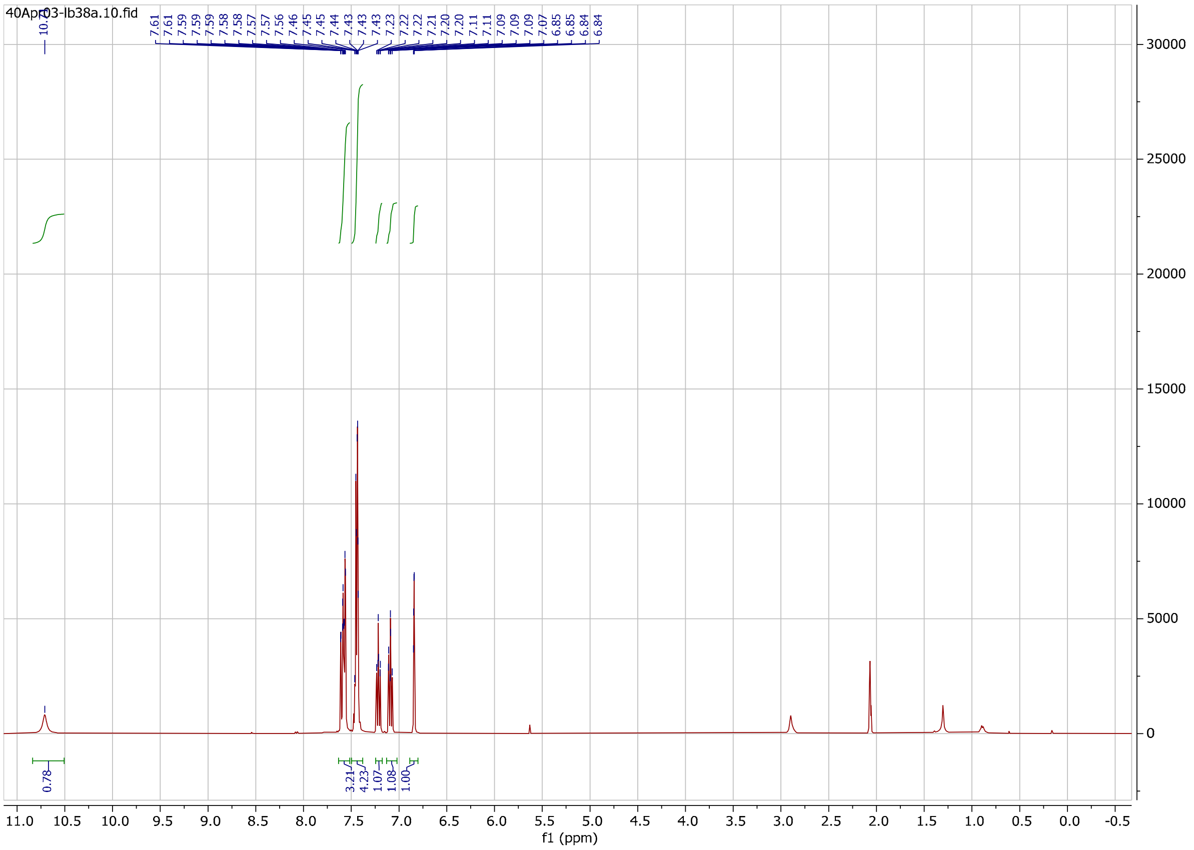

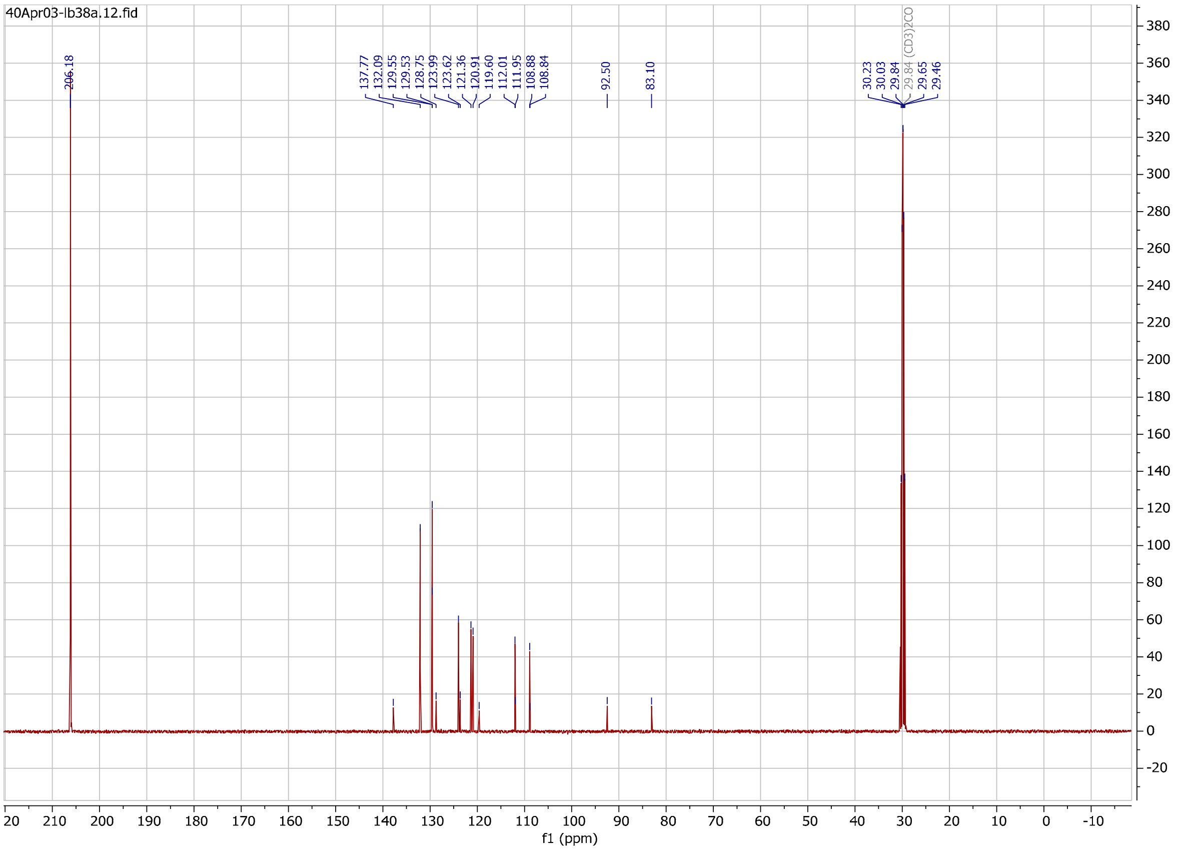

2-(*p*-Tolylethynyl)-1*H*-indole 32 [24]

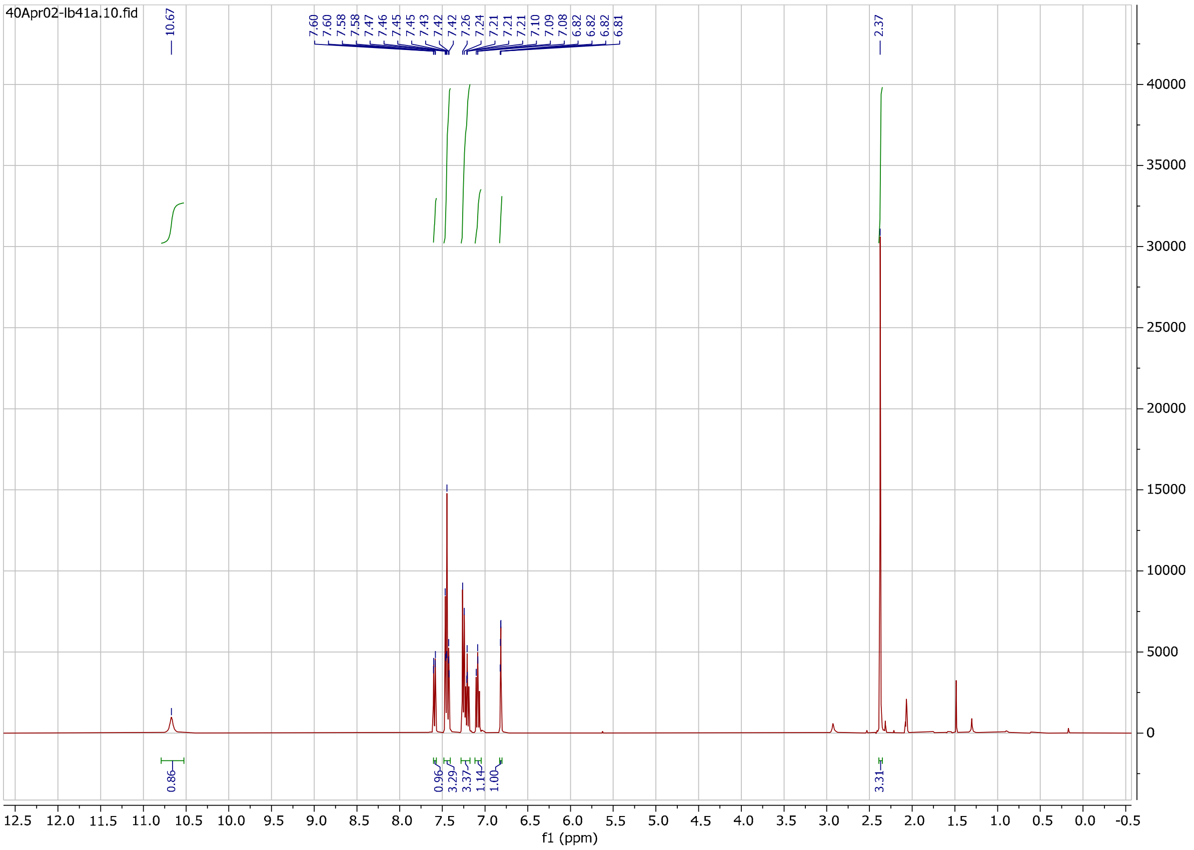

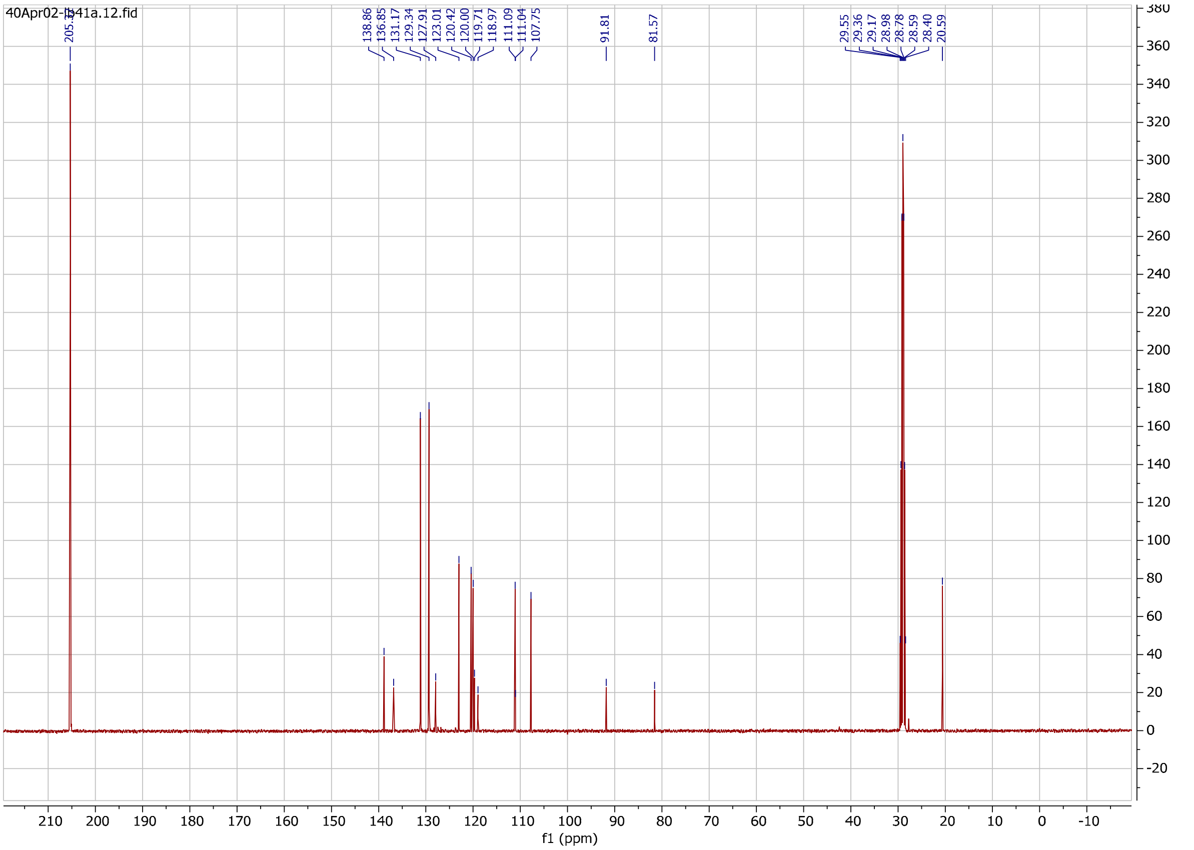

2-((4-Ethylphenyl)ethynyl)-1*H*-indole 33

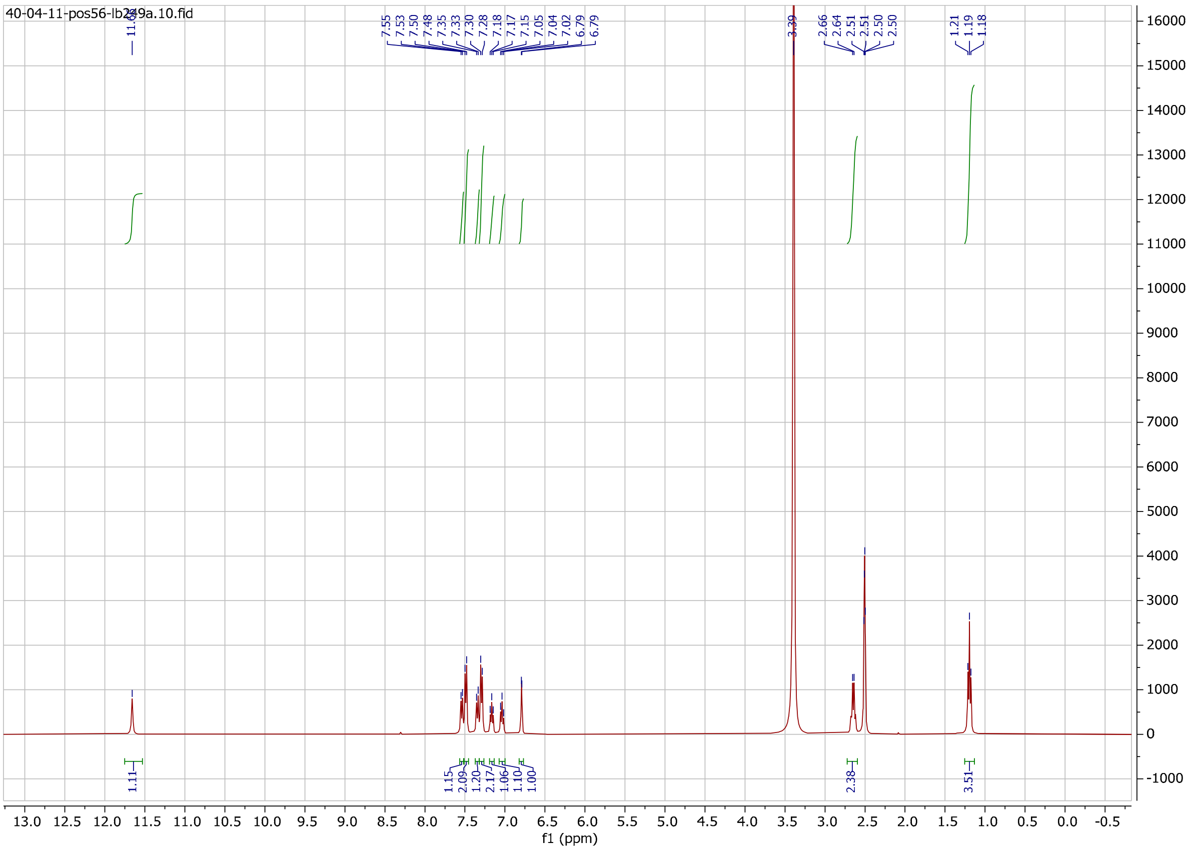

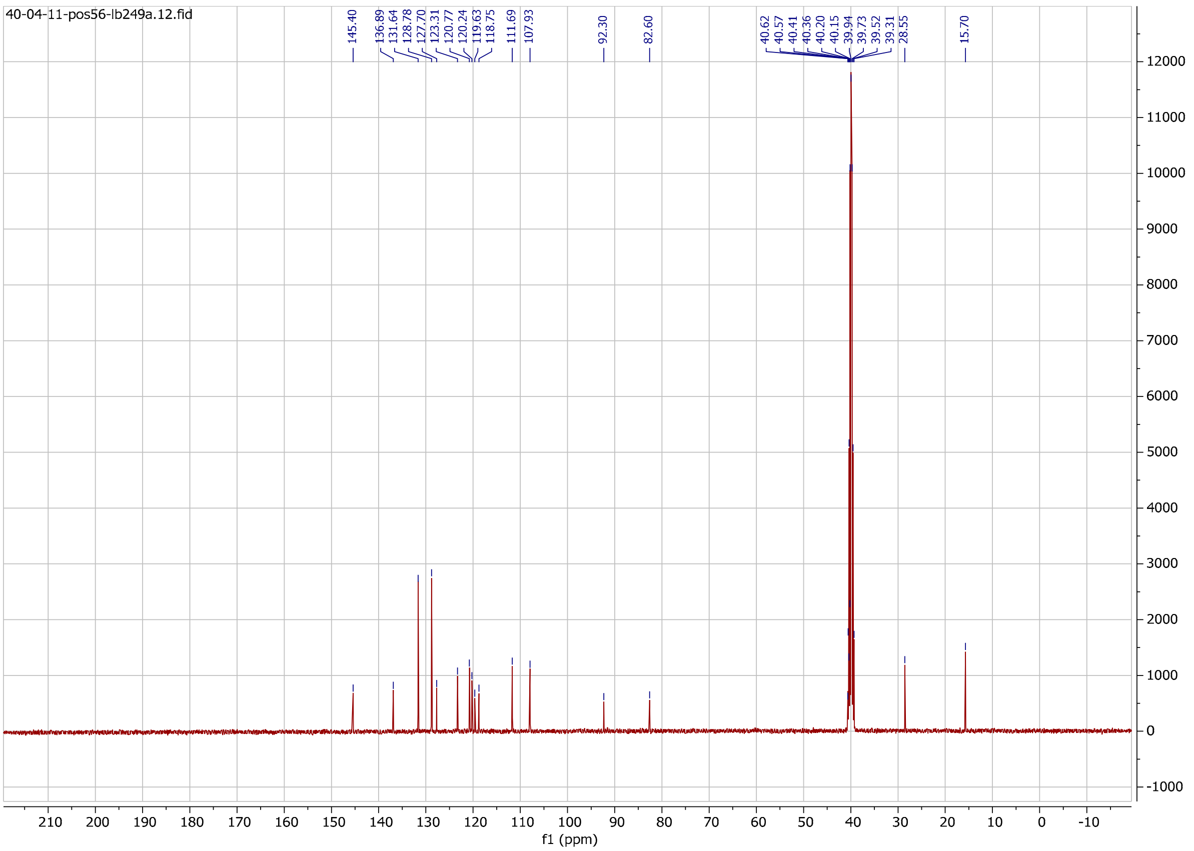

2-((4-Methoxyphenyl)ethynyl)-1*H*-indole 34 [29]

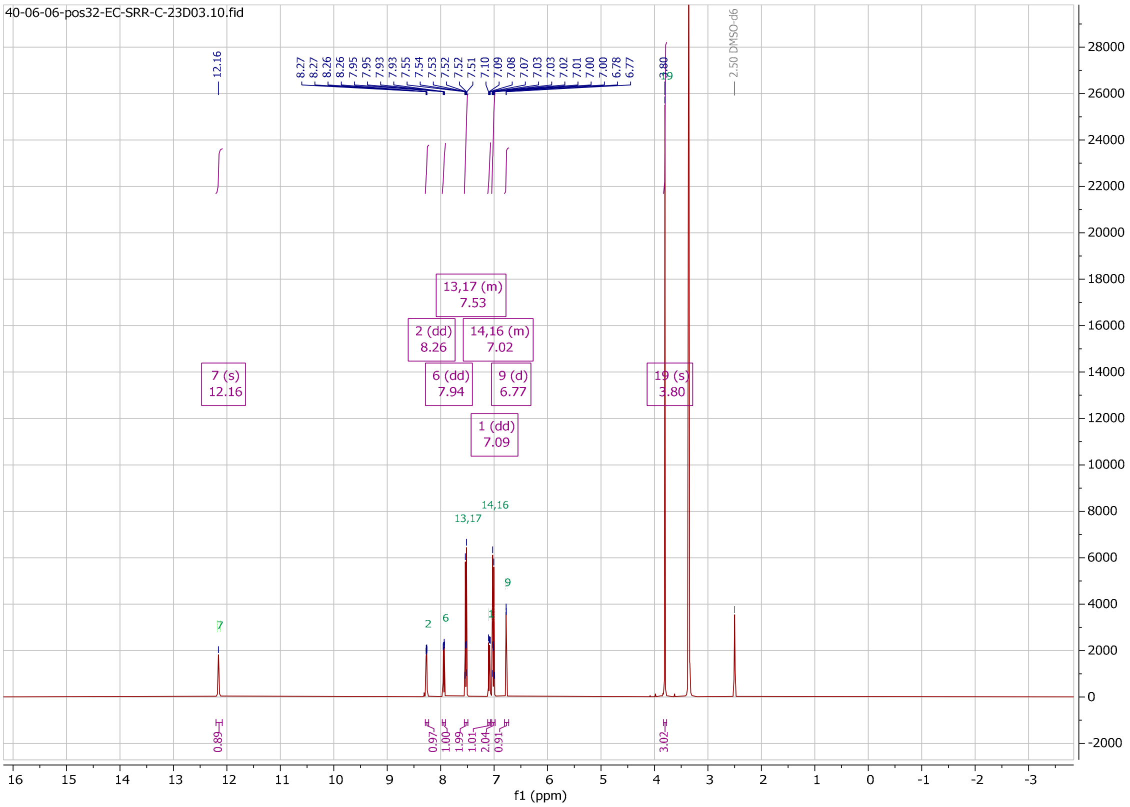

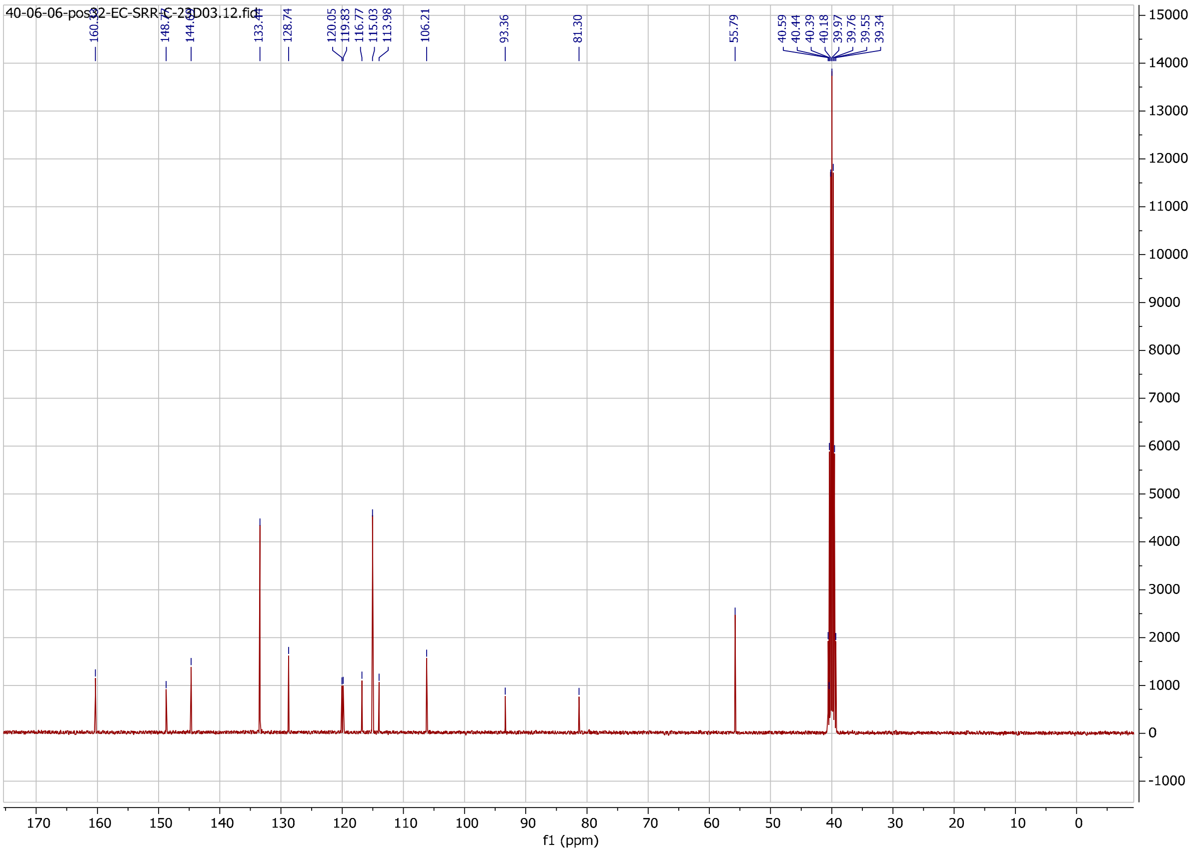

4-((1*H*-Indol-2-yl)ethynyl)aniline 35

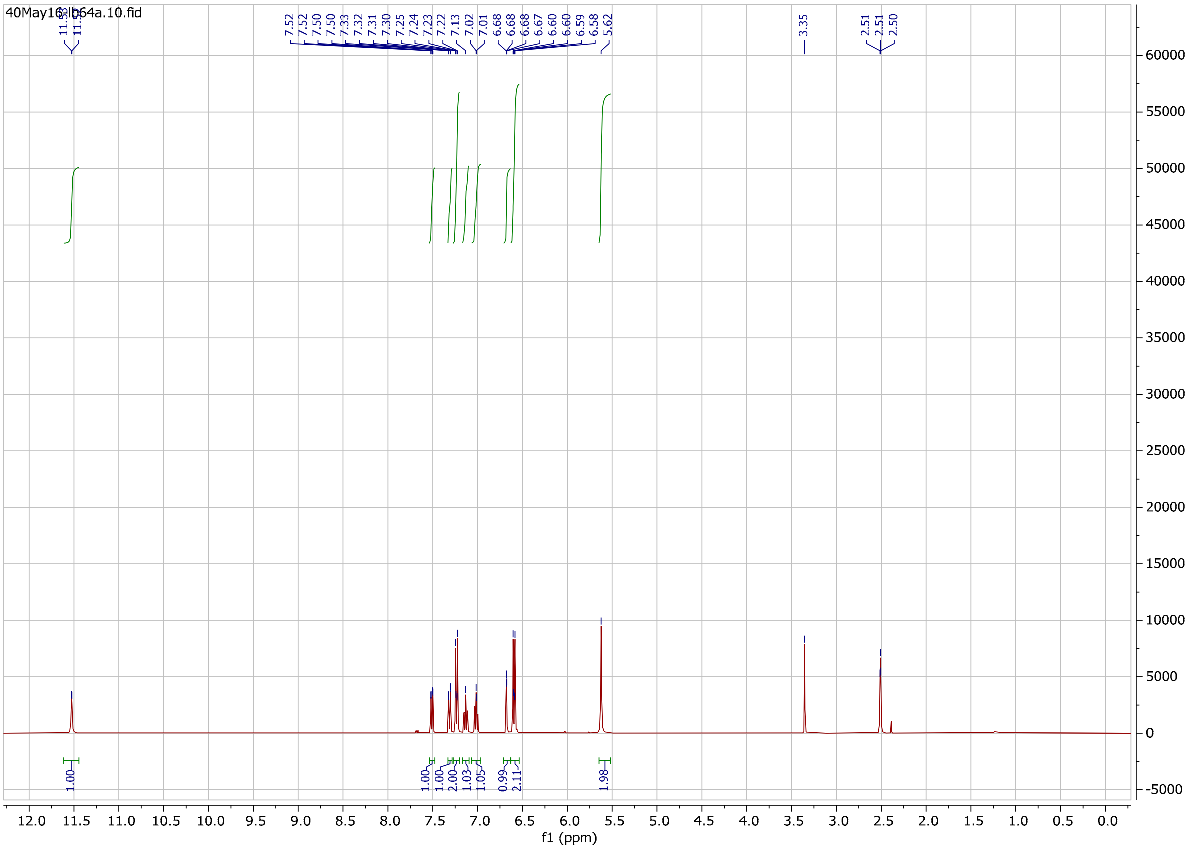

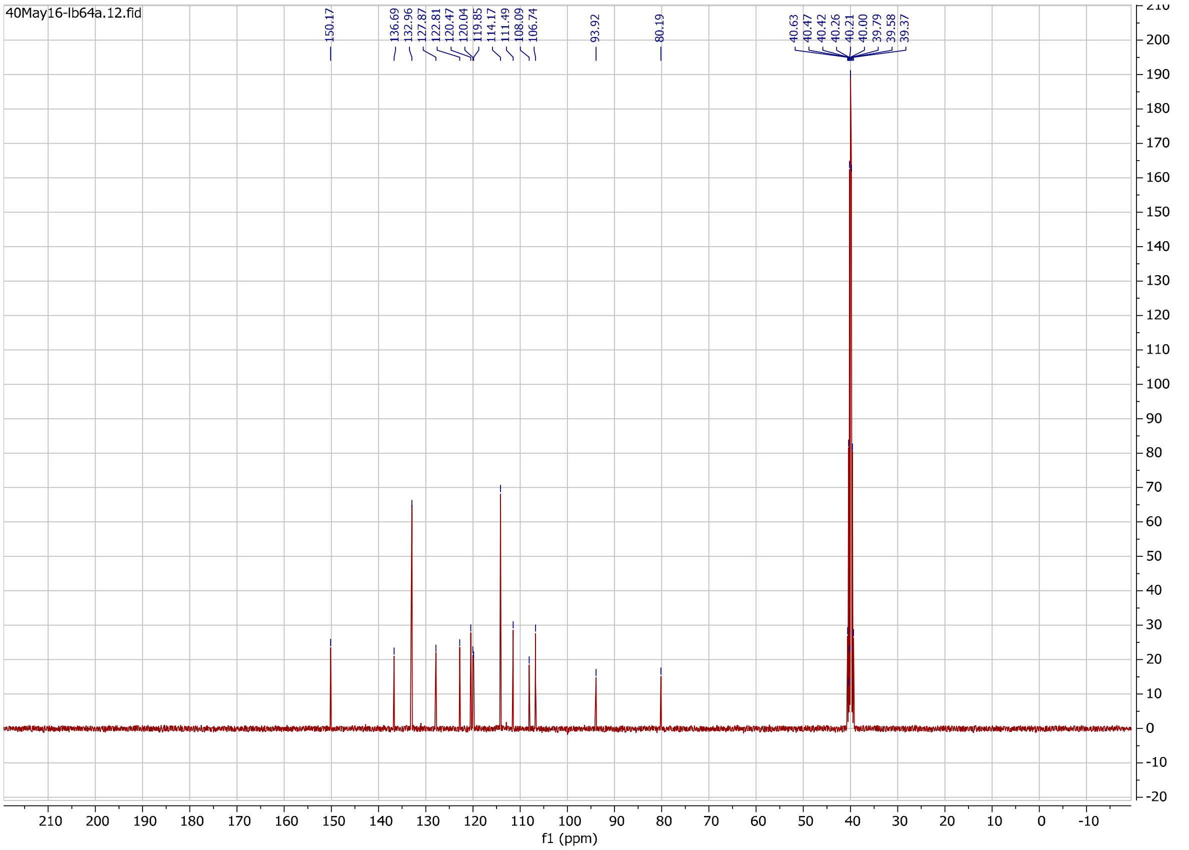

3-((1*H*-Indol-2-yl)ethynyl)aniline 36

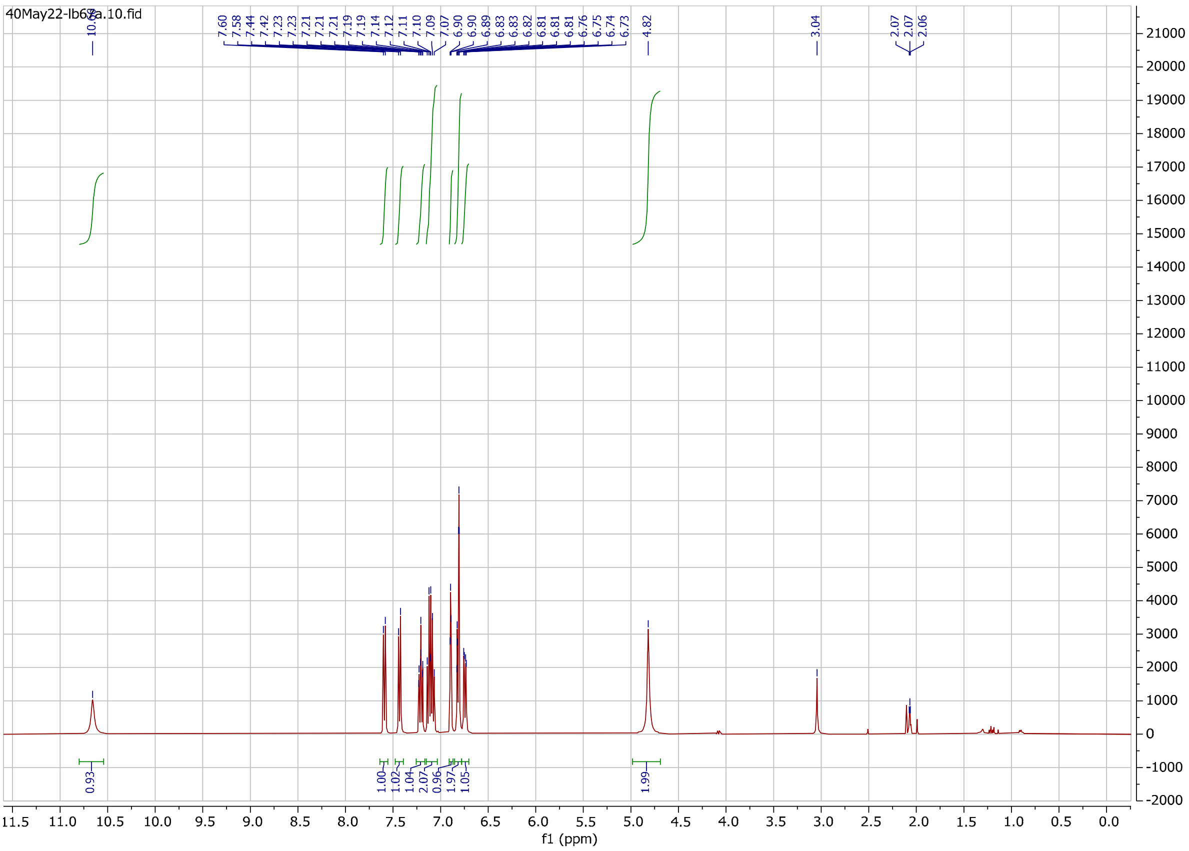

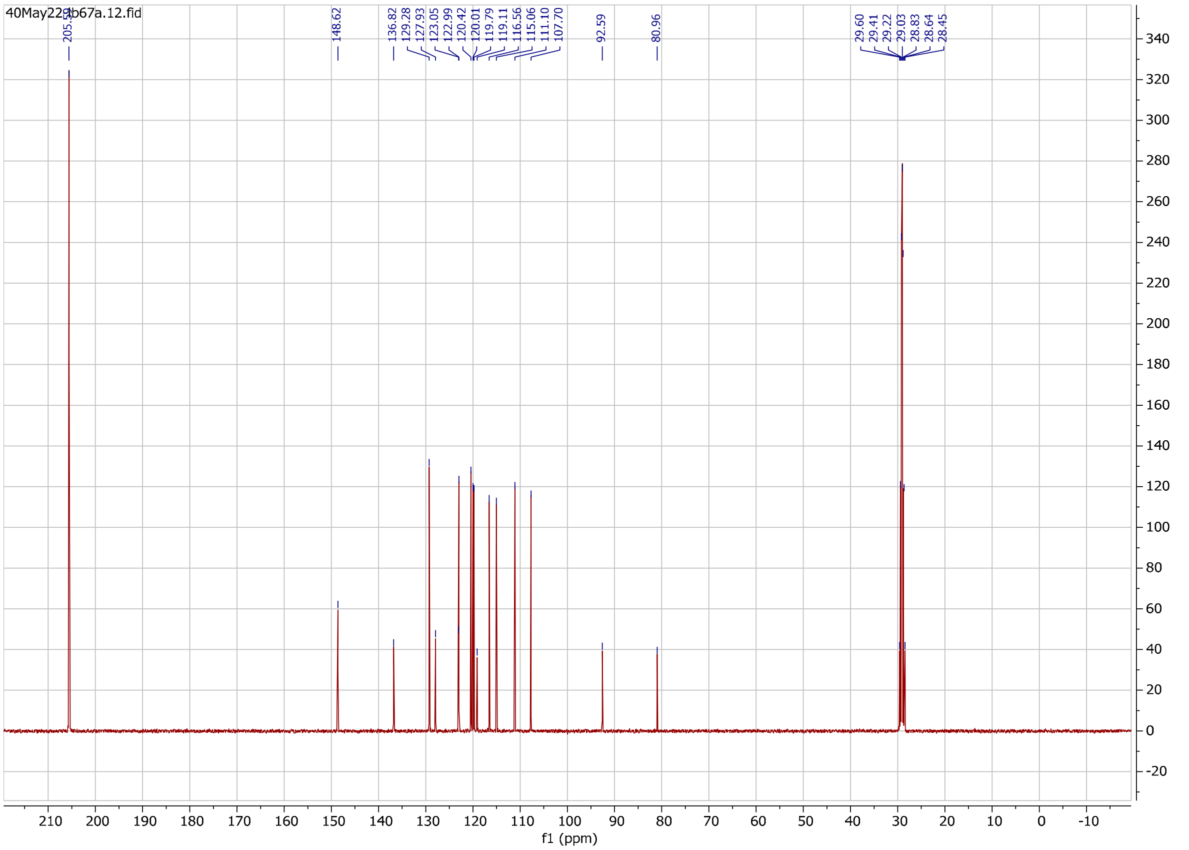

4-((1*H*-Indol-2-yl)ethynyl)-*N*-methylaniline 37

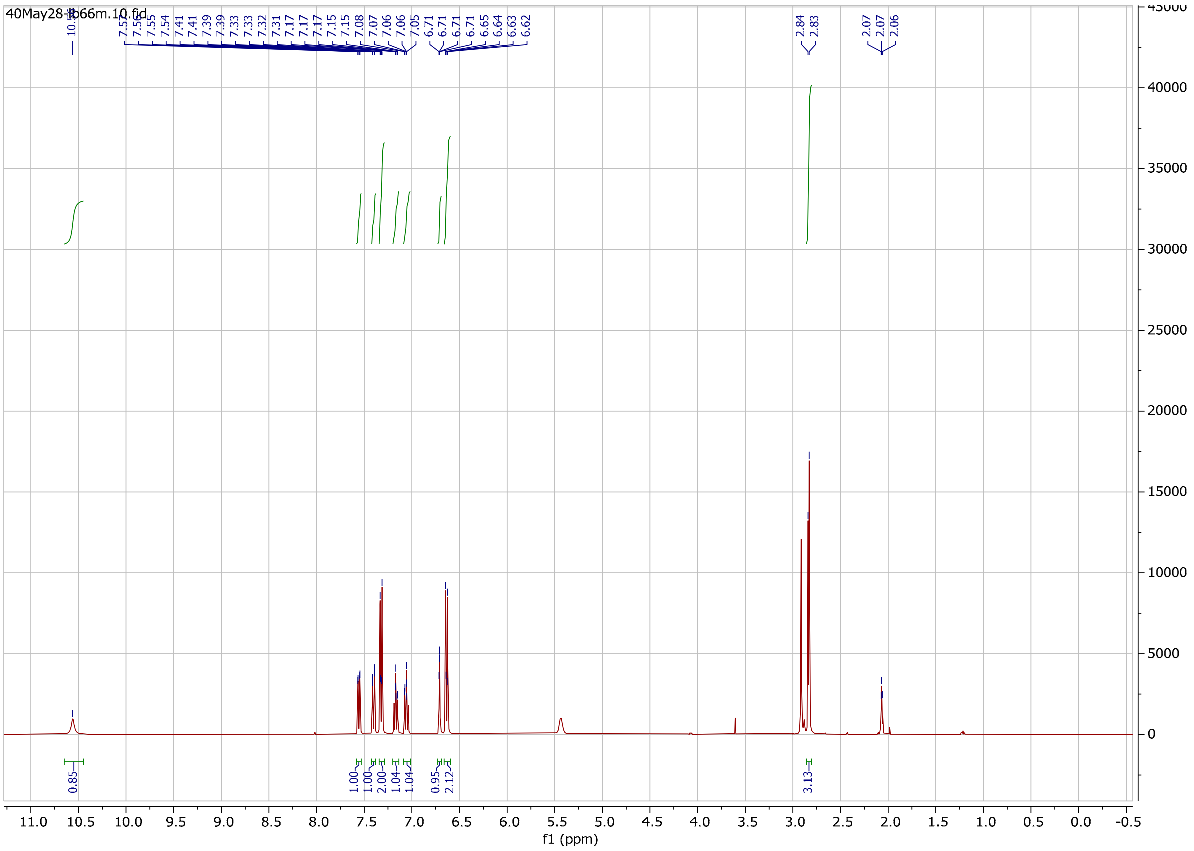

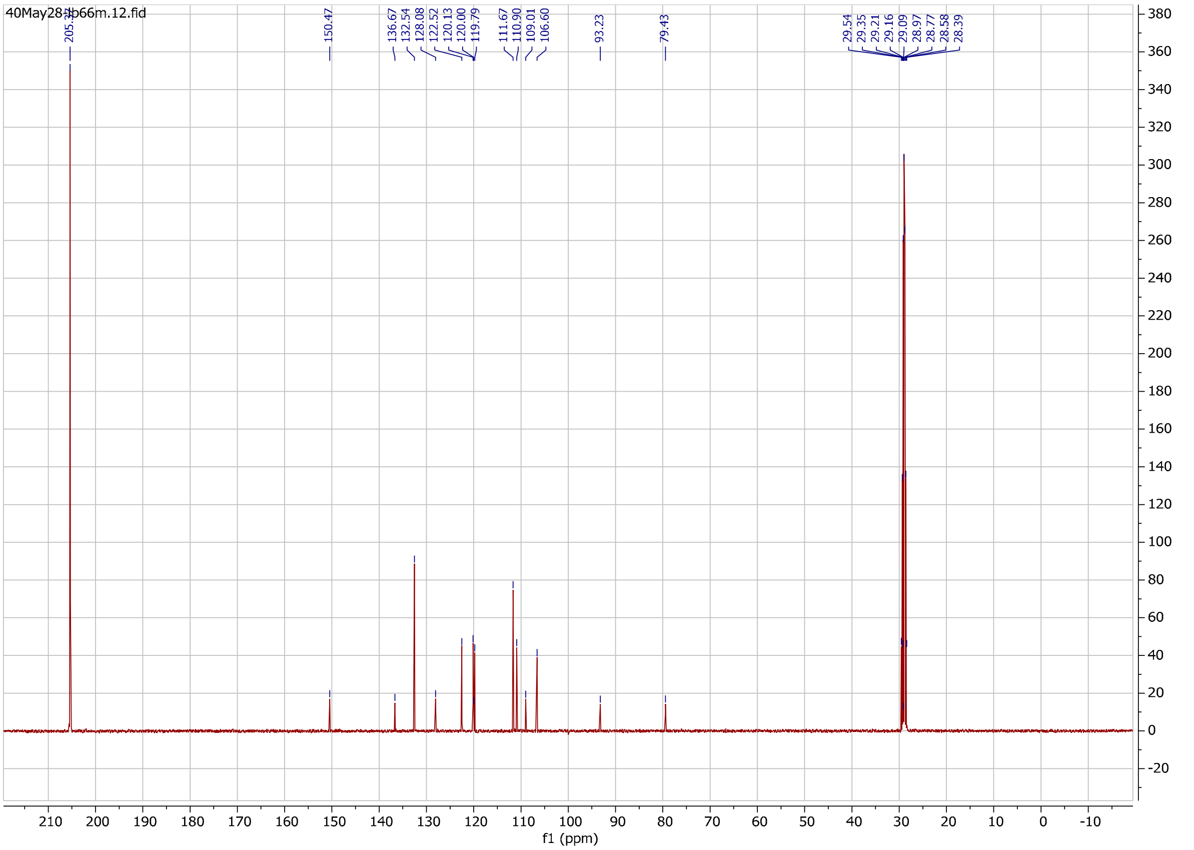

4-((1*H*-Indol-2-yl)ethynyl)-*N*,*N*-dimethylaniline 38

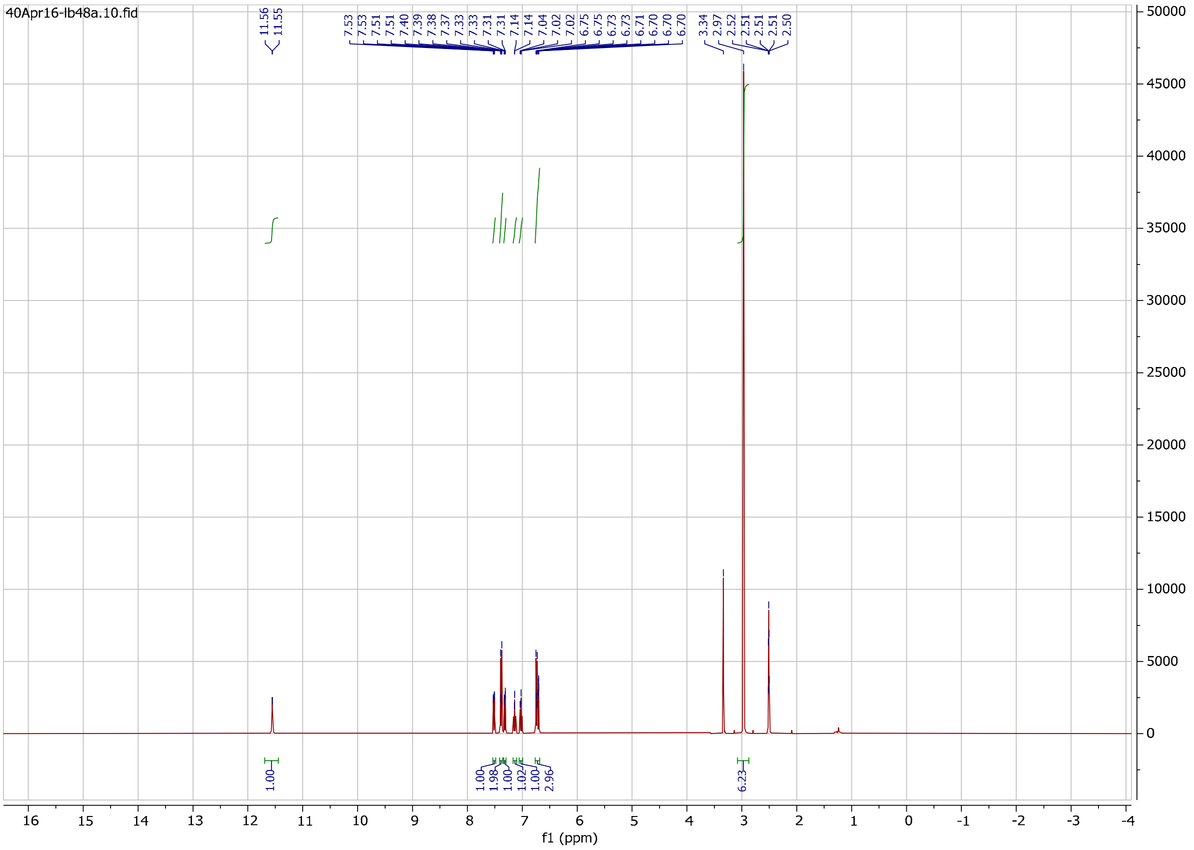

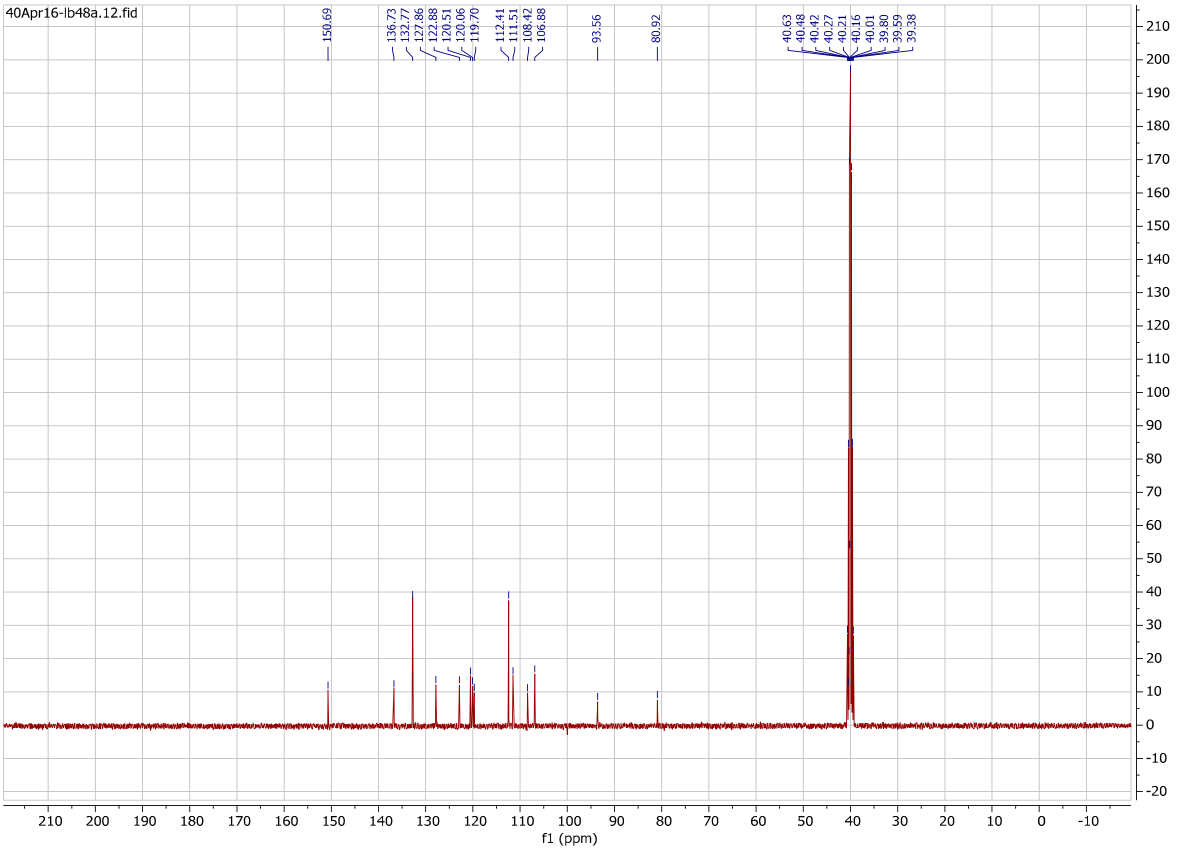

4-((5-Methoxy-1*H*-indol-2-yl)ethynyl)-*N*,*N*-dimethylaniline 39

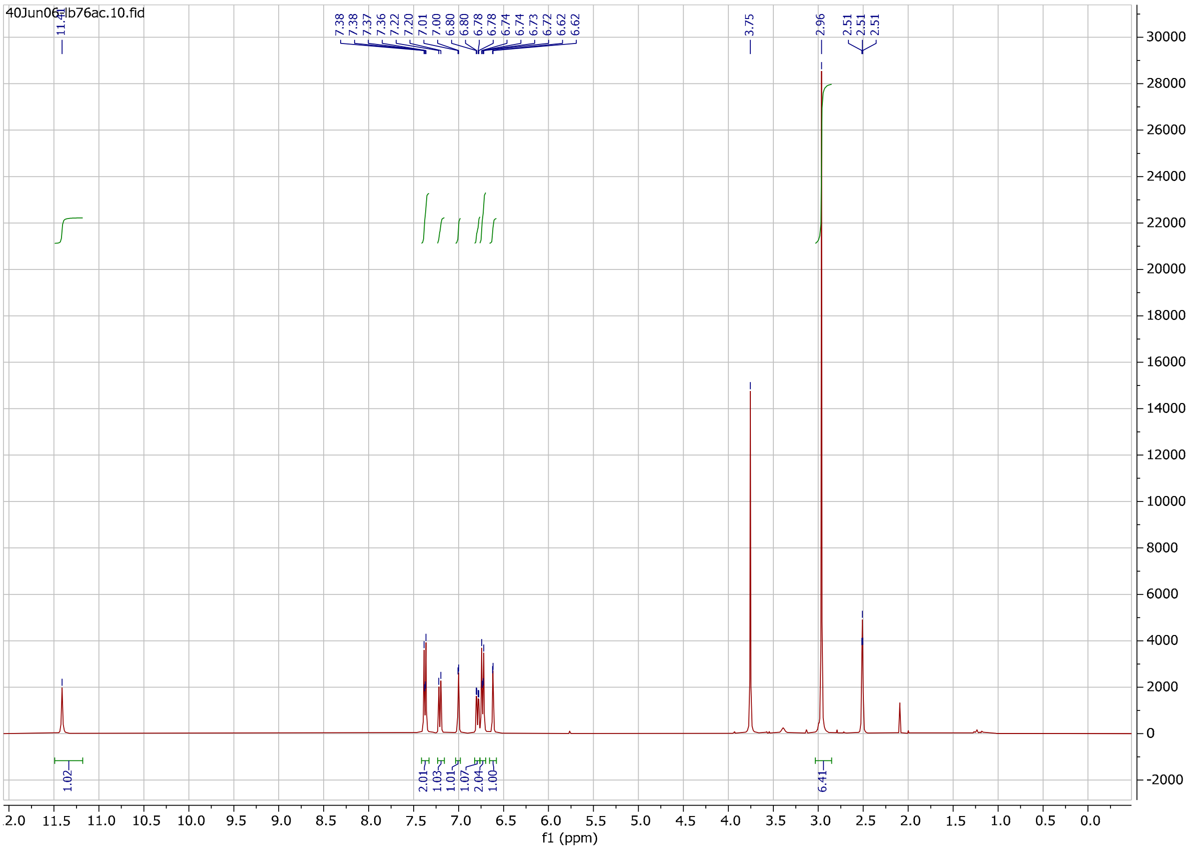

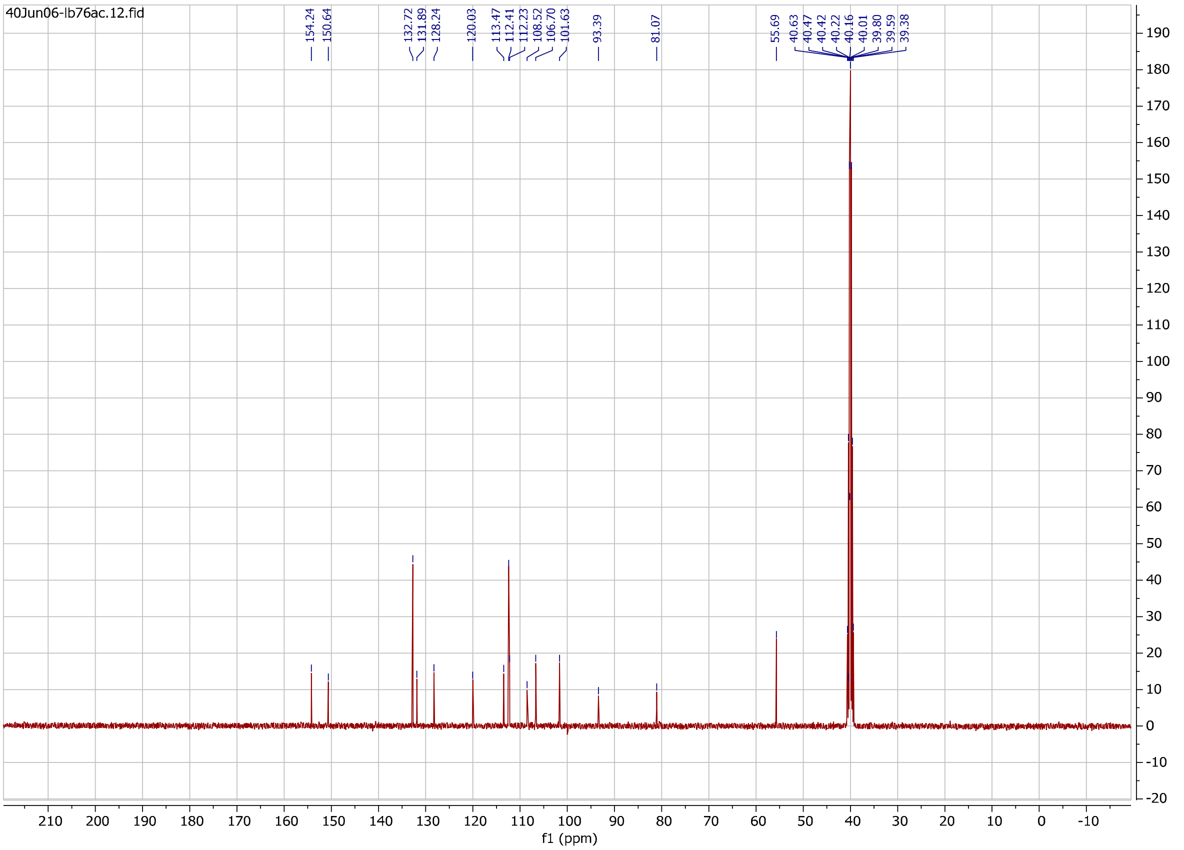

4-((5-(2-Fluoroethoxy)-1*H*-indol-2-yl)ethynyl)-*N,N*-dimethylaniline 40

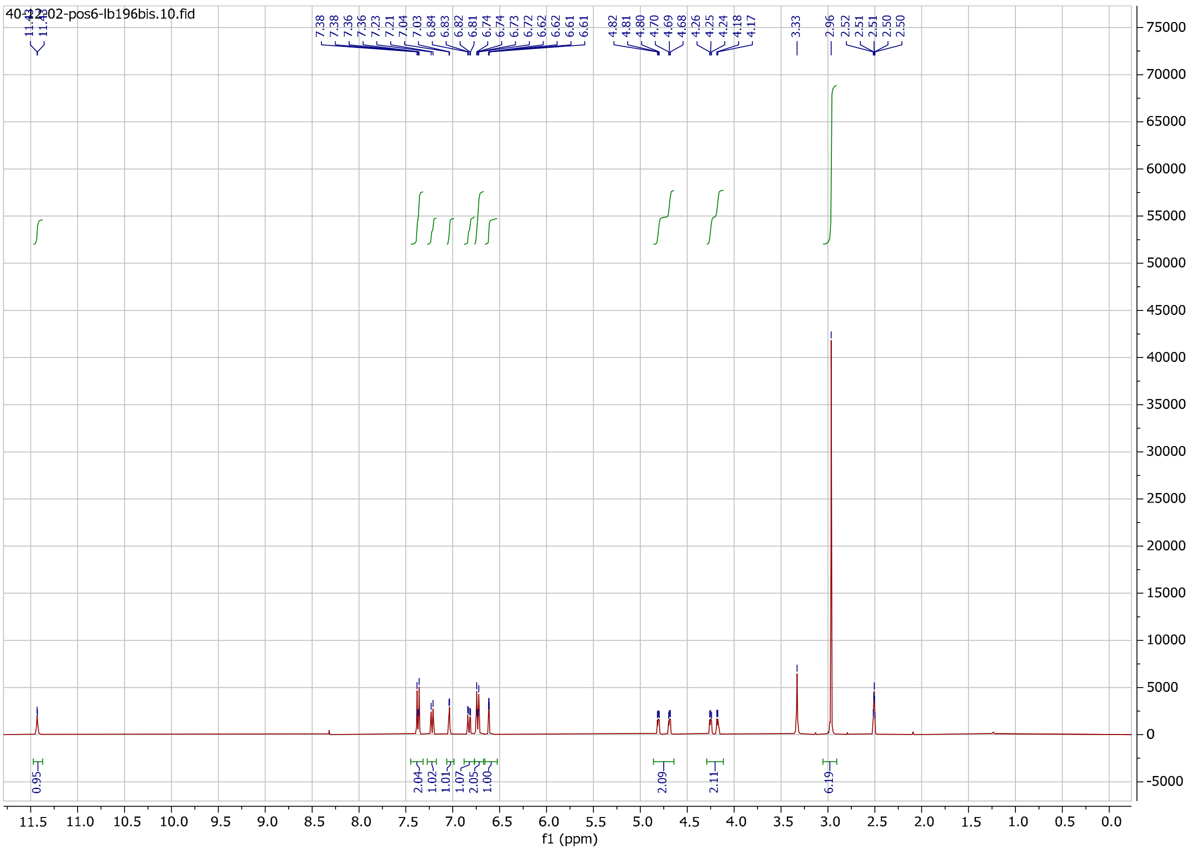

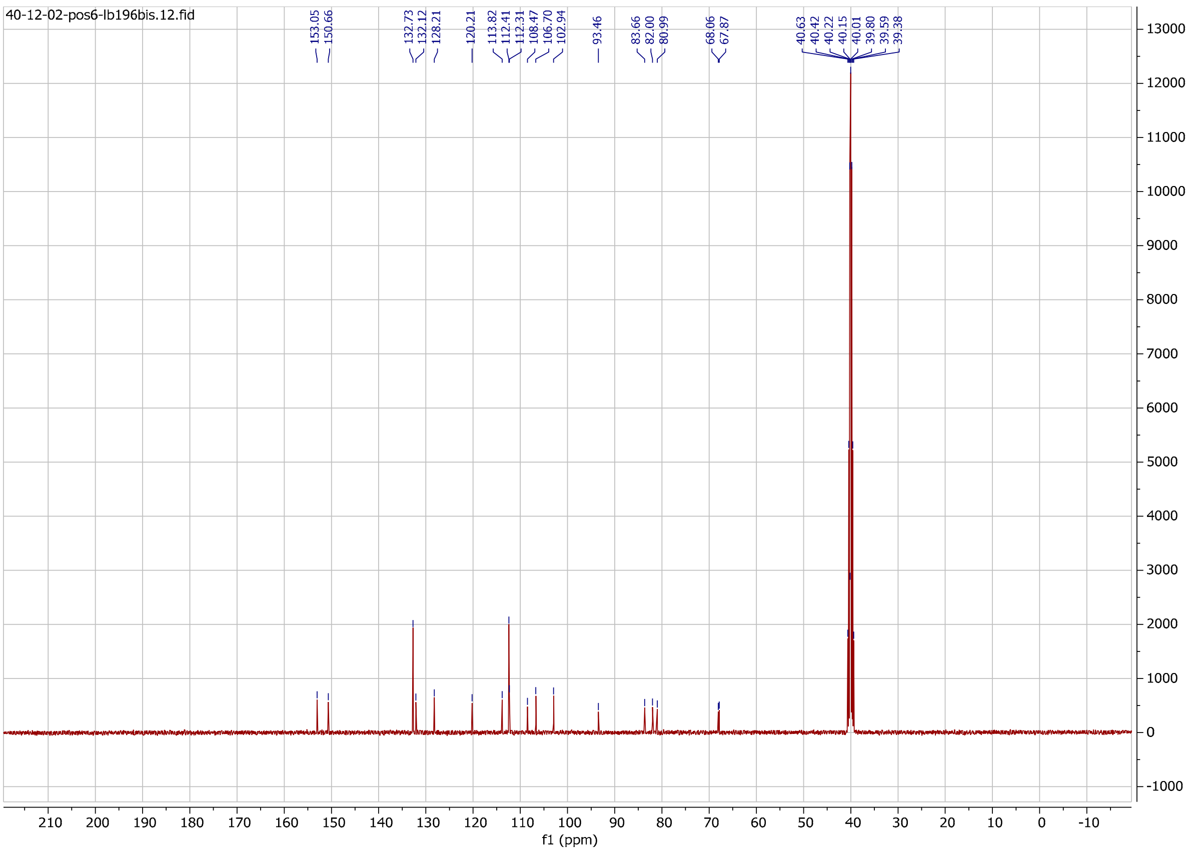

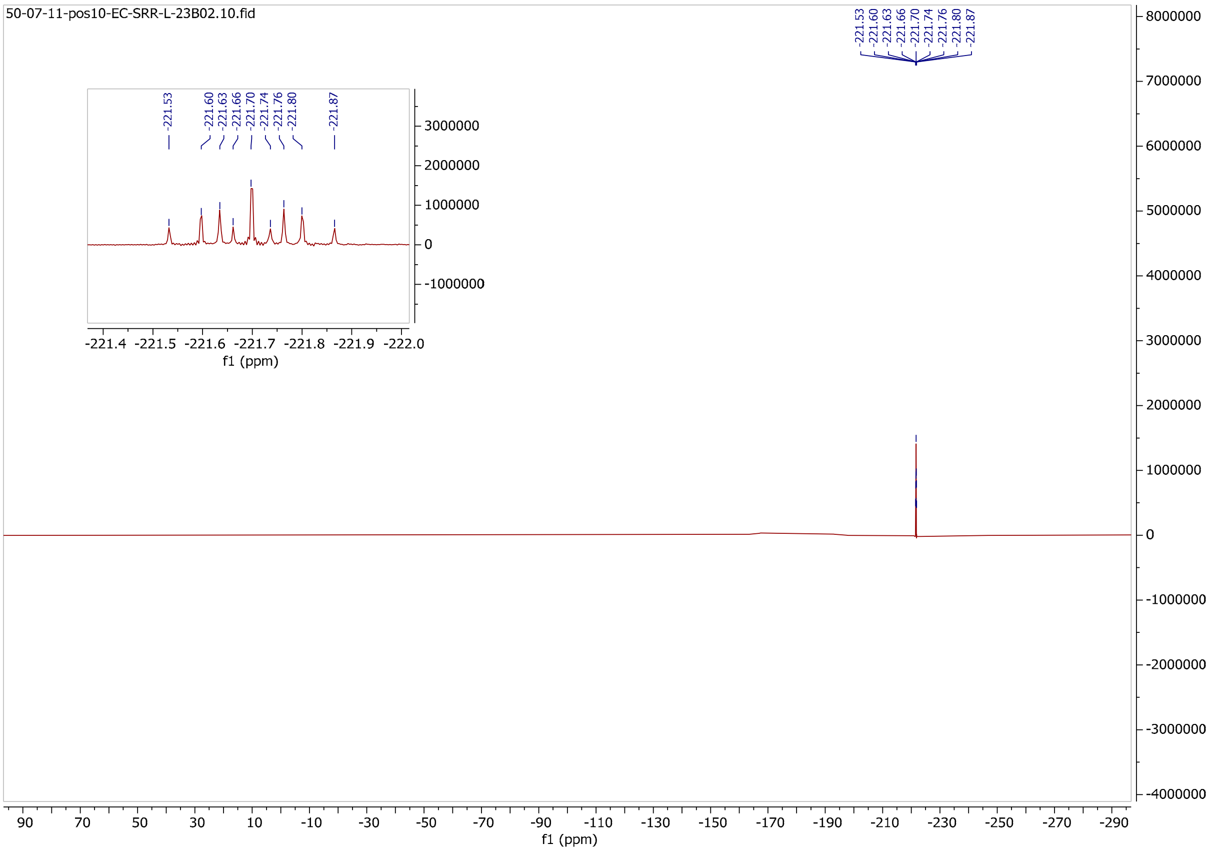
50-07-11-pos10-EC-SRR-L-23B02.10.fid
-221.53
-221.60
-221.63
-221.66
-221.70
-221.74
-221.76
-221.80
-221.87
f1 (ppm)
f1 (ppm)

4-((1*H*-Pyrrolo[3,2-*b*]pyridin-2-yl)ethynyl)-*N*,*N*-dimethylaniline 41

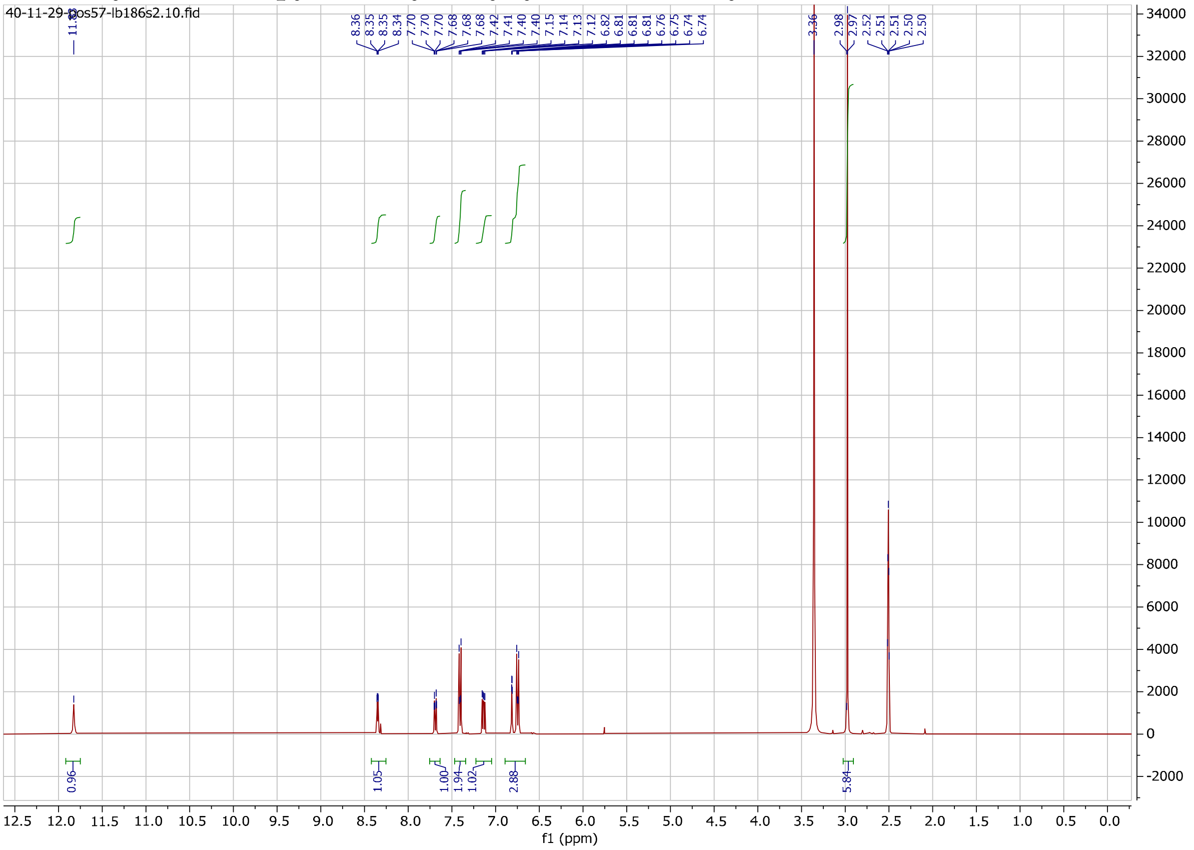

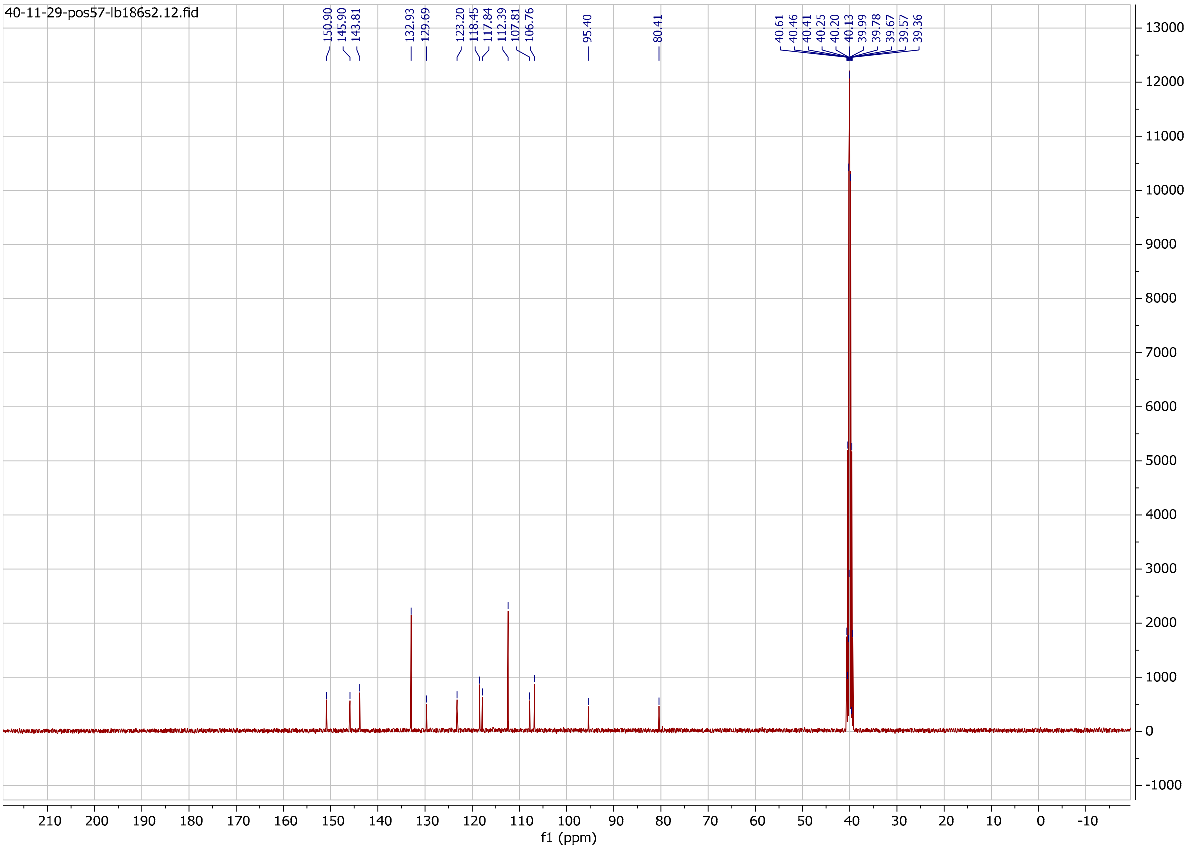

4-((1*H*-Pyrrolo[2,3-*c*]pyridin-2-yl)ethynyl)-*N*,*N*-dimethylaniline 42

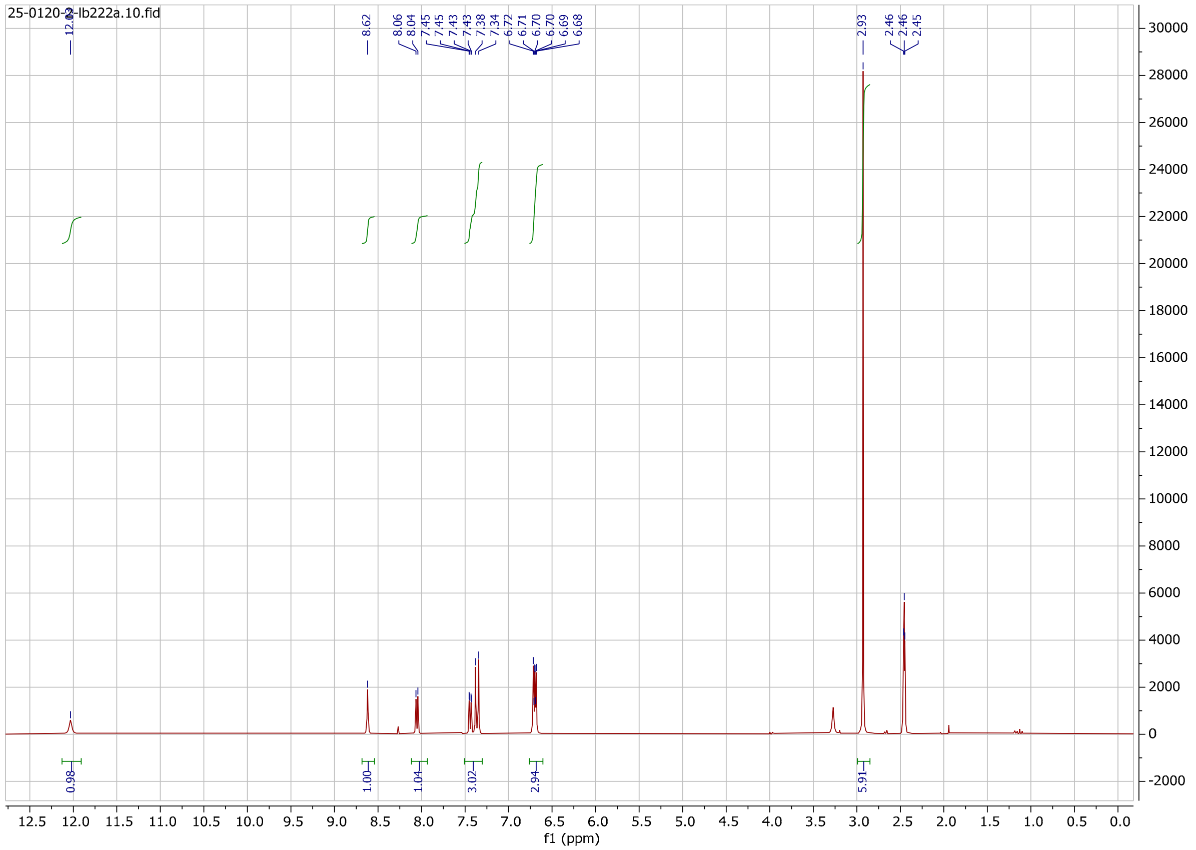

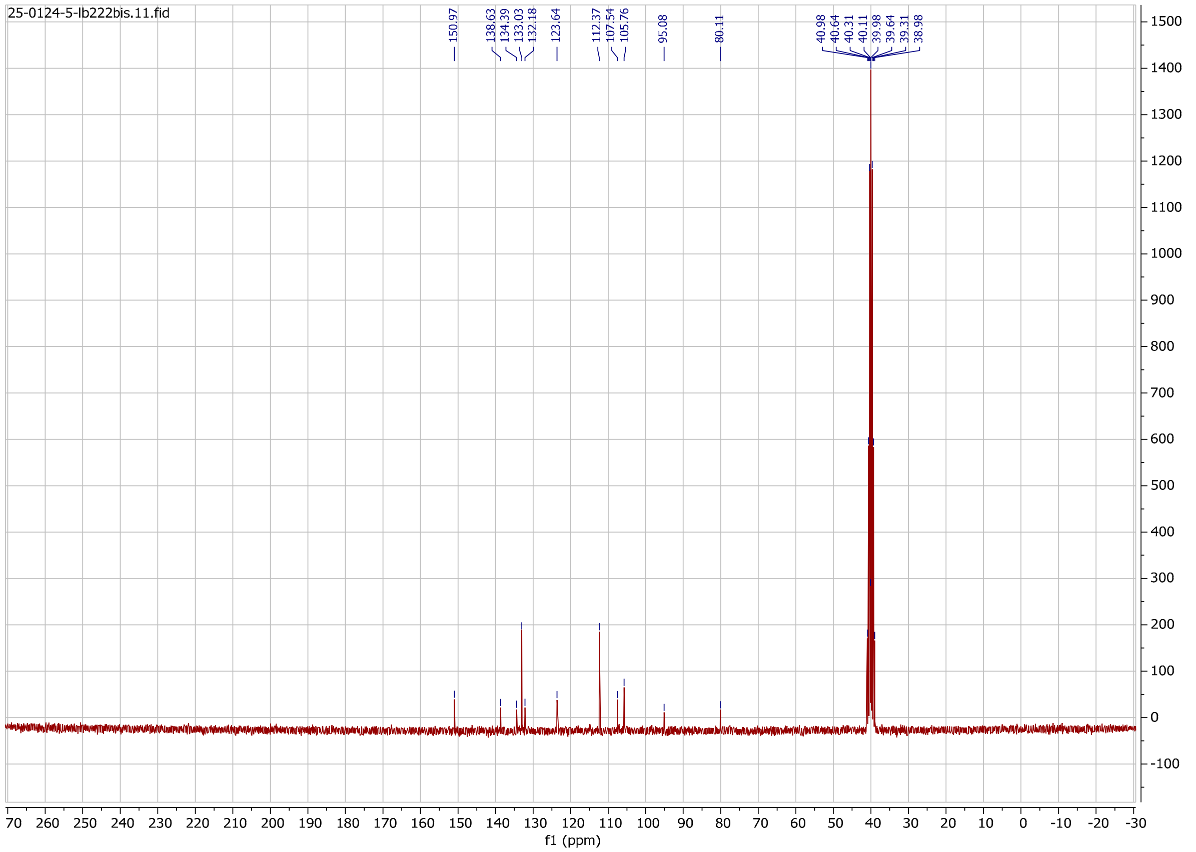

4-((1*H*-Pyrrolo[2,3-b]pyridin-2-yl)ethynyl)-*N,N*-dimethylaniline 43

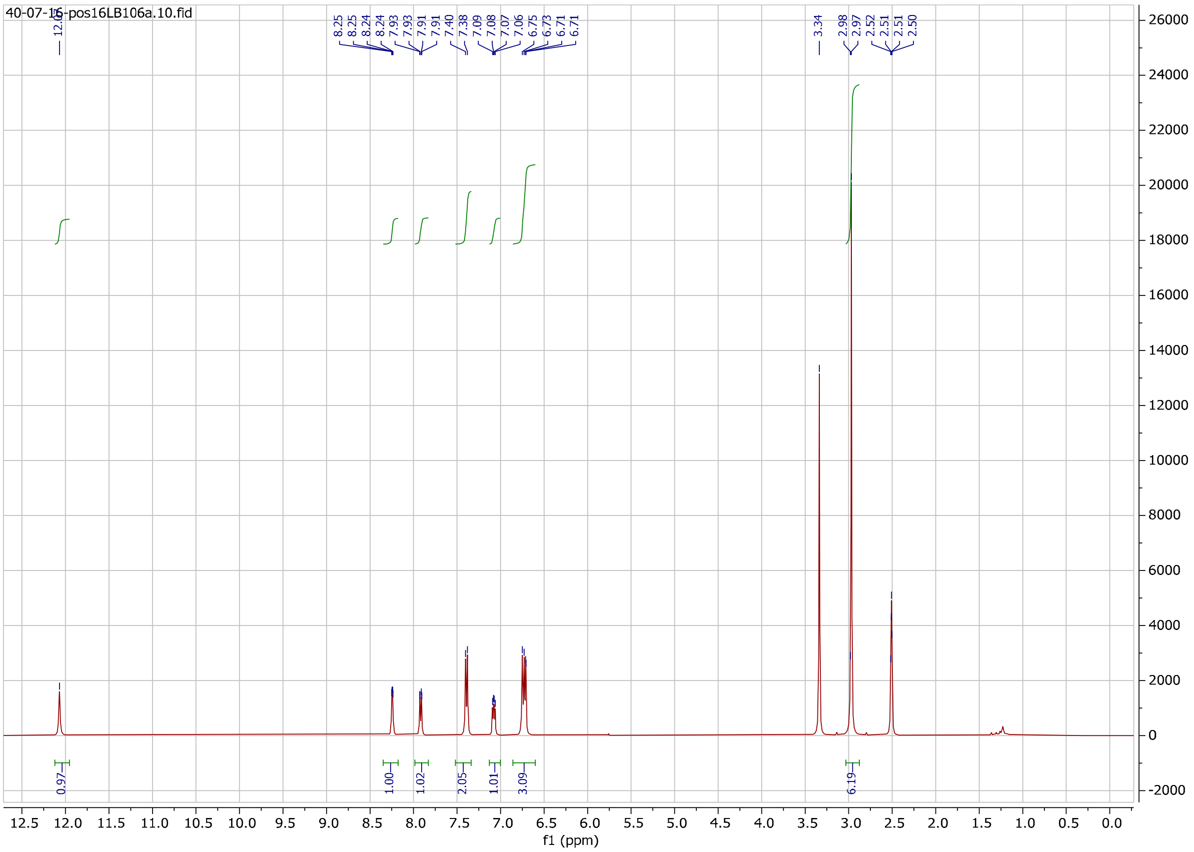

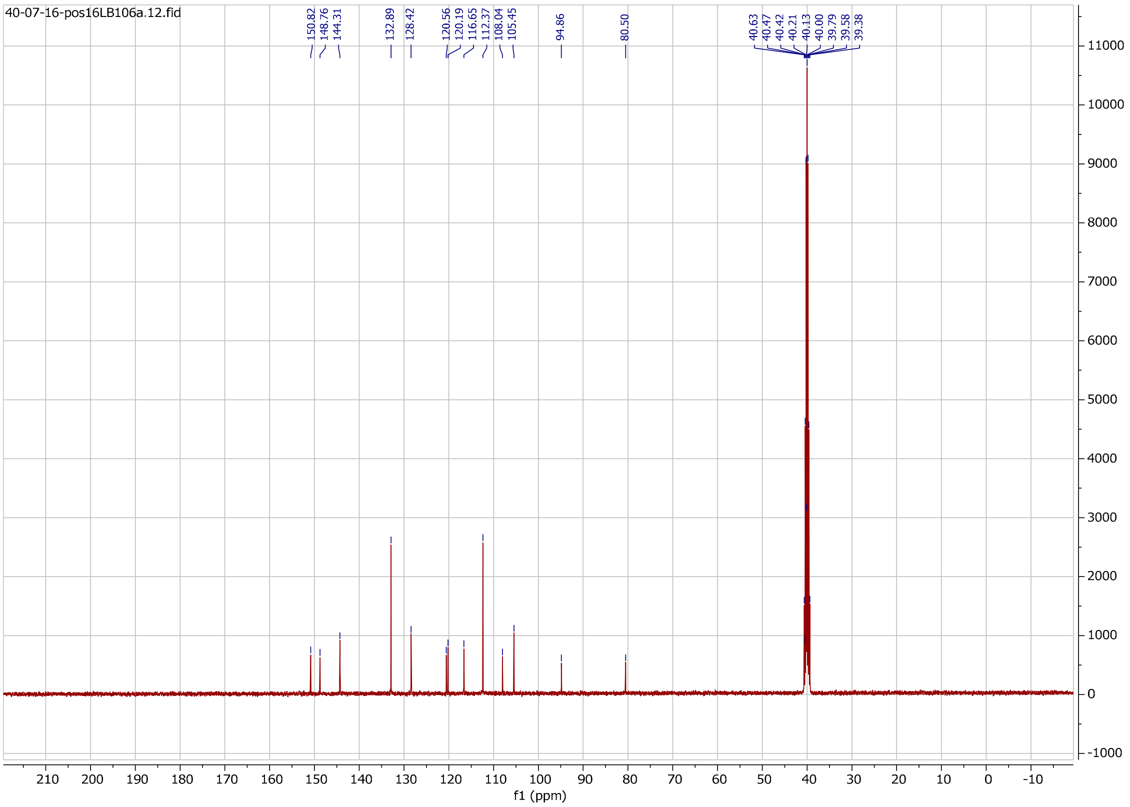

4-((1*H*-Pyrrolo[3,2-*c*]pyridin-2-yl)ethynyl)-*N*,*N*-dimethylaniline 44

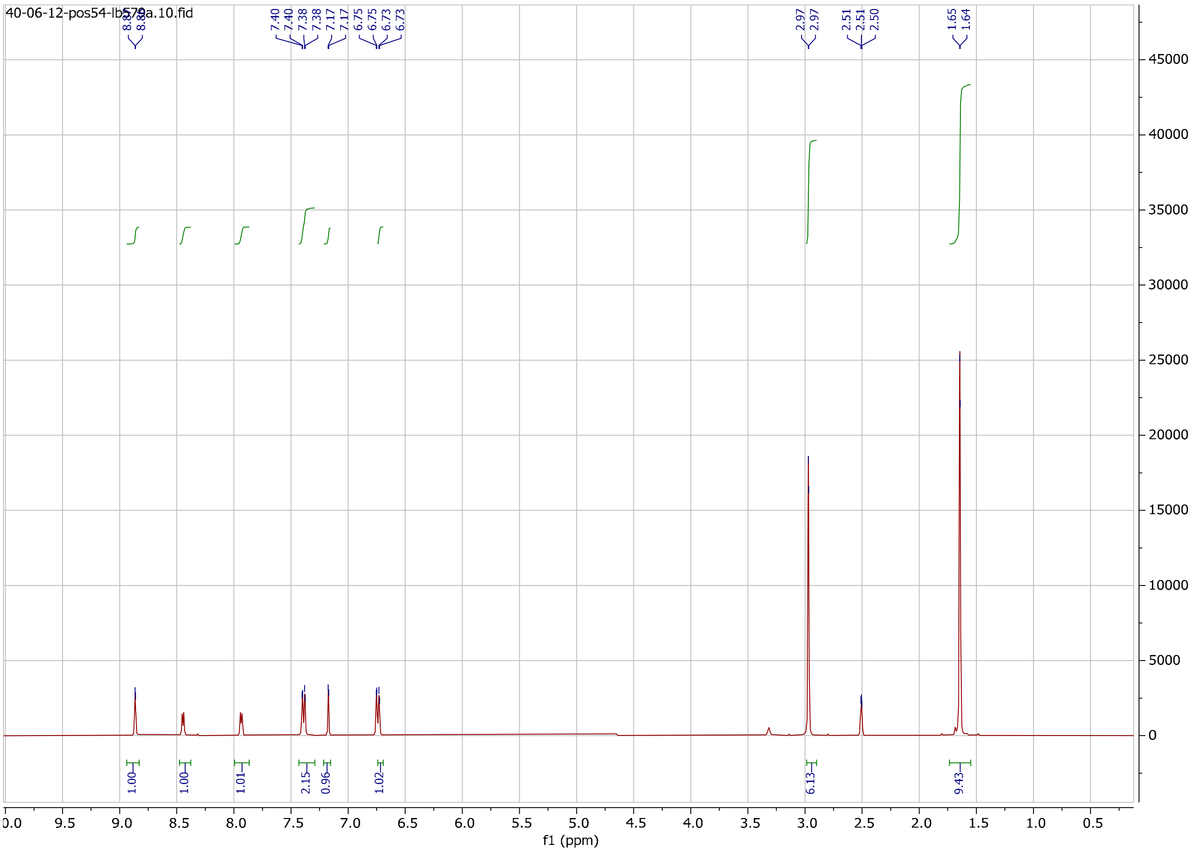

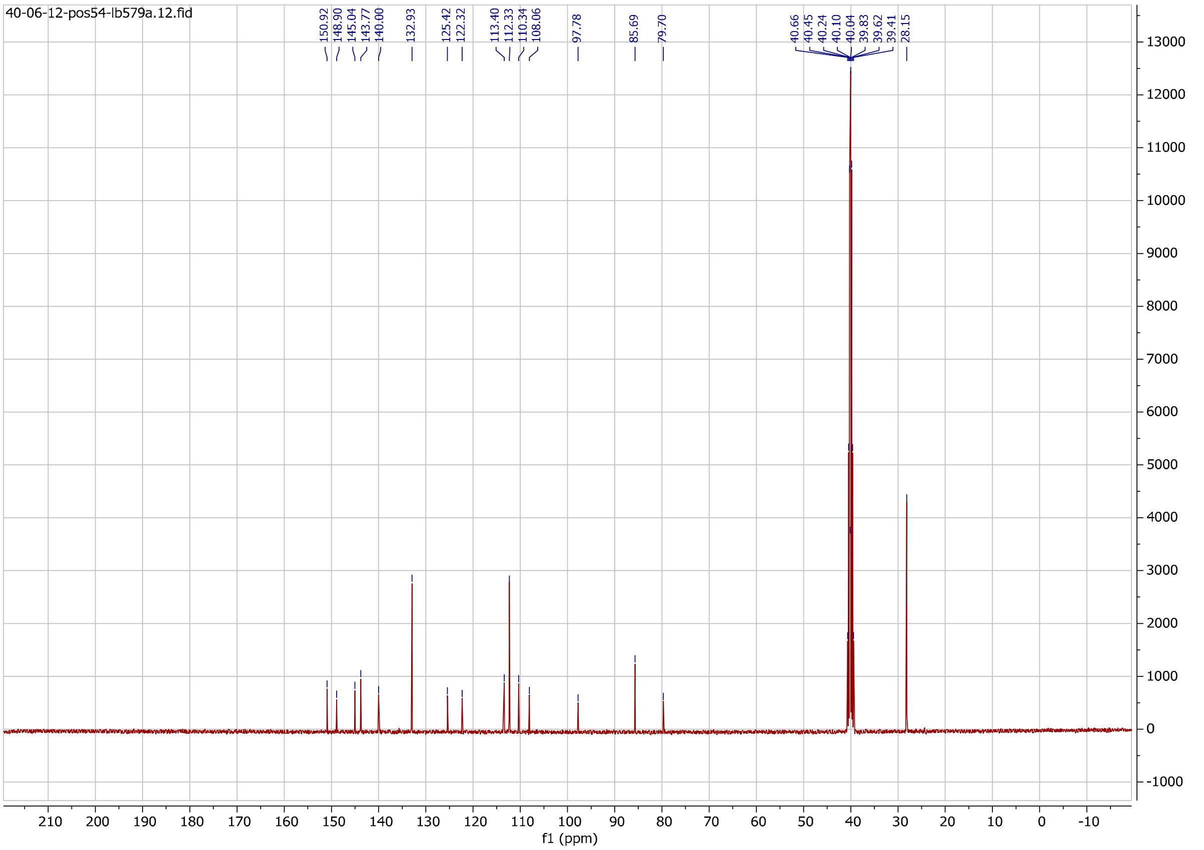

4-((1*H*-Pyrrolo[2,3-*b*]pyridin-2-yl)ethynyl)-*N*-(2-fluoroethyl)-*N*-methylaniline 45

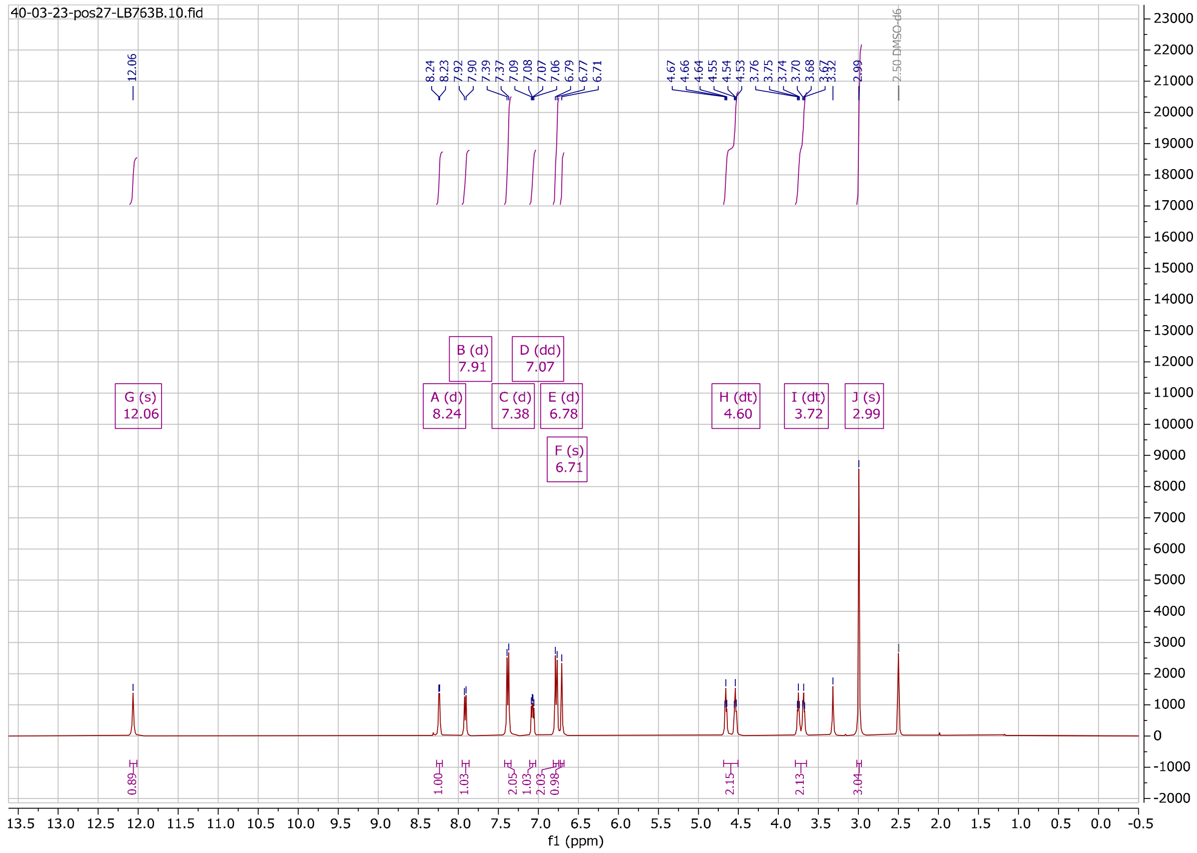

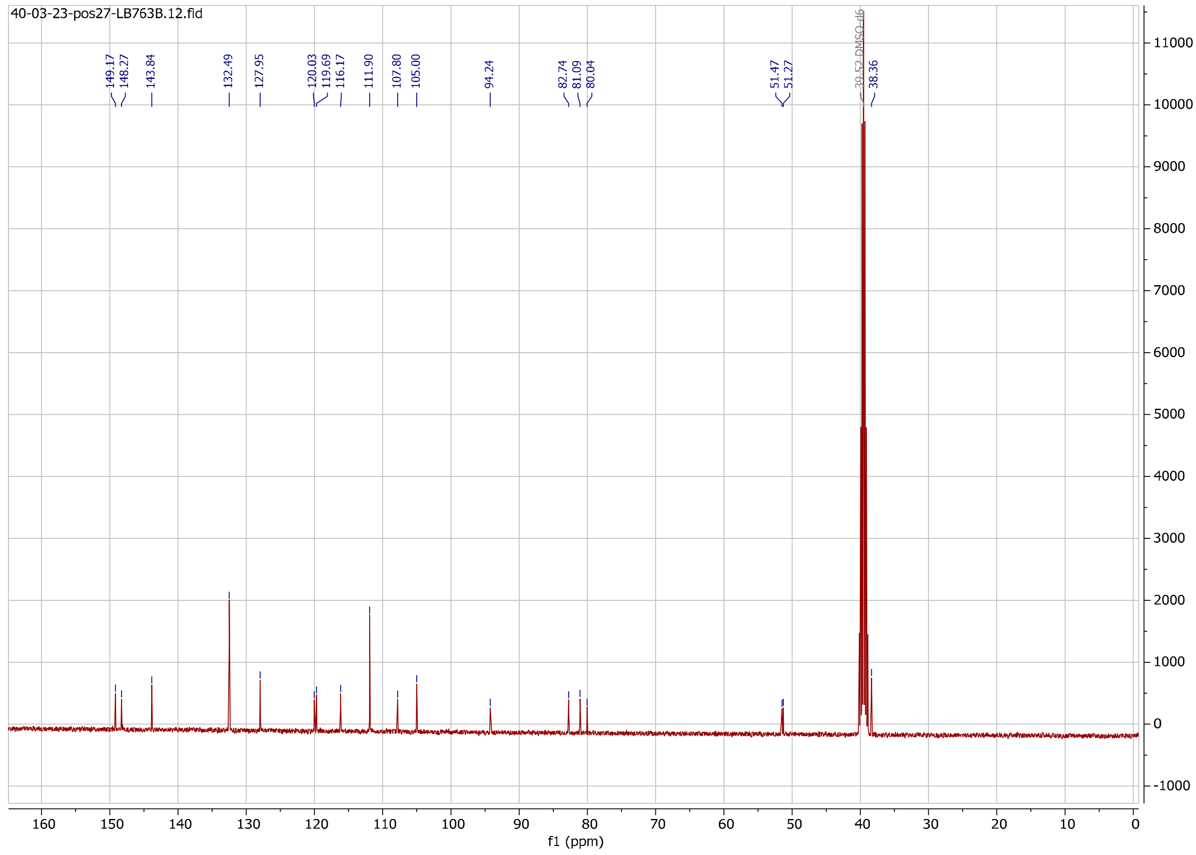

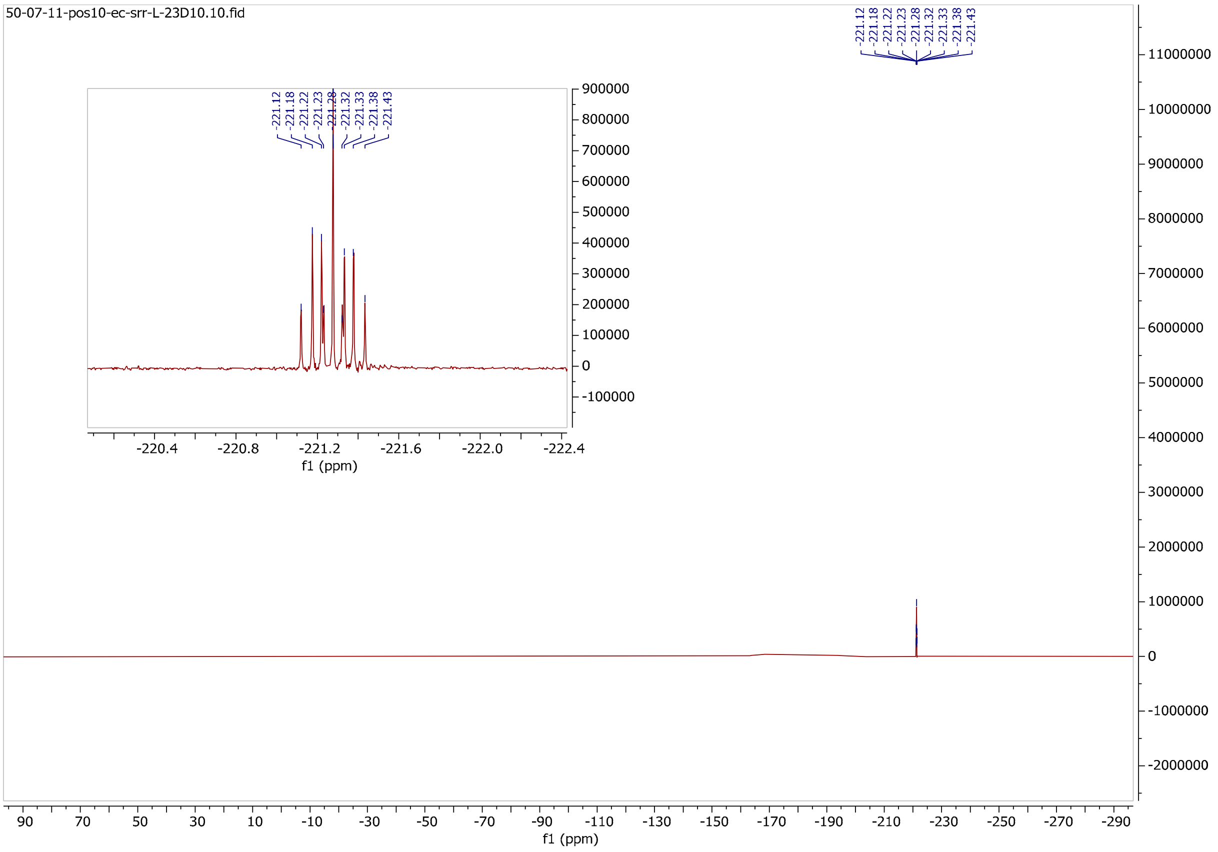
50-07-11-pos10-ec-srr-L-23D10.10.fid
-221.12
-221.18
-221.22
-221.23
-221.28
-221.32
-221.33
-221.38
-221.43
f1 (ppm)
f1 (ppm)

tert-butyl 2-((4-(méthyl(2-(tosyloxy)éthyl)amino)phényl)éthynyl)-1*H*-pyrrolo[2,3-*b*]pyridine-1-carboxylate 46

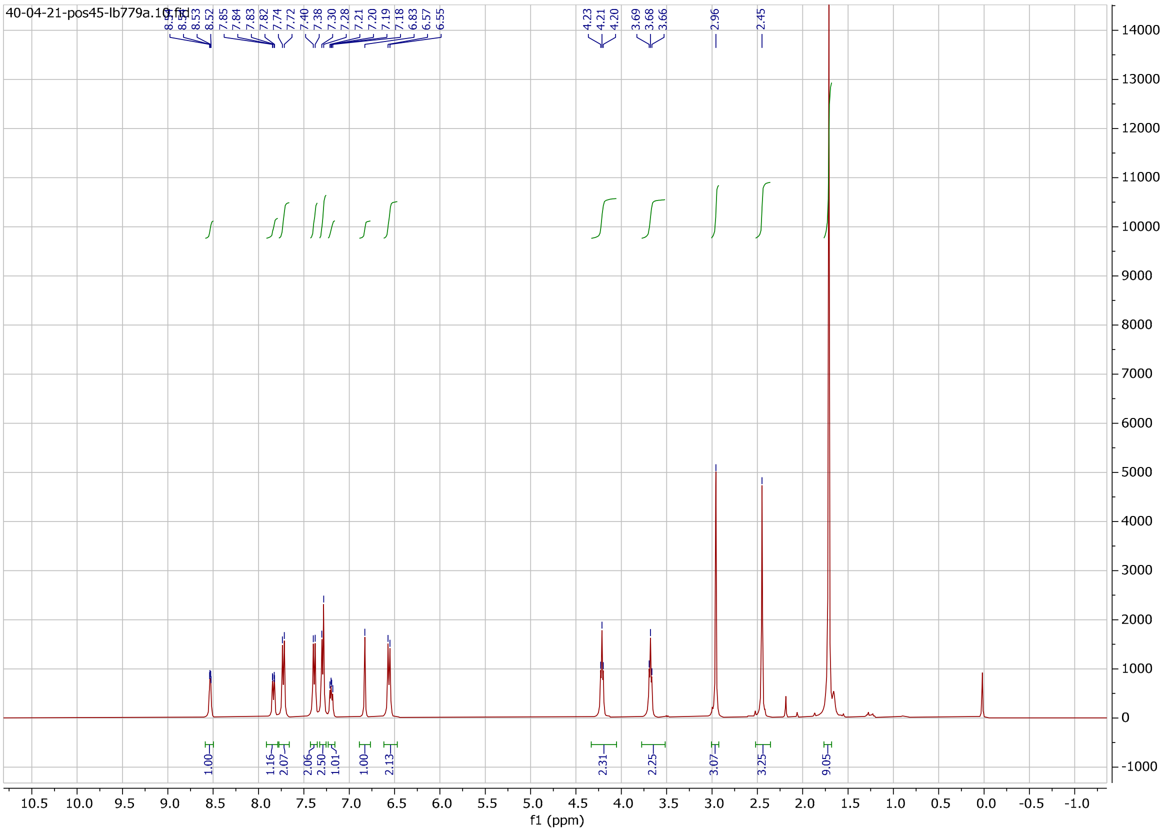

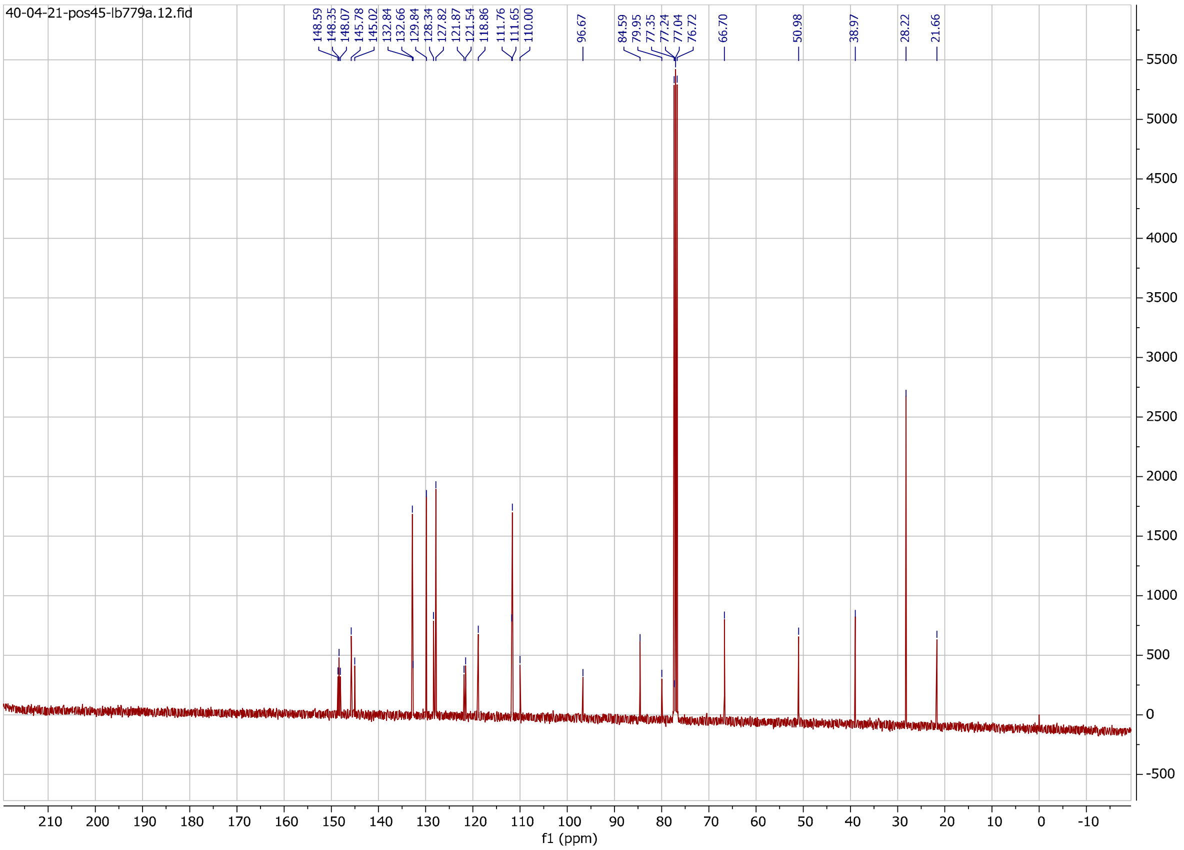

Determination of molar activity: Method and HPLC data.

Calibration curve was performed with nine different concentrations from 1 to 50 µg/10 mL. The concentration values were: 1;2;4;8;12;16;20;25 and 50 µg/10 mL. Measures were repeated five times for each concentration and allow the drawing of the following calibration curve. The coefficient determination was higher than 99.9 % and the estimated slope was 0.362

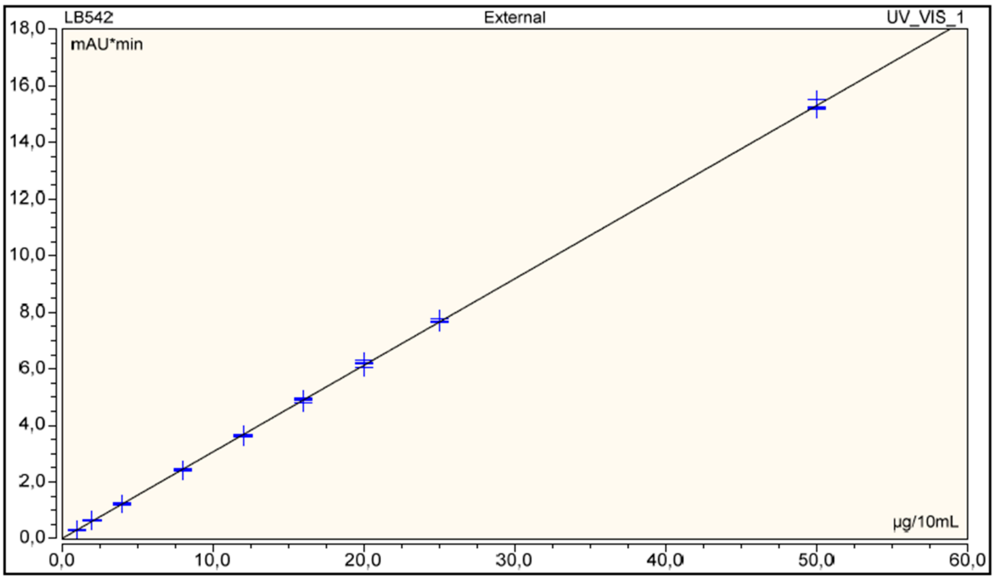

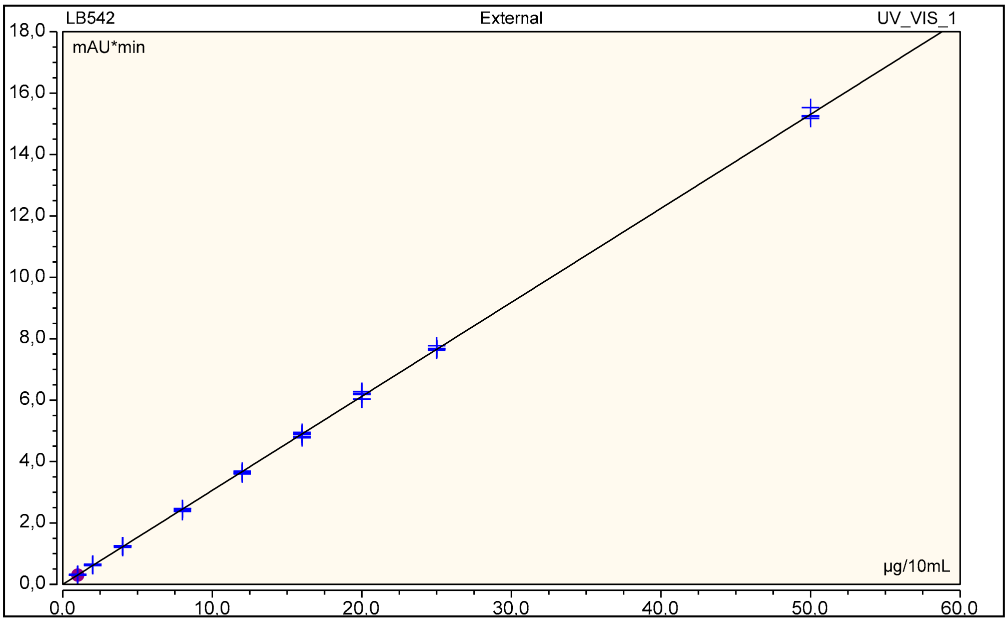

No.	Ret.Time min	Peak Name	Cal.Type	Points	Coeff.Det. %	Offset	Slope	Curve
1	3.63	LB542	Lin	45	99.9778	0.0000	0.3062	0.0000
**Average:**					99.8709	0.0000	0.3062	0.0000

HPLC purity of compound [^{18}F]45.

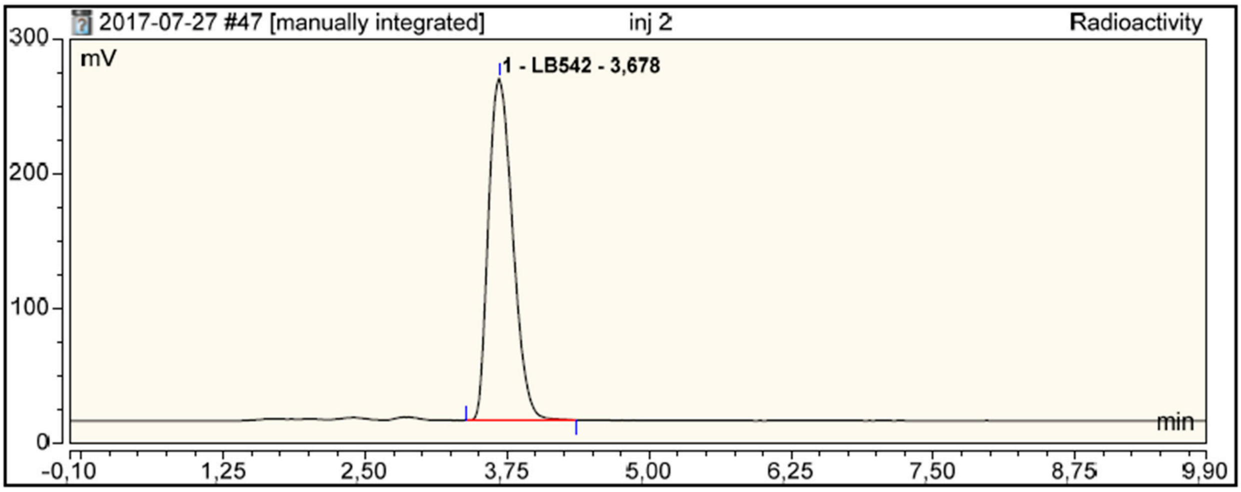

HPLC purity of compound 45.

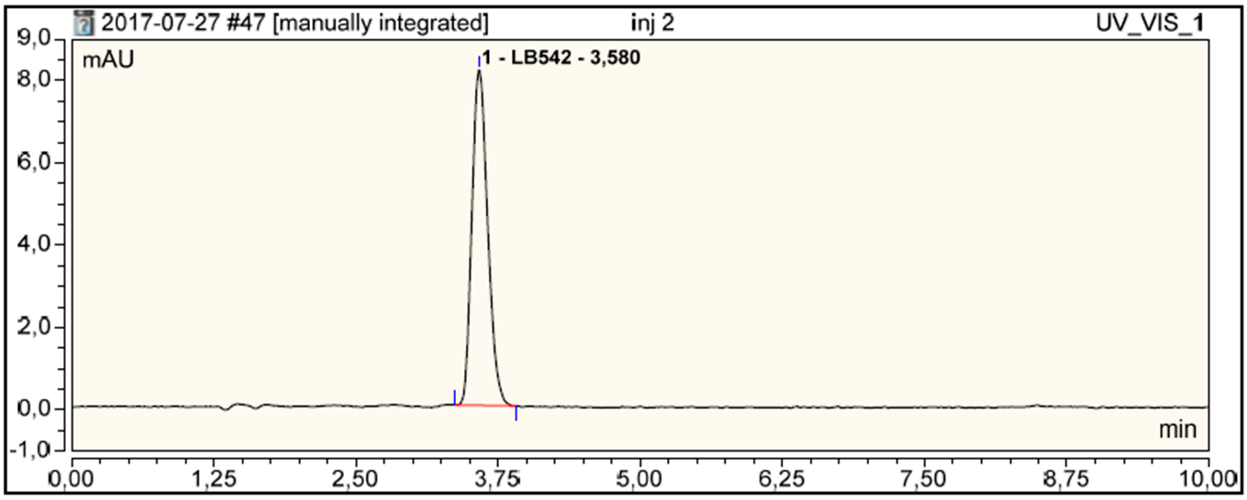

Selection of microPET images used to obtain the quantitative data 3.

Coronal and sagittal fused static brain PET images (40–180 min post-tracer injection) with the MRI T2 template (PMOD v3.4). Regions of interest : IST, CST and CE

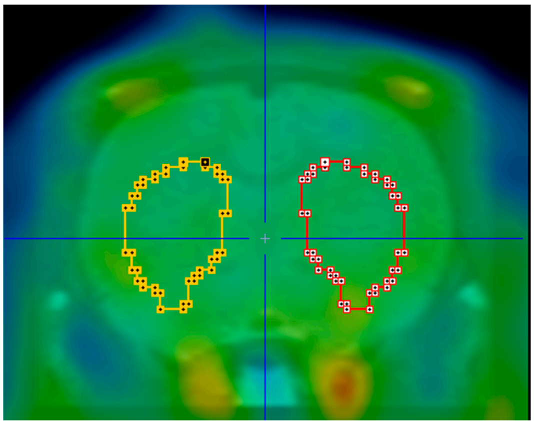

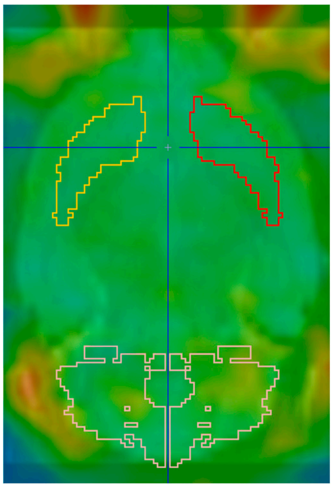

Supplement: Supplementary file 1 [file pharmaceuticals-18-01638-s001.zip › pharmaceuticals-3884275-supplementary.pdf]
